# Investigating the effects of missense mutations on the dual Ras/Rap activation mechanism of SYNGAP1 using molecular modelling

**DOI:** 10.1016/j.yjsbx.2026.100162

**Published:** 2026-09-10

**Authors:** Aliaa E. Ali, Arto Liljeblad, Jukka V. Lehtonen, Noboru H. Komiyama, Pekka A. Postila

**Affiliations:** a Institute of Biomedicine, Integrative Physiology and Pharmacology, University of Turku, FI-20014 Turku, Finland; b InFLAMES Research Flagship, University of Turku, 20014 Turku, Finland; c Structural Bioinformatics Laboratory, Department of Natural and Health Sciences, Åbo Akademi University, FI-20500 Turku, Finland; d InFLAMES Research Flagship, Åbo Akademi University, FI-20500 Turku, Finland; e Institute for Neuroscience and Cardiovascular Research, University of Edinburgh, Edinburgh EH16 4SB, UK; f Simons Initiative for the Developing Brain (SIDB), Centre for Discovery Brain Sciences, University of Edinburgh, Edinburgh EH8 9XD, UK; g The Patrick Wild Centre, Centre for Discovery Brain Sciences, University of Edinburgh, Edinburgh EH8 9XD, UK.

**Keywords:** *SYNGAP1*, SynGAP, Ras-GTPase, Rap1b-GTPase, Activation mechanism, Missense mutation, Tumor suppressor, SynGAP-related disorder (SRD), Intellectual disability, Molecular dynamics (MD) simulations, Binding free energy calculations

## Abstract

SynGAP (Synaptic GTPase-Activating Protein) is enriched in the postsynaptic density, where it accelerates GTP hydrolysis by Ras- and Rap-GTPases. In forebrain, SynGAP is essential for synaptic plasticity and cognitive development, and de novo mutations in *SYNGAP1* gene cause SynGAP-related disorder (SRD), characterized by intellectual disability, autism, and epilepsy. Beyond the nervous system, SynGAP regulates cell growth, differentiation, and cytoskeletal dynamics, and loss of its GAP activity has been implicated in tumorigenesis and cancer metastasis. Although classified as a RasGAP based on homology, SynGAP efficiently promotes GTP hydrolysis of both Ras and Rap1b. Here, a combination of modelling approaches, including in silico mutagenesis, free-energy calculations, and atomistic molecular dynamics simulations, were used to define the structural basis of this dual specificity. Cancer-associated and SRD-associated missense variants were analyzed to assess their effects on SynGAP interactions with Ras and Rap and, by extension, on GAP-mediated GTP hydrolysis. Modelling suggests that SynGAP regulates Ras through a canonical RasGAP mechanism, whereas Rap1b is controlled through a Plexin-like mechanism involving opening of Rap-Switch II and engagement of a previously uncharacterized region termed the SynGAP activation-segment. The modelling supports the established role of the C2-domain residue Arg401, possibly stabilizing an open Rap-Switch II conformation, and suggests new testable hypotheses that some variants could differentially affect Ras and Rap. Enhanced GTP stabilization is proposed to explain the greater potency of SynGAP toward Rap1b. Overall, 33 missense variants, including eight ClinVar and 18 cancer-associated variants, were predicted to potentially impair SynGAP–GTPase complex formation.

**Author summary:**

We study SynGAP, a protein essential for how brain cells communicate, learn, and develop. Changes in the *SYNGAP1* gene are known to cause a neurodevelopmental disorder causing intellectual disability, autism, and epilepsy. SynGAP also helps regulate cell-growth pathways, and its dysfunction can contribute to cancer. In this work, we use computer-based approaches, including molecular dynamics simulations, to investigate how SynGAP interacts with the signaling GTPases Ras and Rap1b, which control key cellular processes. We show that SynGAP engages these proteins through two distinct interaction modes and model both at atom-level resolution. We also examine disease-associated missense variants and predict how they may disrupt these interactions. Our results help explain previously reported experimental findings and highlight regions of SynGAP that are critical for its function. By improving our understanding of how SynGAP regulates Ras and Rap1b, this work provides insight into SynGAP-related disorders and cancer as well as suggesting testable hypotheses for future experimental studies.

## Highlights

• SynGAP uses canonical RasGAP and Plexin-like RapGAP mechanisms.
• Determinants of SynGAP–GTPase selectivity revealed using in silico methods.
• Somatic cancer and SynGAP-related disorder missense mutations show a substantial overlap.
• Missense variants can affect stability and catalysis in SynGAP–Ras/Rap complexes.
• Pre-organization of GTPase active site by SynGAP explains 100-fold Rap activity over Ras.

## Graphical abstract

**Figure:**
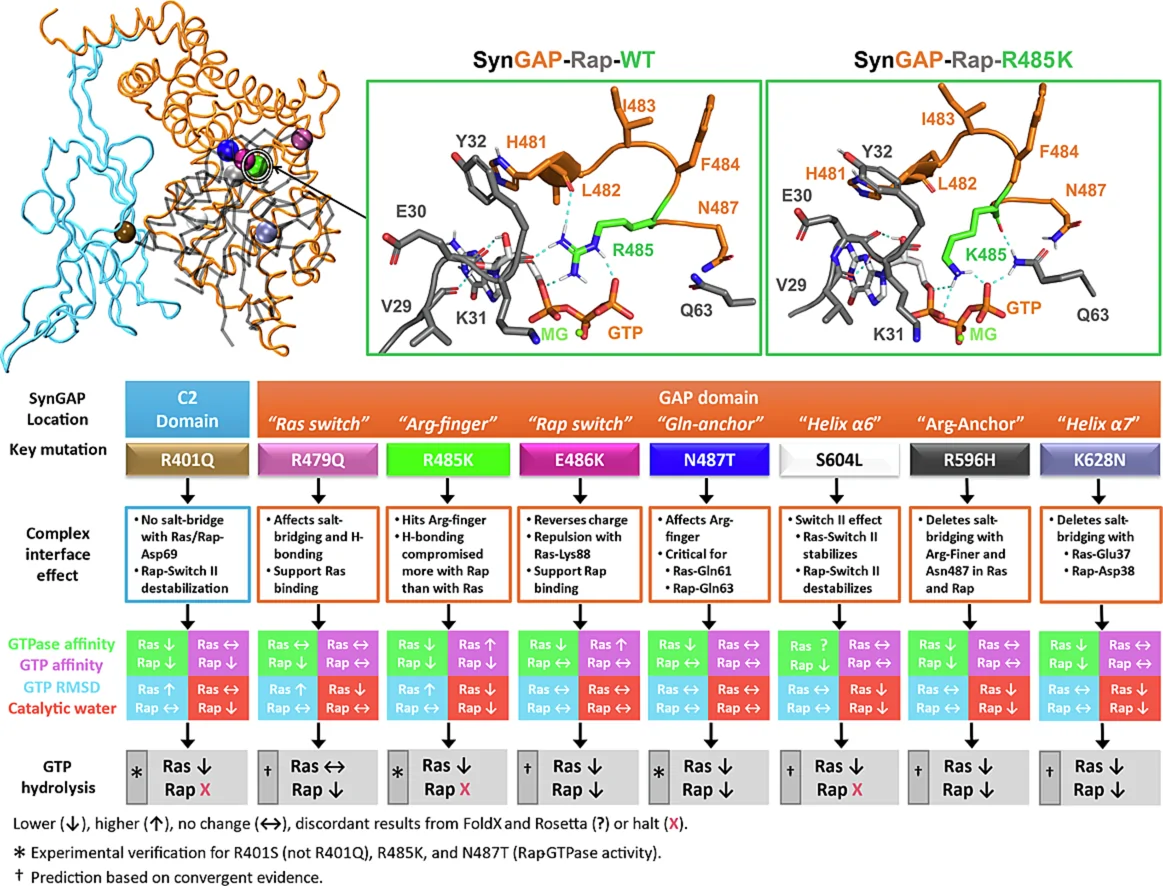

## Introduction

SynGAP or Synaptic GTPase Activating Protein (Fig. 1A), encoded by the *SYNGAP1* gene, is abundantly expressed at the postsynaptic density (PSD) of excitatory synapses in forebrain neurons (Araki et al., 2015). SynGAP deactivates Ras/Rap-GTPases by accelerating their intrinsic GTP hydrolysis through the stabilization of the GTPase Switch I and II regions into a catalytically competent conformation, while providing an essential “Arg-finger” into the active-site to stabilize the transition state of GTP (Figs. 1B, Fig. 2, Fig. 3A). Thereby, SynGAP is regulating signaling pathways involved in cell growth, differentiation, and cytoskeletal dynamics (Shah et al., 2018; Walkup 4th et al., 2018; Zhang et al., 2017; Gamache et al., 2020; Scheffzek et al., 1997), not only in the brain but also across other human tissues (e.g., thyroid gland, bronchus).

**Fig. 1:**
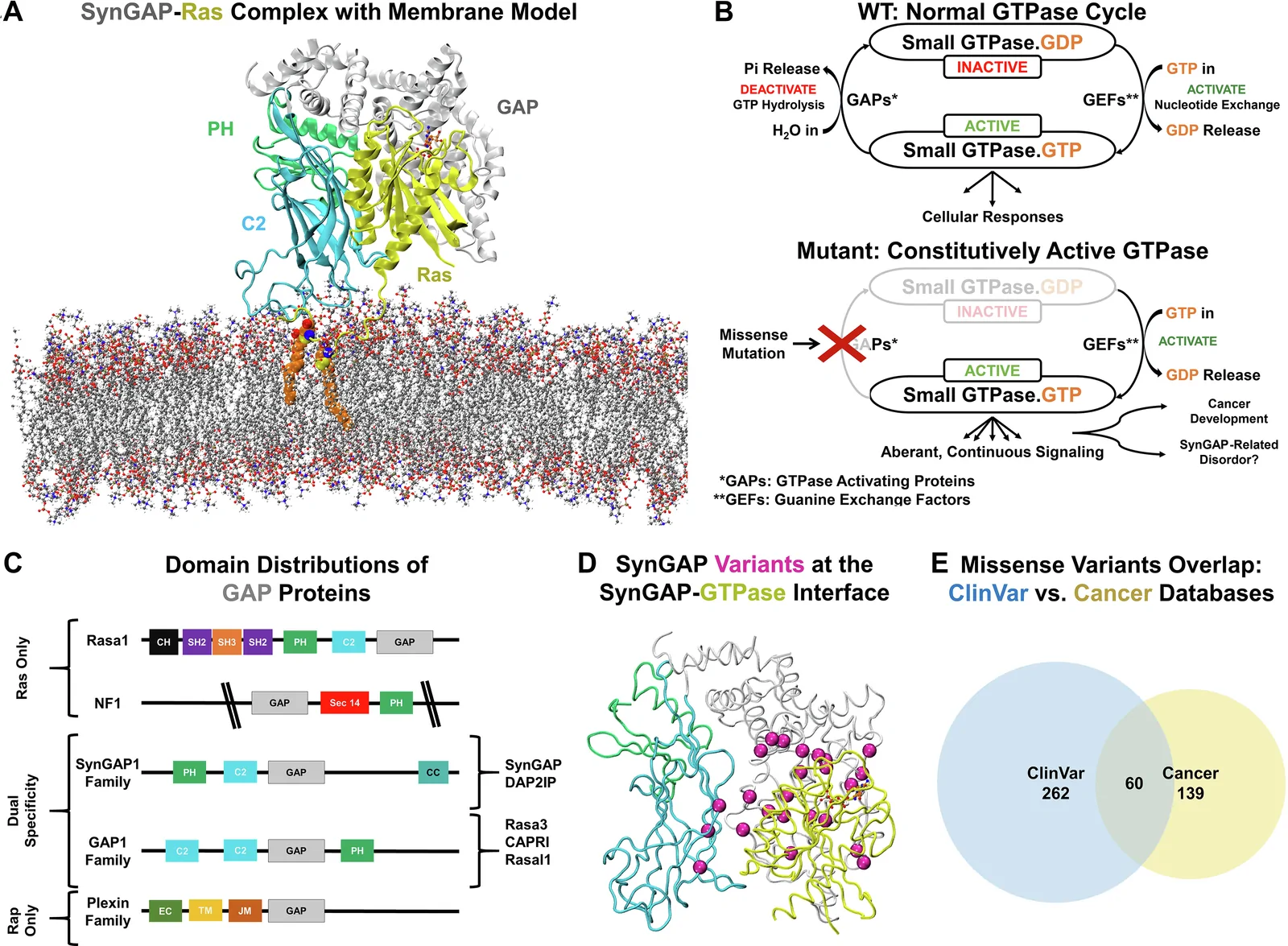
**SynGAP***–***Ras-GTPase complex, domain organization and interface missense mutations**. **(A)** The PH-C2-GAP (green; cyan; white) section of SynGAP is shown forming a complex with a GTP-bound Ras-GTPase (yellow) tethered at the inner synaptic membrane model via Cys181/184-palmitoyl lipids (orange CPK models) (Ali et al., 2024). Note that the modelled SynGAP*–*GTPase systems probed in this study exclude the lipid bilayer the same as the comparable in vitro studies on the topic (Pena et al., 2008; Paul et al., 2025a). **(B)** Normal cycle (top): GTPases toggle between inactive (GDP) and active (GTP) states via GEF-mediated exchange and GAP-stimulated hydrolysis. Mutant state (bottom): GAP function-blocking missense mutations result in constitutively active GTPases and aberrant signaling, potentially contributing to cancer development or even SRD symptoms. **(C)** Comparative schematic of domain architectures across representative GAP family members, highlights the conservation of the catalytic GAP domain alongside variable accessory domains and identifies the dual-specificity members. **(D)** SynGAP missense variants at the SynGAP*–*Ras interface (ribbon model), that are predicted to destabilize the complex formation by Rosetta and/or FoldX-MD approach, are shown as pink spheres. (E) Venn diagram of SynGAP variants within the N-terminal PH-C2-GAP segment found in ClinVar (*N* = 322) and cancer databases (*N* = 199) show a substantial overlap (*N* = 60). These represent all reported entries, regardless of whether they are known to be pathogenic. (For interpretation of the references to colour in this figure legend, the reader is referred to the web version of this article.)

**Fig. 2:**
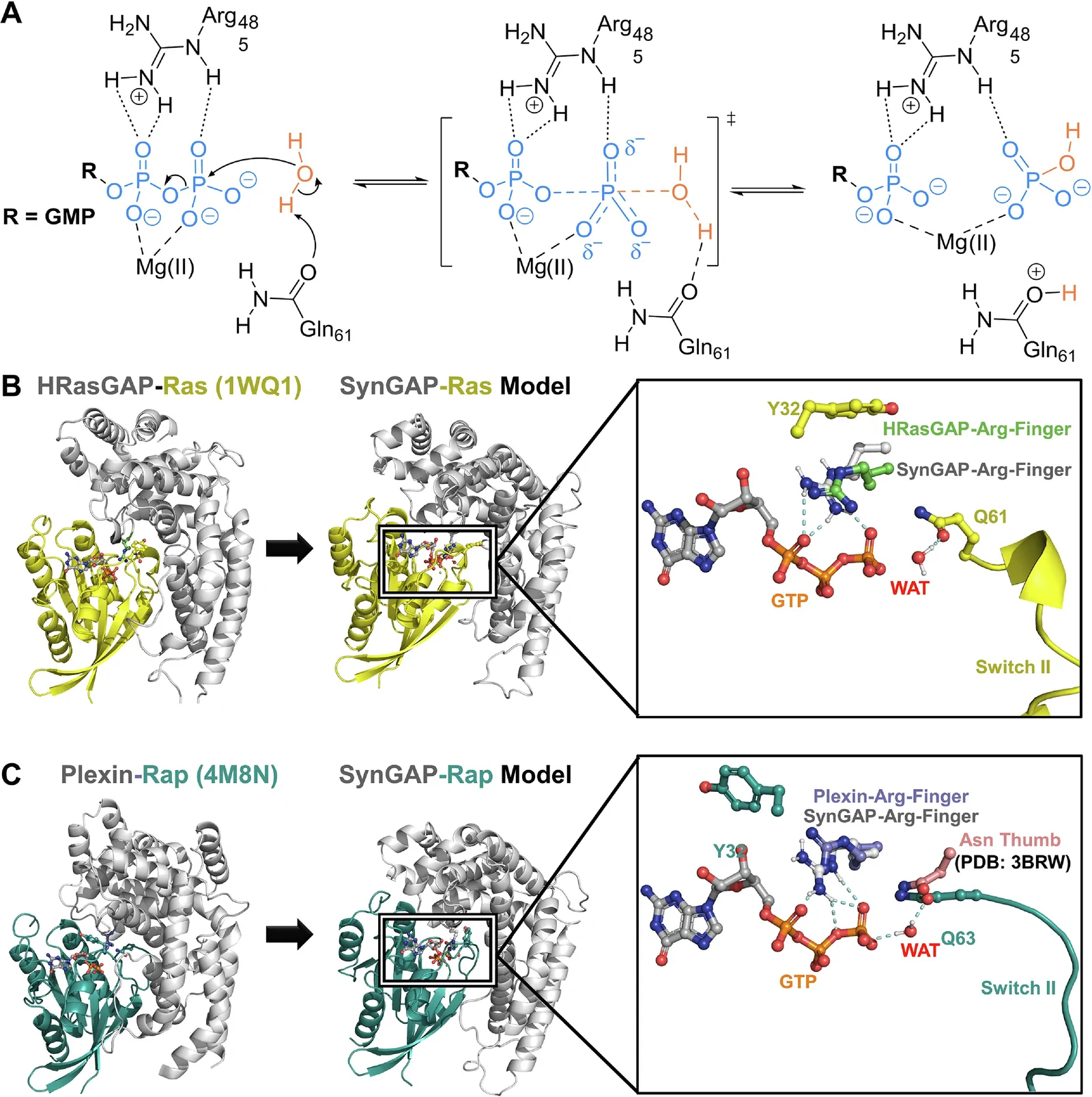
**SynGAP-induced Ras-and Rap-GTPase activation involves a conserved arginine finger based on structural modelling. (A)** S_N_2-type mechanism of GTP hydrolysis has Gln61 acting as the activating base and Arg-finger stabilizing the transition state, which in turn promotes γ-phosphate release. **(B)** The HRasGAP–Ras complex X-ray crystal structure (PDB: 1WQ1 (Scheffzek et al., 1997); left overview) was used as a template to model the SynGAP–Ras complex (middle) by protein backbone superposition. In the close-up view (right), the Arg-fingers of SynGAP (gray) and HRasGAP (green) align closely, positioning the SynGAP-Arg-finger into the active-site to stabilize the γ-phosphate of GTP. The catalytic arrangement is completed by Ras-Gln61 and a coordinated water molecule (WAT), both required to initiate nucleophilic attack during GTP hydrolysis. **(C)** Similarly, the SynGAP–Rap complex (middle overview) was modelled based on the Plexin–Rap X-ray crystal structure (PDB: 4M8N (Wang et al., 2013); left overview). In the close-up view, the Arg-fingers of SynGAP (gray) and Plexin (magenta) align well; in this configuration, Rap-Gln63 occupies a position equivalent to the Asn-thumb (pink sticks) observed in the RapGAP–Rap complex X-ray crystal structure (PDB: 3BRW (Scrima et al., 2008)). Protein 3D structures are shown as cartoons (Ras: yellow; Rap: teal; GAPs: white), while GTP (gray carbon atoms) and selected residues are depicted as ball-and-stick models. The catalytic water oxygen (red) is shown as a sphere, with hydrogens displayed only for the catalytic water and Arg-finger (white sticks). Key H-bonds are indicated by cyan dashed lines. (For interpretation of the references to colour in this figure legend, the reader is referred to the web version of this article.)

**Fig. 3:**
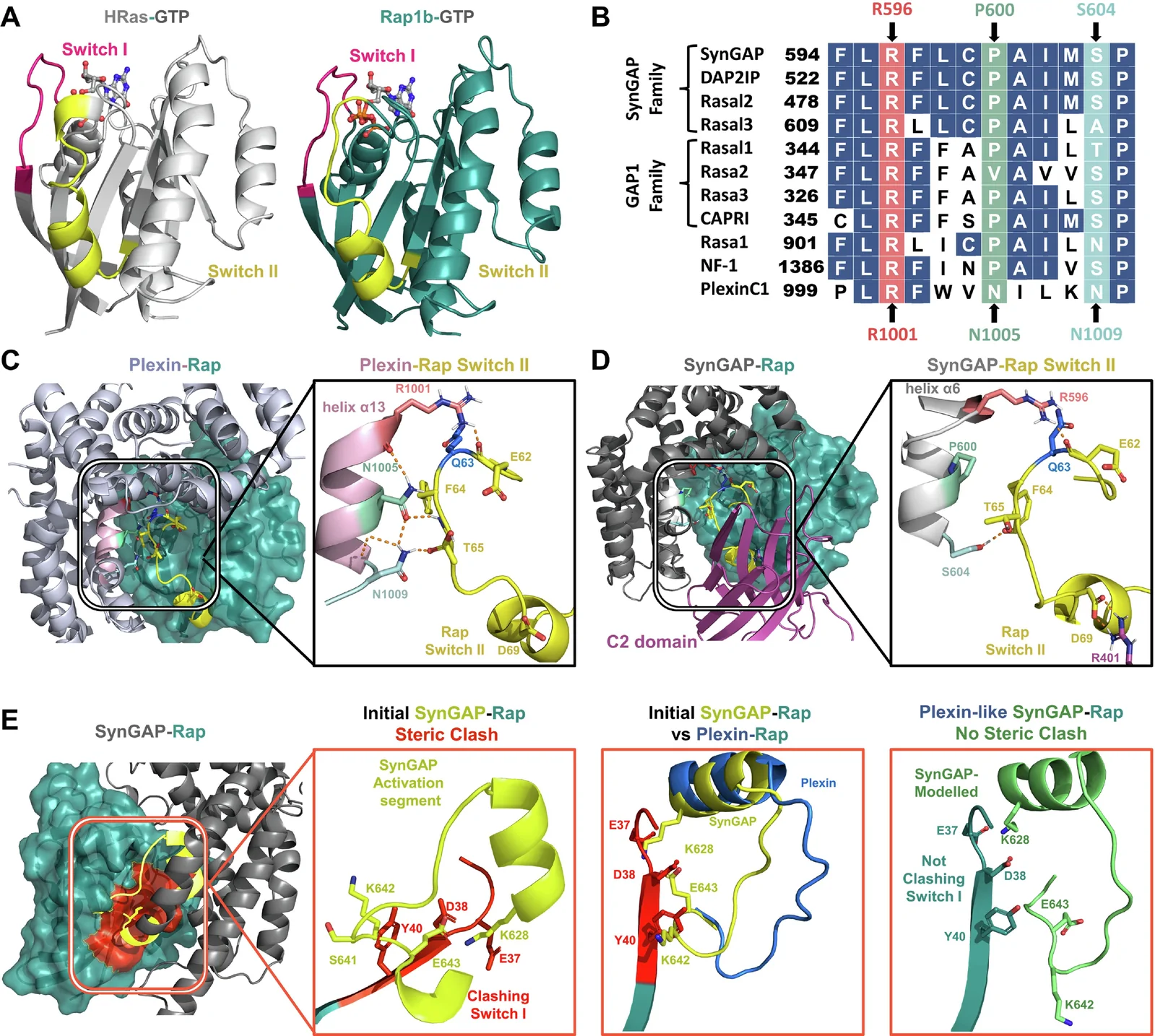
**Structural basis of SynGAP–Rap interface modelling and comparison to the Plexin–Rap complex. (A)** A cartoon model of Ras (white) and Rap (teal) bound to GTP (sticks with gray carbon) highlights the Switch I (magenta) and Switch II (yellow) regions. **(B)** Sequence alignment of the SynGAP α6 helix motif highlights residues corresponding to Plexin Arg1001 (pink), Asn1005 (green), and Asn1009 (cyan), showing that SynGAP contains Pro600 and Ser604 in place of the Plexin asparagines. **(C)** The Plexin–Rap complex shows coordinated positioning of Switch II (yellow), with the close-up view illustrating the H-bond network (orange dashes) between Plexin-helix α13 (light pink) and Rap residues (sticks). **(D)** In the final complex model, SynGAP adopts a Plexin-like arrangement that stabilizes the Rap surface (teal) through its GAP (gray) and C2 (magenta) domains; the inset shows Arg596 and Ser604 engaging the Rap-Switch II, while a salt-bridge forms between SynGAP-Arg401 and Rap-Asp69. **(E)** The initial complex model reveals a steric clash between the SynGAP-activation segment (yellow) and Rap-Switch I (right overview); the close-up views highlight the residues involved (left close-up), while comparison with Plexin (middle close-up; PDB: 4M8N) (Wang et al., 2013) shows that adopting an open loop conformation (right close-up) resolves the clash and enables a compatible binding geometry. (For interpretation of the references to colour in this figure legend, the reader is referred to the web version of this article.)

However, SynGAP (∼140 kDa) (Gamache et al., 2020) is substantially larger than its namesake GAP domain (∼21 kDa), featuring a conserved SynGAP-family domain architecture (Kupzig et al., 2006; Sot et al., 2010; Pena et al., 2008; Paul et al., 2025a) that includes N-terminal PH (Pleckstrin homology), C2 and GAP domains (Pena et al., 2008), and a C-terminal CC (coiled-coil) domain (Zeng et al., 2016) (Fig. 1C). This multidomain organization enables diverse functions beyond catalysis: the PH and C2 domains likely mediate membrane association (Fig. 1A) (Pena et al., 2008; Larsen and Sansom, 2021; Meili et al., 2021; Jeyabalan and Clement, 2016; Bernards, 2003), while the CC domain drives trimerization (Zeng et al., 2016). Furthermore, SynGAP, via its α1-isoform containing a C-terminal PDZ-binding motif, is pivotal in modulating synaptic plasticity by regulating AMPAR (α-amino-3-hydroxy-5-methyl-4-isoxazolepropionic acid receptor) trafficking at the PSD. By limiting the availability of PDZ-binding slots of PSD95 for AMPAR–TARP (Transmembrane AMPAR Regulatory Protein) complexes during long-term potentiation (LTP), SynGAP constrains excessive receptor recruitment, thereby supporting neuronal homeostasis and memory formation (Araki et al., 2015; Gamache et al., 2020; Pena et al., 2008; Meili et al., 2021; Jeyabalan and Clement, 2016; Araki et al., 1979).

Recent work by Araki et al. (Araki et al., 2024) indicates that this structural role of SynGAP, rather than its GAP function, would be primarily associated with SynGAP-related disorder (SRD) that is a spectrum of neurodevelopmental conditions of varying severity, including intellectual disability (ID), epilepsy, and autistic-like behaviors caused by de novo *SYNGAP1* mutations (Wright et al., 2022; Carvill et al., 2013; Araki et al., n.d.; Agarwal et al., 2019; Hamdan et al., 2009). Despite being a rare disease with an estimated incidence of 6 per 100,000, SRD is nevertheless one of the leading genetic causes of ID in children, accounting for ∼1% of all cases (López-Rivera et al., 2020; Graglia et al., 2025). While truncating variants are readily classified as pathogenic, missense mutations, which are likely behind most SRD cases, remain challenging to diagnose due to their subtle and often obscure structural effects (Ali et al., 2024). Homozygous deletion of the C2/GAP domains is lethal in rats, and, importantly, even if the GAP-disabling mutations of SynGAP are not the root cause of SRD, they have been reported to alter intrinsic neuronal excitability (Araki et al., 2024; Brill et al., 2025; Katsanevaki et al., 2024), and are, therefore, likely contributors to the SRD-related symptoms and disease phenotypes at least on some level. In a broader evolutionary context, SynGAP's GAP activity must contribute to normal physiology; otherwise, its catalytic function would likely have been lost, as observed for scaffolding protein IQGAP (Hedman et al., 2015) or in many pseudokinases (e.g., MAGUK-family (Won et al., 2017)).

In fact, GAP function and dual-Ras/Rap specificity are in many ways a defining feature of SynGAP (Fig. 1C). Ras-GTPases are central signaling regulators and, accordingly, ∼30% of cancers have oncogenic mutations conferring resistance to the RasGAP-mediated inactivation (Scheffzek et al., 1997; Bellazzo and Collavin, 2020; Shah et al., 2019). Likewise, Rap1 controls cell adhesion and tumor invasiveness (Zhang et al., 2017; Shah et al., 2019; Stefanini and Bergmeier, 2019), and aberrant RapGAP expression correlates with aggressive tumor phenotypes and poor clinical prognosis (Shah et al., 2018; Zhang et al., 2017; Stefanini and Bergmeier, 2019; Minato and Hattori, 2009). Together, Ras- and Rap-specific GAPs function as context-dependent tumor suppressors, where their loss or silencing promotes tumorigenesis in both the presence and absence of oncogenic Ras mutations (Bellazzo and Collavin, 2020; Shah et al., 2019; Stefanini and Bergmeier, 2019; Yeh et al., 2025; McLaughlin et al., 2013; Li et al., 2019; Grewal et al., 2011; De Florian et al., 2024). Indeed, SynGAP overexpression has been shown to inhibit rectal adenocarcinoma cell proliferation (Xiao et al., 2024) and suppress tumor invasion by blocking epithelial-mesenchymal transition (Li et al., 2019). SynGAP deactivates Rap ∼100-fold more potently than that of Ras (Pena et al., 2008). This differential activity is modulated by kinases that phosphorylate >20 sites, mainly within its C-terminal “disordered” segment (Shah et al., 2018; Zhang et al., 2017; Saito et al., 2015; Looi et al., 2020), and disfunction in this phosphoregulation is implicated in both neurodevelopmental pathology and cancer progression (Zhang et al., 2017; Scheffzek et al., 1997; Bellazzo and Collavin, 2020; McLaughlin et al., 2013; Li et al., 2019; Grewal et al., 2011; Saito et al., 2015; Looi et al., 2020; Zhou et al., 2019).

During GTP hydrolysis, RasGAPs use an Arg-finger to stabilize the departing γ-phosphate of GTP, while Ras-Gln61 orients the nucleophilic water (Fig. 2A–B). In contrast, Rap-GTPases rely on a catalytic “Asn-thumb” to coordinate the nucleophilic water (Fig. S1) (Sot et al., 2010; Hansmann et al., 2020; Scrima et al., 2008; Daumke et al., 2004). Dual-specificity GAPs (Kupzig et al., 2006; Pena et al., 2008; Qiao and Homayouni, 2015), including SynGAP (Fig. 1C), share the RasGAP-fold and Arg-finger but lack the RapGAP Asn-thumb and homology; thus, their non-canonical Rap activation mechanism has remained unclear. While no dual-specificity GAP–Rap complex has yet been solved experimentally, insights from the Plexin–Rap X-ray crystal structure (Wang et al., 2013) have suggested a “Plexin-like” non-canonical mechanism in which Rap-Gln63 is positioned for catalysis through stabilization of an open Switch II conformation (Figs. 2C and 3B–C vs. S1). Supporting this mechanism, Rap-Gln63 is required for the RapGAP-activity in dual-specificity GAPs, acting analogously to Ras-Gln61 (Sot et al., 2010; Kupzig et al., 2009). This open conformation is stabilized by a Plexin-specific H-bonding network, that is absent in dual-specificity GAPs such as SynGAP (Wang et al., 2013). Instead, SynGAP is thought to rely on its C2 domain in the Switch II stabilization (Fig. 3A–D), consistent with the C2 domain being indispensable for the Rap deactivation by both SynGAP- and GAP1-family members (Sot et al., 2010; Pena et al., 2008; Paul et al., 2025a; Kupzig et al., 2009).

In this study, atomistic molecular dynamics (MD) simulations, in silico mutagenesis, and binding free-energy calculations were applied to elucidate (1) the molecular mechanisms underlying the dual-Ras/Rap-GTPase activation by SynGAP (Fig. 2B–C), and (2) how somatic cancer and SRD-linked missense mutations modulate SynGAP–Ras/Rap association (Fig. 1D–E). As suggested in prior work (Ali et al., 2024), Ras-GTPase deactivation by SynGAP is likely to follow the canonical RasGAP-mechanism involving the Arg-finger and Ras-Gln61 (Fig. 2B). Conversely, Rap regulation is suggested to follow a Plexin-like mechanism in which SynGAP stabilizes an extended Rap-Switch II conformation, positioning Rap-Gln63 analogously to Ras-Gln61 through coordinated interactions involving the C2 (Arg401) and GAP (Arg596, Ser604) domains (Figs. 2C and 3D). Among the 214 analyzed missense variants (Table S1), mainly reported in the cancer databases (Gao et al., 2013; Sondka et al., 2024; Sayers et al., 2022), 33 were predicted to disrupt complex stability and/or GAP-mediated GTP hydrolysis geometry, including eight ClinVar-listed variants potentially linked to SRD (Fig. 1E; Tables 1 and S2).

**Table 1:** Binding affinities and folding stabilities for SynGAP–Ras/Rap GTPase complexes.

**Variant**	**SynGAP–Ras Complex**			**SynGAP–Rap Complex**		
	**FoldX-MD**$\;{\mathrm{\Delta \Delta G}}_{\mathrm{bind}}$^1^	**Rosetta**$\;{\mathrm{\Delta \Delta G}}_{\mathrm{bind}}$^2^	**Foldx-MD**$\;{\mathrm{\Delta \Delta G}}_{\mathrm{fold}}$^3^	**FoldX-MD**$\;{\mathrm{\Delta \Delta G}}_{\mathrm{bind}}$^1^	**Rosetta**$\;{\mathrm{\Delta \Delta G}}_{\mathrm{bind}}$^2^	**Foldx-MD**$\;{\mathrm{\Delta \Delta G}}_{\mathrm{fold}}$^3^
R272W	**0.7 ± 0.4**⁎⁎⁎	**1 ± 0.3**⁎⁎⁎	**4.0 ± 0.3**	**0.7 ± 0.1**⁎⁎⁎	**0.6 ± 0.3**⁎⁎⁎	**3.2 ± 0.1**
E360K	**0.6 ± 0.2**⁎⁎⁎	-0.4 ± 1⁎	2.0 ± 0.2	**1.0 ± 0.3**⁎⁎⁎	0.3 ± 0.3	2.6 ± 0.3
R401Q†	**1.6 ± 0.9**⁎⁎⁎	0.4 ± 0.5	**3.0 ± 0.8**	**3.9 ± 0.6**⁎⁎⁎	**1.3 ± 0.4**⁎⁎⁎	**4.9 ± 0.9**
R401W	**1.1 ± 0.7**⁎⁎⁎	-0.1 ± 0.4	**3.3 ± 0.9**	**3.0 ± 0.6**⁎⁎⁎	**0.6 ± 0.3**⁎⁎⁎	**4.6 ± 1.1**
N440I	0 ± 0	**-0.5 ± 0.1**⁎⁎⁎	1.0 ± 0.1	**0.5 ± 0.3**⁎⁎	0 ± 0.1	1.6 ± 0.1
K442R	**-0.6 ± 0.1**⁎⁎⁎	0 ± 0.1	-0.4 ± 0.1	**-1.1 ± 0**⁎⁎⁎	**0.5 ± 0.3**⁎⁎	-0.9 ± 0.1
R479Q†	0.3 ± 0.2	0 ± 0.1	2.1 ± 0.5	**1.8 ± 0.2**⁎⁎⁎	0.2 ± 0.2	2.2 ± 0.1
R479W	0 ± 0.2	-0.1 ± 0.1	1.4 ± 0.5	**1.5 ± 0.1**⁎⁎⁎	0.1 ± 0.1	1.7 ± 0.1
H481D	**1.1 ± 0.1**⁎⁎⁎	**0.6 ± 0.2**⁎⁎⁎	1.1 ± 0.1	**0.9 ± 0.2**⁎⁎⁎	**0.7 ± 0.7**⁎⁎	1.2 ± 0.0
F484A	0.1 ± 0	0 ± 0	**4.3 ± 0.1**	0 ± 0	0 ± 0.1	**4.5 ± 0.2**
R485L-F484A	-0.1 ± 0.1	**-0.6 ± 0.3**⁎⁎⁎	**5.9 ± 0.2**	-0.1 ± 0.1	-0.1 ± 0.5	**6.4 ± 0.3**
R485L	-0.1 ± 0.1	**-0.7 ± 0.2**⁎⁎⁎	1.5 ± 0.1	-0.2 ± 0.1	-0.1 ± 0.5	1.8 ± 0.1
R485K	**0.7 ± 0.2**⁎⁎⁎	**0.8 ± 0.2**⁎⁎⁎	2.0 ± 0.3	**0.5 ± 0.0**⁎⁎⁎	**0.7 ± 0.4**⁎⁎⁎	2.0 ± 0.1
R485H	**2.0 ± 0.9**⁎⁎⁎	0.2 ± 0.5	**4.5 ± 0.9**	**0.7 ± 0.1**⁎⁎⁎	0.2 ± 0.4	**3.2 ± 0.3**
R485P	**2.1 ± 0.3**⁎⁎⁎	**1.1 ± 0.9**⁎⁎⁎	**7.9 ± 0.6**	**1.0 ± 0.2**⁎⁎⁎	**0.8 ± 0.4**⁎⁎⁎	**7.1 ± 0.9**
E486K†	**0.9 ± 0.2**⁎⁎⁎	0.3 ± 0.2	1.6 ± 0.6	0.3 ± 0.2	0 ± 0.3	0.5 ± 0.2
N487K	**0.8 ± 0.7**⁎⁎	0.1 ± 0.6	2.2 ± 0.7	**0.5 ± 0.2**⁎⁎⁎	**0.6 ± 1.4**⁎	2.0 ± 0.5
N487T	**1.1 ± 0.5**⁎⁎⁎	-0.3 ± 0.2	**2.7 ± 0.5**	0.4 ± 0.2	-0.1 ± 0.1	2.5 ± 0.1
K492E†	**1.7 ± 0.4**⁎⁎⁎	**1 ± 0.2**⁎⁎⁎	**4.1 ± 0.4**	**1.4 ± 0.6**⁎⁎⁎	**0.5 ± 0.3**⁎⁎⁎	**3.4 ± 0.4**
R596H†	**1.3 ± 0.4**⁎⁎⁎	-0.1 ± 0.3	**4.9 ± 0.5**	**1.2 ± 0.1**⁎⁎⁎	**0.5 ± 0.3**⁎⁎	**4.8 ± 0.6**
P600V	**1.0 ± 0.4**⁎⁎⁎	-0.2 ± 0.2	**3.5 ± 0.4**	**0.7 ± 0.4**⁎⁎⁎	0.2 ± 0.3	**3.1 ± 0.7**
M603I	**1.1 ± 0.3**⁎⁎⁎	**0.7 ± 0.3**⁎⁎⁎	1.4 ± 0.4	**0.7 ± 0.2**⁎⁎⁎	0.3 ± 0.2	1.2 ± 0.3
S604L†	**1.4 ± 1.6**⁎	**-0.5 ± 0.2**⁎⁎⁎	1.1 ± 1.2	**1.4 ± 0.7**⁎⁎⁎	**1.6 ± 0.9**⁎⁎⁎	1.0 ± 0.7
L607F	0.3 ± 0.5	0.3 ± 0.3	1.0 ± 0.7	0.4 ± 0.2	**0.7 ± 0.3**⁎⁎⁎	1.0 ± 0.3
Y614C	0.4 ± 0.2	**0.5 ± 0.3**⁎⁎⁎	2.0 ± 0.5	**0.6 ± 0.5**⁎⁎⁎	0.4 ± 0.3	2.2 ± 0.2
R621Q†	**1.5 ± 0.4**⁎⁎⁎	0.2 ± 0.4	2.7 ± 0.2	**1.6 ± 0.9**⁎⁎⁎	0.2 ± 0.3	2.2 ± 0.2
A627V	0.2 ± 0	0.4 ± 0.2	2.4 ± 0.6	0.1 ± 0.1	**0.6 ± 0.7**⁎⁎	1.9 ± 0.6
K628R	**-1.1 ± 0**⁎⁎⁎	**0.8 ± 0.2**⁎⁎⁎	-1.1 ± 0.2	**-1.0 ± 0.2**⁎⁎⁎	0.1 ± 0.4	-1.3 ± 0.2
K628N	**1.9 ± 0.4**⁎⁎⁎	**1.1 ± 0.3**⁎⁎⁎	2.7 ± 0.3	**1.1 ± 0.3**⁎⁎⁎	0.4 ± 0.6	1.8 ± 0.2
N635S†	**1.2 ± 0.3**⁎⁎⁎	0.4 ± 0.1	1.4 ± 0.3	**0.6 ± 0.1**⁎⁎⁎	**0.5 ± 0.5**⁎⁎⁎	1.9 ± 0.4
E634Q	**-1.1 ± 0.6**⁎⁎⁎	-0.3 ± 0.2	-0.5 ± 0.4	-0.2 ± 0.1	0 ± 0.4	0.6 ± 0.1
D644N	0 ± 0	**0.7 ± 0.6**⁎⁎⁎	-0.1 ± 0.1	0.4 ± 0.2	0.3 ± 0.1	0.5 ± 0.1
D644A	0.1 ± 0.1	**0.6 ± 0.6**⁎⁎⁎	0 ± 0	**0.7 ± 0.3**⁎⁎⁎	0.2 ± 0.3	1.0 ± 0.3

Binding affinities and folding stabilities for the WT and missense SynGAP–Ras and SynGAP–Rap complexes, where positive ΔΔG values indicate destabilization and negative values indicate stabilization, with all ΔG and ΔΔG values reported in kcal/mol. Standard deviation (SD), reported as ± values, represents between-replica variability. It was calculated as the sample SD of the mean ΔΔG values from the three WT replica ensembles for FoldX-MD, and as the sample SD of the ΔΔG values obtained from three independently selected WT templates for Rosetta Flex ddG.

Variants included in this table are those that exceeded the energetic thresholds in either Rosetta or FoldX. Numerical values are shown in bold for mildly destabilizing or stabilizing effects (${\mathrm{\Delta \Delta G}}_{\mathrm{bind}}$ ≥ 0.5 or ≤ −0.5 kcal/mol), and bolded/underlined for significant perturbations (${\mathrm{\Delta \Delta G}}_{\mathrm{bind}}$ ≥ 1.0 or ≤ −1.0 kcal/mol, or $\;{\mathrm{\Delta \Delta G}}_{\mathrm{fold}}$ ≥ 3 or ≤ −3 kcal/mol).

1 FoldX-MD $\;{\mathrm{\Delta \Delta G}}_{\mathrm{bind}}$ values were computed using *AnalyseComplex* on a combined set of 380 WT simulation frames per replica, comprising the original 180 frames from the 200-ns simulations (first 20 ns discarded; one frame per ns thereafter) together with an additional 200 frames from the restarted and extended WT simulations. The three WT replica datasets were processed independently, and variant ΔΔG values represent the mean and SD across the three combined WT reference ensembles.

2 Rosetta ${\mathrm{\Delta \Delta G}}_{\mathrm{bind}}$ values were calculated using three WT template structures, each selected as the lowest-energy frame based on the MM/GBSA analysis of its corresponding replica simulation and used as input for FlexddG. Reported values are the mean and SD across the three templates.

3 FoldX-MD $\;{\mathrm{\Delta \Delta G}}_{\mathrm{fold}}$ folding stability values were computed using *BuildModel* with 380 WT simulation frames the same way as explained above for FoldX-MD (See 1).

⁎⁎⁎ Strong/reproducible effect: all three replicas show the same direction and ≥ 2/3 exceed the predefined energetic threshold.

⁎⁎ Moderate effect: all three replicas show the same direction, but the threshold is not consistently exceeded.

⁎ Uncertain effect: replicas disagree in the direction of the energetic change. See Table S2 for individual replica values.

† Missense variants correspond to ClinVar-annotated substitutions, classified as VUS (R401Q, E486K, K492E, S604L, N635S) or as likely benign (R479Q, R596H, R621Q).

In addition to analyzing disease-linked missense mutation (Fig. 1D) effects on RasGAP-activity of SynGAP, this study establishes the first atomistic model of the SynGAP–Rap1b complex (Fig. 2C), providing mechanistic insight into its non-canonical Rap-activation mechanism and mutation consequences.

## Methods

### System preparation

#### WT-SynGAP–Ras complex system preparation

The WT (wild-type) SynGAP–Ras complex (Fig. 1A) was modelled as described previously (Ali et al., 2024). The ≥50% reliable and continuous PH-C2-GAP segment SynGAP-α2 isoform (UniProt: SYGP1_HUMAN; Q96PV0) structure (residues 198–730, AF-Q96PV0-F1-model_v3.pdb) was acquired from the AlphaFold database (Jumper et al., 2021) (https://alphafold.ebi.ac.uk/; accessed 11/22/2022). Thus far, the AlphaFold3-SynGAP models have shown no substantial differences within the PH-C2-GAP segment compared to the applied AlphaFold2 model (data not shown). The Ras positioning was modelled by aligning the model's GAP domain against the GAP domain of human GAP-Ras complex (PDB: 1WQ1 (Scheffzek et al., 1997); Fig. 2B). The GDP-AlF3 pseudo-substrate was converted to GTP and the catalytic water (HOH 230) was retained. This WT-SynGAP–Ras complex model, where the Arg-finger directly interacts with the γ-phosphate and Gln61 adopts catalytic orientation engaging the catalytic water molecule (WAT in Fig. 4A–B), served as the template for the MD simulation and subsequent in silico mutagenesis steps (Fig. S2).

**Fig. 4:**
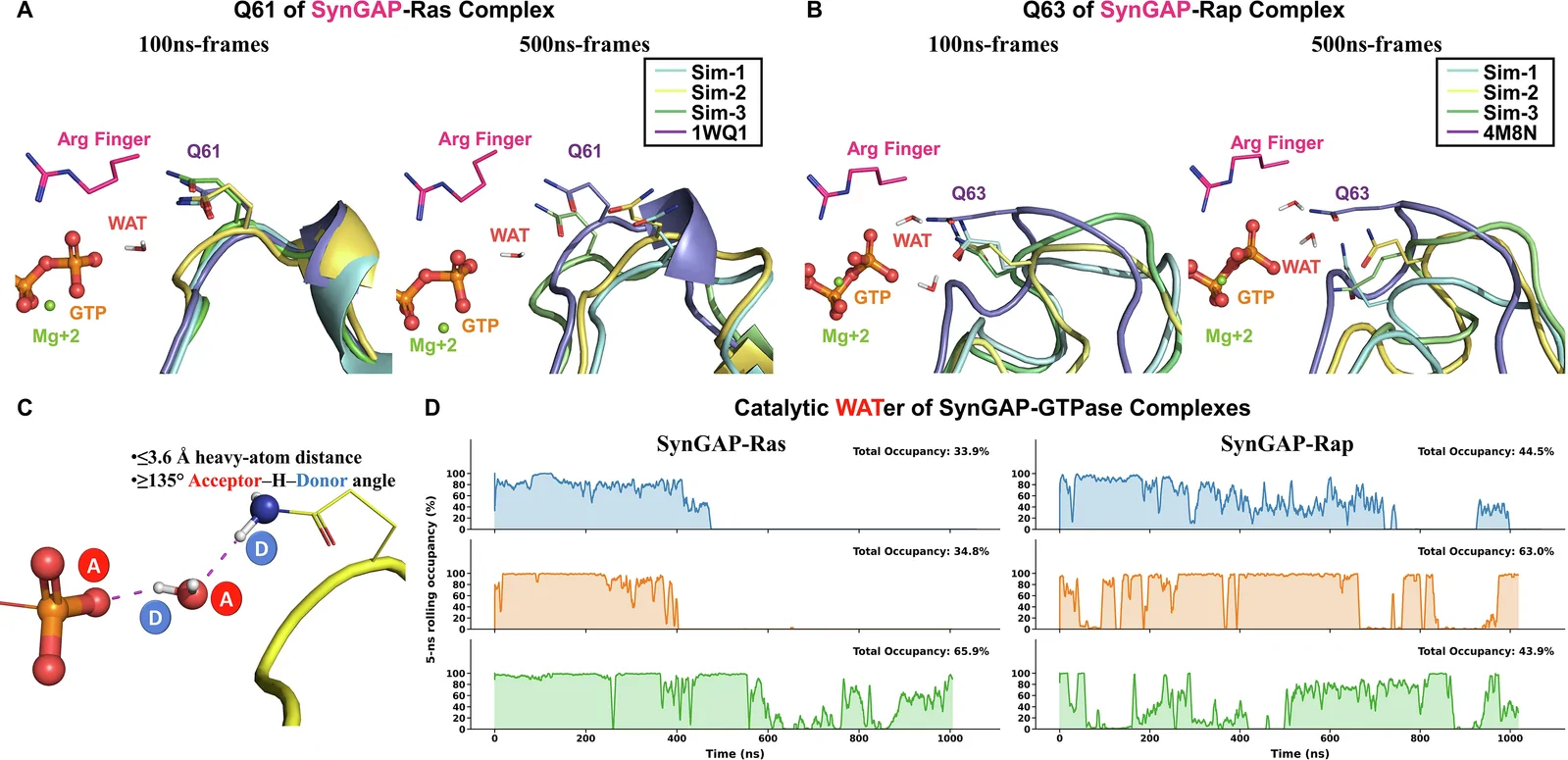
**Stability of the catalytic environment and water coordination in the SynGAP–Ras/Rap complex simulations. (A)** Structural overlays of the catalytic active-site in the SynGAP–Ras complex at 100 ns, shows the simulated conformations of the catalytic glutamine (Ras-Q61) across three independent replicas remain tightly clustered around the reference X-ray crystal structure (PDB: 1WQ1 (Scheffzek et al., 1997); purple). However, by 500 ns, Gln61 exhibits conformational drift and structural divergence. **(B)** In contrast, in the SynGAP*–*Rap complex, the catalytic glutamine (Rap-Gln63) displays more structural flexibility and deviation from the reference (PDB: 4M8N (Wang et al., 2013); purple) increasing over time at both 100 ns and 500 ns. **(C)** Geometric criteria utilized to define functional water-bridge involving the catalytic water positioned between the side-chain amide nitrogen (Nε2) of glutamine (Donor, D) and the GTP γ-phosphate oxygen atoms (Acceptor, A), requires a heavy-atom distance of ≤3.6 Å and an Acceptor-H-Donor angle of ≥135°. **(D)** The plots show the 5-ns rolling occupancy (%) across three independent 1-μs replicas (Blue: Sim-1, Orange: Sim-2, Green: Sim-3), measuring how consistently the complex maintains functional water-bridge at the active-site. In the SynGAP–Ras complex, the catalytic water maintains high initial stability for the first 400 to 500 ns before dropping sharply as Gln61 diverges, resulting in overall 1-μs total occupancies of 33.9% (Sim-1), 34.8% (Sim-2), and 65.9% (Sim-3). In contrast, SynGAP–Rap displays a highly dynamic water network characterized by frequent transient drops and recoveries throughout the 1 μs simulation time, yielding total occupancies of 44.5% (Sim-1), 63.0% (Sim-2), and 43.9% (Sim-3). Annotated values represent the cumulative total occupancy over the full 1-μs run. (For interpretation of the references to colour in this figure legend, the reader is referred to the web version of this article.)

#### WT-SynGAP–Rap1b complex system preparation

Initially, WT-SynGAP–Rap1b complex was modelled based on the RapGAP–Rap1b complex X-ray crystal structure (PDB: 3BRW (Scrima et al., 2008)). It required repositioning of Rap-Tyr32 to resolve steric clash with the Arg-finger in MAESTRO (Schrödinger Release 2022-3: Maestro, Schrödinger); yet, 250-ns unbiased MD simulations failed to achieve an open conformation for Tyr32 that allows direct interaction of the Arg-finger with GTP or satisfactory positioning of catalytic Gln63 within the active-site. (Fig. S1). Consequently, the SynGAP–Rap1b complex was modelled again by mimicking the PlexinC1–Rap1b complex X-ray crystal structure (PDB: 4M8N (Wang et al., 2013); Fig. 2C). In canonical RapGAP–Rap complex, Rap1b-Switch II is α-helical, positioning Gln63 away from the active-site, while in PlexinC1–Rap1b complex, Rap-Switch II adopts a tight hairpin loop conformation that positions Gln63 adjacent to GTP, analogous to Ras-Gln61. Besides, Tyr32 adopts an open state, eliminating the steric clash with SynGAP-Arg485 (Fig. 2C and S1) observed in the initial RapGAP-based model, and providing favorable structural features for subsequent modelling work.

In the final SynGAP–Rap1b complex model (Fig. 2C), Arg485, Gln63, catalytic water, and “activation segment” (residues 628–645) were positioned carefully to assure a catalytically competent geometry and unobstructed assembly:

• **Superposition and active-site alignment**: AlphaFold2-SynGAP model residues 198–730 were aligned onto the Plexin-GAP domain (PDB: 4M8N (Wang et al., 2013); Chain D: residues 637–754 and 935–1141) using PyMOL 2.6.0 (Schrodinger, 2019) (Fig. 2C). This positioned SynGAP's Arg-finger (Arg485) analogously to the Plexin-Arg711, enabling contact with the γ-phosphate of GTP (see Section 0).
• **Placement of catalytic water at the active-site:** The catalytic water was retained from human GAP–Ras X-ray crystal structure (PDB: 1WQ1 (Scheffzek et al., 1997); HOH230) by aligning Ras-Gln61 with Rap-Gln63 using PyMOL, preserving its position relative to the catalytic glutamine (WAT in Fig. 2C).
• **Activation segment modelling:** The Plexin activation segment (residues 1038–1058) was modelled onto the corresponding SynGAP region (residues 628–645) using MODELLER 10.4 (Šali and Blundell, 1993) to adopt the “open” conformation (Fig. 3E). The native “closed” Plexin conformation caused steric clashes with Rap1b (e.g., Pro1050-Tyr40, Asn1052-Asp38), and the SynGAP model showed analogous clashes. To resolve these clashes, SynGAP residues 635–645 were modelled to match the “open” conformation of PlexinC1 (residues 1038–1051).

To make the modelling match with the SynGAP–GTPase in vitro experiments (e.g., (Pena et al., 2008; Paul et al., 2025a)) and to keep the system size manageable, the explicit membrane (Fig. 1A) nor C-terminal residues with covalently linked lipid anchors were not included.

#### In silico mutagenesis: SynGAP missense variant system preparation

A comprehensive dataset of somatic cancer SynGAP missense mutations within the N-terminal PH-C2-GAP model (*N* = 199) were extracted from the cBioPortal (https://www.cbioportal.org/) (Gao et al., 2013), COSMIC (https://cancer.sanger.ac.uk/cosmic) (Sondka et al., 2024), and NIH Genomic Data Commons (https://gdc.cancer.gov/) (Heath et al., 2021) databases, accessed on 02/28/2025. This variant set was supplemented with 15 additional missense mutations positioned at the SynGAP–GTPase interface, selected based on their structural proximity (gnomAD: E360K, K442R, K628R, K628N; ClinVar: K492E, W362R; other: H481D, D644A) or prior literature relevance (engineered: F484A, R485P, R485K, F484A/R485L, R485L, N487T, P600V) (Scheffzek et al., 1997; Pena et al., 2008; Araki et al., 2024; Wang et al., 2013). The individual effects of the F485A and R485L substitutions, which comprise the F484A/R485L double variant, have not yet been experimentally investigated. In total, 60 of the cancer-linked variants in the N-terminal PH–C2–GAP model are also listed in ClinVar and may therefore be associated with SRD (Figs. 1E and S2).

### Molecular dynamics simulations

#### Simulation settings and parameters

The WT and missense variant SynGAP–Ras and SynGAP–Rap1b complexes were solvated in explicit water using a periodic box extending at least 15 Å from any solute atom to the box boundary (∼141,900 and ∼ 139,200 water molecules, respectively), thereby ensuring a minimum 15 Å separation between periodic images. Systems were neutralized with Na^+^ counterions using TLEaP in AmberTools22 (Aktulga et al., 2022). Proteins and solvent were parameterized with the ff19SB and OPC (Optimal Point-Charge) force fields, respectively (Tian et al., 2020; Izadi et al., 2014). The default OPC compatible 12-6 Li/Merz ion parameters were applied for Mg^2+^ ion (Li et al., 2015). The energy minimization was performed in two stages (side-chains/solvent only, then all atoms; 2000 steps each) using steepest descent followed by conjugate gradient. Equilibration proceeded in four 250-ps stages with decreasing positional restraints (4 → 1 kcal.mol^-1^.Å^-2^). Heating was performed under canonical ensemble (NVT), followed by isothermal-isobaric ensemble (NPT) equilibration. The temperature (310*K*) and the pressure (1 bar) were maintained with Langevin dynamics and isotropic Berendsen barostat, respectively (Loncharich et al., 1992; Berendsen et al., 1984). All covalent bonds involving hydrogen atoms were constrained using SHAKE (Miyamoto and Kollman, 1992). The AMBER input files are available in the Supporting Information of our prior study (Ali et al., 2024).

#### WT-SynGAP–GTPase simulations

The SynGAP–Ras (∼11,080 atoms) and SynGAP–Rap complex (∼11,140 atoms) model systems were equilibrated via the MD protocol described above (Ali et al., 2024) (3 × 200 ns) with AMBER22 (Case et al., 2023a). MM/GBSA (Molecular Mechanics with Generalized Born and Surface Area Solvation) binding free energies were calculated using the trajectories (excluding the first 20 ns; see Section below) to identify the best equilibrated frames. The lowest complex energy frames were selected as reference structures for the subsequent in silico mutagenesis protocol (Fig. S2; SynGAP–Rap: 150.90 ns, ΔG = −128.99 kcal/mol; SynGAP–Ras: 91.96 ns, ΔG = −123.76 kcal/mol).

To validate the structural integrity of modelled complexes prior to mutagenesis, we analyzed Ras-Switch I (residues 30–38) and II (60–76; Fig. 3A) stabilizing interactions across three 200-ns WT trajectories. Interface stability, involving the Arg-finger loop (480–488), helices α6 (596–606) and α7 (621–635), and the C2 domain, was assessed visually in VMD 1.9.4a12 (Humphrey et al., 1996), and for electrostatic contact changes using CPPTRAJ in AMBERTOOLS22 (Aktulga et al., 2022). For the SynGAP–Rap1b system, the PlexinC1–Rap1b interface (Fig. 3B–E) served as the structural benchmark to ensure the model maintained a catalytically relevant extended Switch II conformation throughout equilibration.

This validation step confirmed that the equilibrated low-energy complexes retained catalytically relevant geometries, establishing their suitability as templates or starting points for subsequent in silico mutagenesis and variant simulations. However, when extending the WT simulations to 1 μs, the Ras/Rap active-site coordination, especially regarding the Gln61/Gln63 side-chain placement or their water bridging to GTP, became progressively labile at ∼400–500 ns (Fig. 4A-D). Hence, the most detailed simulation analysis in this study was restricted to the initial 500 ns featuring the most biologically relevant active-site coordination in the WT simulations.

#### SynGAP missense variant–Ras/Rap-GTPase simulations

In total, 27 SynGAP missense variants (Table 1 and S2) were modelled using MODELLER 10.4 (Šali and Blundell, 1993) using the lowest energy WT reference frames as templates (Section 0). The in silico mutagenesis was restricted to the substituted residue to ensure direct comparability with the WT-SynGAP–Ras/Rap complexes. All variants were probed using MD simulations at least 100 ns; however, to match the longer WT system analysis, the missense variant simulations were run for 500 ns for 14 missense variants flagged destabilized specifically by the FoldX-MD and/or Rosetta interaction energy calculations (Section 0.). Finally, MM/GBSA binding free energies were also computed for all production MD trajectories (Section 0.; Fig. S2).

### Interaction energy and folding stability analysis

The interaction energy analysis for missense variants focused on protein-protein interaction components, excluding GTP, to enable consistent comparison across a large number of variant systems. GTP binding was evaluated separately using MM/GBSA calculations to capture variant-induced effects within the catalytic pocket. For FoldX-MD and Rosetta interface-energy calculations, variants were classified based on the mean energetic effect across simulation replicas (Table 1). To assess reproducibility, variants exceeding the predefined energetic threshold in either method (ΔΔG > 0.5 kcal/mol or < −0.5 kcal/mol) were assigned one of three confidence categories. Variants were classified as strong/reproducible (***) when all three replicas showed the same direction of energetic change and at least two exceeded the predefined threshold, moderate (**) when all three replicas showed the same direction of change but the threshold was not consistently exceeded, and uncertain (*) when the replicas disagreed on the direction of the energetic change. Individual replica values are provided in Table S2. MM/GBSA calculations were used primarily as a complementary method for evaluating protein complex stability and GTP binding, whereas variant effect interpretation relied principally on FoldX-MD and Rosetta predictions.

#### MM/GBSA calculations

Binding free energies of SynGAP–GTPase complexes were calculated using MM/GBSA method (mmpbsa.py) in AMBER22 (Case et al., 2023a). For protein–protein affinity, SynGAP was treated as the receptor and Ras or Rap1b as the ligand. Topology files were prepared with ante-MMPBSA.py, excluding water, counter ions, Mg^2+^, and GTP (Fig. S3). While GTP affinity was calculated using the SynGAP–GTPase complex as receptor and GTP as ligand, excluding water, counter ions, while retaining Mg^2+^ (Fig. S4). All calculations utilized the mbondi2 radii, igb = 2 (Onufriev GB model) and a 0.150 M salt concentration (Case et al., 2023a; Homeyer and Gohlke, 2012). The MM/PBSA (Molecular Mechanics with Poisson-Boltzmann and Surface Area Solvation) method produced highly similar results to MM/GBSA (data not shown).

#### Hybrid FoldX-MD and Rosetta FlexddG strategy

To evaluate the impact of each SynGAP missense mutation on complex formation, a hybrid screening strategy, integrating the complementary strengths of FoldX-MD (Miller et al., 2016; Sapozhnikov et al., 2023; Barnes et al., 2022) and Rosetta-FlexddG (Fuchs et al., 2015; Barlow et al., 2018), was applied. FoldX-MD samples hundreds of WT MD trajectory snapshots, capturing the average dynamic energetic landscape, providing high sensitivity toward steric or electrostatic disruptions caused by residue substitutions (Potapov et al., 2009; Guerois et al., 2002). Meanwhile, Rosetta-FlexddG incorporates local backbone flexibility offering a conservative but often physically realistic estimate of structural tolerance while identifying stabilizing mutations more effectively than FoldX-MD (Barlow et al., 2018; Buß et al., 2018). Because each method captures distinct energetic features, variants flagged by either approach were retained to minimize false negative predictions.

Binding affinity differences (${\mathrm{\Delta \Delta G}}_{\mathrm{bind}}$) were calculated using (Eq. (1):

(1) $$∆∆G=∆G_{\mathrm{variant}}-∆G_{\mathrm{WT}}\;\left[{\mathrm{kcal}}^{-1}\right]$$

where a positive ΔΔG value represents a destabilizing mutation (van Durme et al., 2011; Schymkowitz et al., 2005a). ΔG_*WT*_ and ΔG_*variant*_ stand for the estimated binding affinity values of the WT system and variant system, respectively. Missense variants were classified based on energetic thresholds as significantly destabilizing (ΔΔG ≥ 1.0 kcal/mol), mildly destabilizing (0.5 < ΔΔG < 1.0 kcal/mol), and neutral/tolerated (ΔΔG ≤ 0.5 kcal/mol). A subset of variants showing predicted energetic perturbations (ΔΔG ≥ 0.5 or ≤ –0.5 kcal/mol) in either protocols (*N* = 33; Fig. 1D) were selected for MD simulations (*N* = 27), ensuring the inclusion of variants with potential destabilizing or stabilizing binding effects (Fig. S2).

##### FoldX-MD workflow

Folding stability $\;{\mathrm{\Delta \Delta G}}_{\mathrm{fold}}$ and binding affinity changes (${\mathrm{\Delta \Delta G}}_{\mathrm{bind}}$) were estimated for WT and missense variants using the FoldX 5.1 empirical force field (van Durme et al., 2011; Schymkowitz et al., 2005b). Following the removal of solvent, ions, and GTP, representative snapshots were extracted at 1-ns intervals of each of the initial three 200-ns (discarding the first 20 ns) SynGAP–Ras and SynGAP–Rap WT MD replicas, together with 200 snapshots spanning 1–200 ns of each of the later 1-μs WT production simulations (Section 0) using CPPTRAJ (Aktulga et al., 2022). Snapshots were then optimized using six *RepairPDB* energy-minimization cycles to ensure energy convergence. Missense variants (*N* = 214) were generated, and their folding stability (${\mathrm{\Delta \Delta G}}_{\mathrm{fold}}$) was calculated using *BuildModel* with each optimized WT structure as a template. Binding affinity (${\mathrm{\Delta \Delta G}}_{\mathrm{bind}})\;$were calculated using *AnalyseComplex* (Fig. S5). The modified MD protocol for FoldX has been introduced in prior studies (Miller et al., 2016; Sapozhnikov et al., 2023; Barnes et al., 2022; Yu et al., 2015; Gonzalez et al., 2020; Chitongo et al., 2020; Hummer et al., 2025).

##### Rosetta-FlexddG protocol

To account for backbone flexibility, the Rosetta-FlexddG (Barlow et al., 2018; Fleishman et al., 2011; Leman et al., 2020; Leaver-Fay et al., 2011; Alford et al., 2017; Chaudhury et al., 2010) was employed on a curated subset of missense variants (*N* = 73 vs. 214 processed with FoldX-MD) filtering out neutral variants (ΔΔG ≈ 0 kcal/mol) predicted by FoldX-MD. Three WT templates per complex were extracted from the initial (Section 0.) 200-ns MD replicas (SynGAP–Ras: 67.6 ns, -123.15 kcal/mol; 129.56 ns, -88.14 kcal/mol; 91.69 ns, -123.75 kcal/mol; SynGAP–Rap: 150.90 ns, -128.99 kcal/mol; 23.79 ns, -139.37 kcal/mol; 143.79 ns, -94.9 kcal/mol). These frames represent local minima in the MM/GBSA energy landscape and were used as stable starting structures. Each template underwent 20 iterative rounds of full-atom *FastRelax* (excluding water, ions, and GTP), generating 50 independent models per each relaxed structure to capture conformational variability. The lowest-energy relaxed structure per template was initially selected for ΔΔG calculations. Due to interfacial distortions in the SynGAP–Ras replica-2 frame, a successor structure from replica-3 was substituted to ensure structural integrity. Flexibility within an 8 Å radius of the mutation site was sampled using the Backrub algorithm (stride 35,000; 35 iterations) under the *ref2015* scoring function (Barlow et al., 2018). Rosetta Energy Units were scaled to kcal/mol (factor: 2.94) using the RosettaDDGPrediction wrapper. All calculations were executed using the *flexddg_ref2015.yaml* protocol and aggregated using *aggregate.yaml* provided by using the RosettaDDGPrediction Python wrapper (https://github.com/ELELAB/RosettaDDGPrediction) (Sora et al., 2023). While Rosetta's backbone relaxation typically yielded smaller ΔΔG magnitudes than FoldX-MD, the discrete template sampling (*N* = 3) occasionally obscured specific steric or electrostatic perturbations (e.g., R479Q, P600V; Table 1 and S2).

### Trajectory analysis and figure preparation

The MD simulation trajectories were processed with CPPTRAJ in AMBERTOOLS22 (Aktulga et al., 2022) and analyzed using BODIL (Lehtonen et al., 2004), VMD 1.9.4a12 (Humphrey et al., 1996), and PYMOL 2.6.0 (Schrodinger, 2019). The inter-protein and GTP-protein hydrogen bonding (or H-bonding), catalytic water occupancy at the active-site (Fig. S6), and RMSD (Root-Mean-Square-Deviation) calculations for protein backbone and GTP (Figs. S7–S9) were done using CPPTRAJ. The normalized B-factors (Fuchs et al., 2015) were calculated to measure the local fluctuations of the protein backbone and GTPase-bound GTP during the MD simulations (Fig. S10). The overall system stability was assessed by examining the protein's backbone RMSD curves, energy profiles, and visual inspection of trajectories. Figures were rendered with PYMOL and VMD. Selected simulation frames used in figures are made available online via the SGM server (https://syngapmissenseserver.utu.fi/).

## Results & discussion

### Canonical RasGAP-like deactivation of Ras-GTPase by SynGAP

Earlier studies suggested that the Ras-GTPase follows an S_N_2-type mechanism proceeding through a concerted pathway involving a loose transition state, without a metaphosphate intermediate (Admiraal and Herschlag, 1995; Du and Sprang, 2009; Du et al., 2004; Lassila et al., 2011). Regarding the attacking water molecule, it was proposed that GTP itself may activate it via H-bonding (Langen et al., 1992; Schweins et al., 1994), or that catalysis takes place without an activating base, with nearby residues instead positioning the attacking water and facilitating proton removal after the transition state (Lassila et al., 2011). Later QM/MM studies still support an S_N_2-type pathway, but Gln61 acting as an activating base for water (Fig. 2A) (Berta et al., 2023). Furthermore, RasGAPs catalyze hydrolysis through a conserved Arg-finger that stabilizes the transition state and promotes γ-phosphate release (Fig. 2A-B). Indeed, mutations at either residue, Gln61 or Arg-finger, disrupt GTP hydrolysis and lock Ras in its non-enzymatically active state (Zhu et al., 2002; Kosloff and Selinger, 2003; Kö et al., 2008; Bos et al., 2007; Martín-García et al., 2012). Lastly, QM/MM studies have suggested that the catalytic glutamine would undergo amide to imide tautomerization during GTP hydrolysis at least in RhoGAP–RhoA complex (Pardos et al., 2025; Parise et al., 2026). Regardless, the classical MD approach applied in this study is not addressing transition state but instead focuses solely on the pre-reaction state.

During the initial 200-ns MD simulations, performed to validate the modelled WT-SynGAP–Ras complex (Fig. 2B) and identify representative low-energy conformations for subsequent in silico mutagenesis, the Arg-finger (Arg485) remained engaged with the γ-phosphate of Ras-bound GTP, while the catalytic water was stably positioned for nucleophilic attack. Furthermore, Arg485 and Ras-Gln61 maintained catalytically competent geometries for an average of ∼90% of the simulation time across three independent replicas (Table S3). These results confirmed that the equilibrated complexes could retain long enough the key structural features required for catalysis and provided suitable template structures for missense mutation modelling and subsequent variant (and equivalent WT) simulations. Consistent with canonical RasGAP–Ras complexes, the Ras-Switch I and II regions became rigidified through extensive stabilizing interactions (Scheffzek et al., 1997; Vetter and Wittinghofer, 2001; Milburn et al., 1990; Scheffzek and Shivalingaiah, 2019) (Figs. 5A-C, 6A-C, S11, and S12), while the SynGAP-C2 domain provided additional peripheral support (Fig. 6C).

**Fig. 5:**
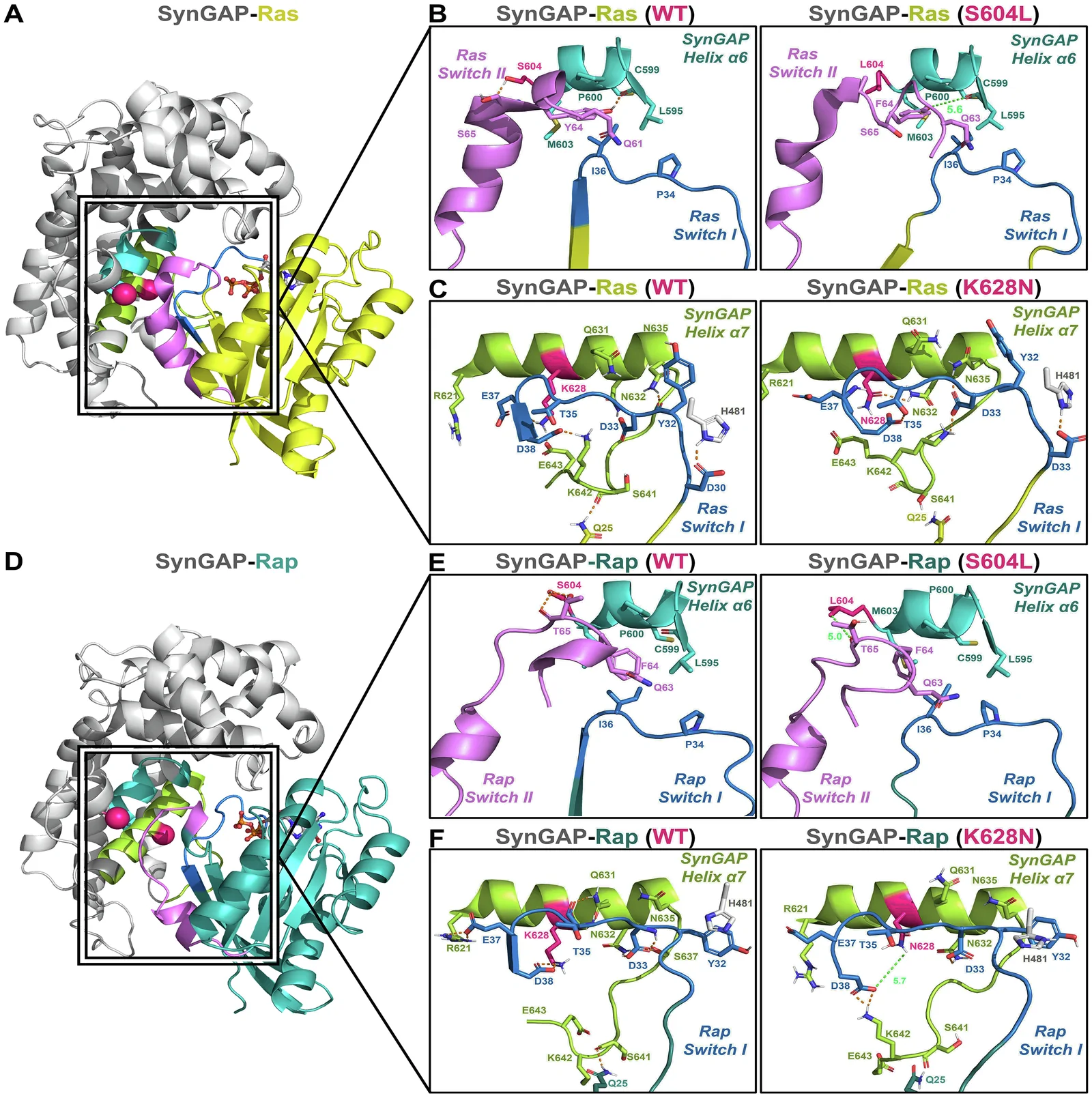
**SynGAP missense mutations S604L and K628N at helices α6 and α7 disrupt SynGAP–GTPase interactions. (A)** In the SynGAP–Ras complex (gray/yellow cartoon), helix α6 (cyan) and helix α7 (lime) stabilize the interface with Ras-Switch I (blue) and II (purple). **(B)** In the WT close-up, helix α6 secures Switch positioning through hydrophobic packing and a defined H-bond network (orange dotted lines); Ser604 (magenta carbons) and Leu595 (sticks with white carbons) form stabilizing H-bonds with Ras-Ser65 and -Tyr64 (purple carbons), respectively. Substitution of Ser604 with leucine introduces a bulkier hydrophobic side-chain that disrupts this arrangement and induces a subtle structural shift that weakens the interface. **(C)** In the WT close-up, helix α7 forms a broad stabilization platform for Switch I, where Lys628 (magenta carbons), Gln631, Asn635, Lys642, and Glu643 (lime carbons) establish a dense network of H-bonds and salt-bridges (orange dotted lines) with Ras residues Asp33, Thr35, Asp38, and Glu37 (blue carbons), while His481 (white carbons) provides distal stabilization via an H-bond with Ras-Asp30. The K628N missense mutation shortens the side-chain and disrupts this central anchor, destabilizing the protein-protein interface. **(D)** The SynGAP–Rap overview (gray/cyan cartoon) similarly positions helices α6 and α7 adjacent to the Rap-Switch regions. **(E)** In the WT close-up, Ser604 forms polar contacts with Rap residues Thr65 and Phe64 that help maintain a catalytically competent Switch II conformation; the S604L missense variant disrupts these interactions and impairs Switch II orientation. **(F)** In the WT close-up, helix α7 residues establish a network of polar and electrostatic interactions with Rap-Switch I, including a key contact between Lys628 and Rap-Asp38; the K628N missense variant fails to maintain this interaction, compromising interface stability and Switch I positioning. Key distances are indicated by green dashed lines. A representative 100 ns MD snapshot is shown for each system. (For interpretation of the references to colour in this figure legend, the reader is referred to the web version of this article.)

**Fig. 6:**
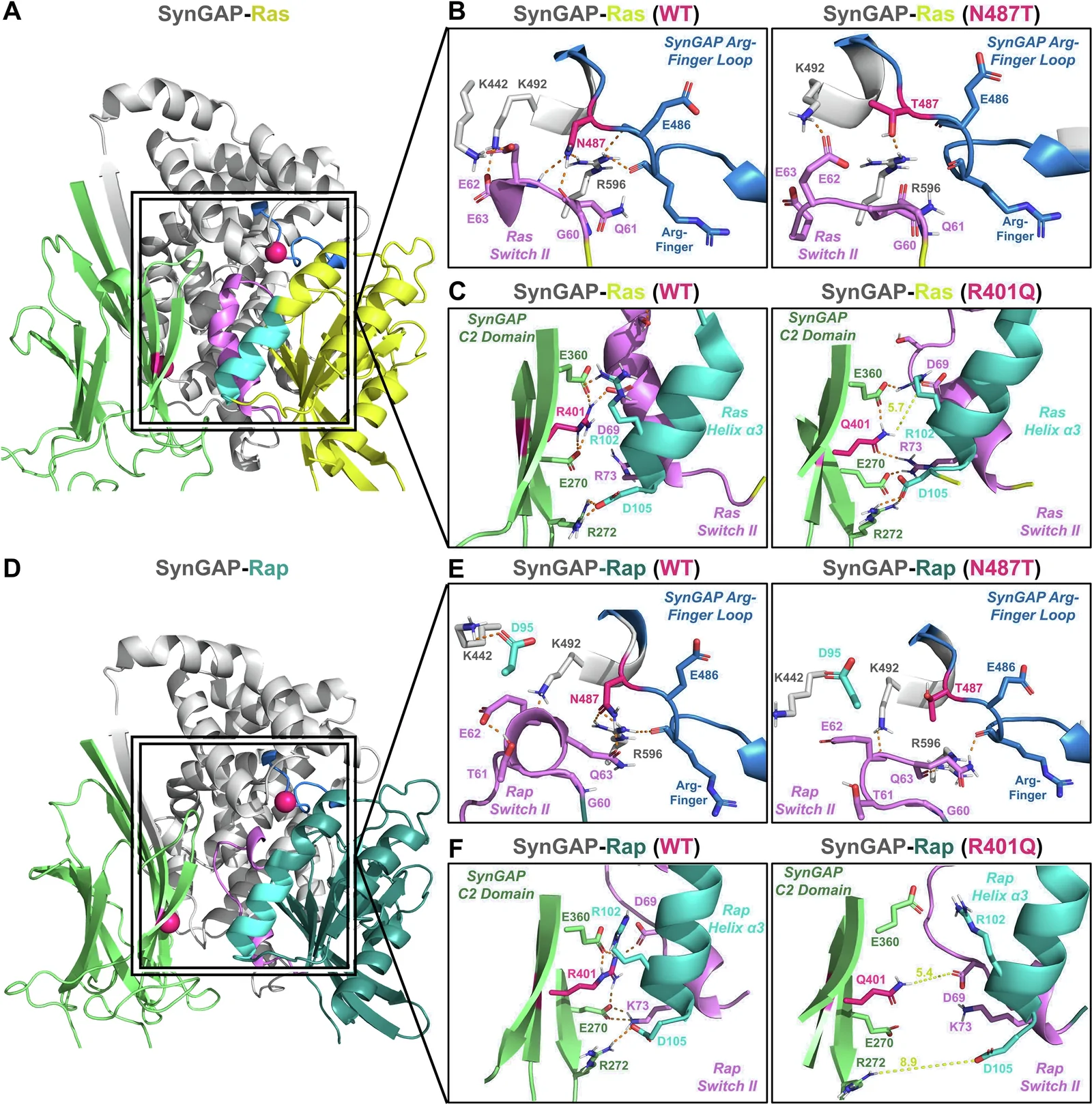
**SynGAP missense mutations R401Q and N487T disrupt Switch II stabilization and perturb coordination of catalytic residues. (A)** Overview highlighting Arg401 in the C2 domain (green) and Asn487 in the Arg-finger loop (blue) as magenta spheres; the C2 domain stabilizes GTPase helix α3 (cyan) and Switch II (purple), while Asn487 contributes to Switch II stabilization. **(B)** In the WT close-up, Asn487 (sticks with magenta carbons) stabilizes the active-site by forming key H-bonds (orange dotted lines) with Ras residues Glu62 and Gly60 (purple carbons). Together with Arg596, Lys492, and Lys442 (white carbons), this network maintains the proper orientation of the Arg-finger loop and catalytic Gln61 (purple carbons). The N487T substitution disrupts anchoring to Glu62 and Gly60, increasing loop flexibility and shifting Gln61, likely reducing catalytic efficiency. **(C)** In the WT close-up, Arg401 (magenta carbons) acts as a central electrostatic hub, forming a salt-bridge (orange dotted lines) with Ras-Asp69 (purple carbons) and coordinating with SynGAP residues Glu360 and Glu270 (green carbons). This network is further supported by interactions with Ras helix α3 residues Arg102 and Asp105 (cyan carbons) and Switch II residue Arg73. The R401Q missense mutation removes the positive charge, disrupts the salt-bridge with Ras-Asp69, and destabilizes the C2–GTPase interface. **(D)** The SynGAP–Rap complex overview (white/teal cartoon) similarly highlights the positioning of Arg401 at the C2–Rap interface and Asn487 at the catalytic center. **(E)** In the WT close-up, catalytic Gln63 (purple carbons) and the Arg-finger are precisely positioned by a polar network involving Arg596, Lys492, and Lys442; the N487T variant disrupts stabilization of Rap-Switch II, perturbing the catalytic environment (see Fig. 3). (F) In the WT close-up, Arg401 stabilizes the interaction with Rap-Asp69 (purple carbons) and coordinates surrounding charged residues to anchor the C2 domain to the Rap surface; the R401Q missense variant disrupts this network, causing structural drift and reduced interface stability. (For interpretation of the references to colour in this figure legend, the reader is referred to the web version of this article.)

At this initial system build-up and validation stage, the generated SynGAP–Ras model (Fig. 2B) retained active-site geometry and protein-protein interactions expected for a pre-reaction state of functional RasGAP, supporting its use as a basis for in silico mutagenesis and further in-depth WT system analysis. Nonetheless, because SynGAP is a dual-specificity GAP that inactivates both Ras- and Rap-GTPases, while holding a canonical RasGAP structural fold (Kupzig et al., 2006; Pena et al., 2008; Qiao and Homayouni, 2015; Kupzig et al., 2009), next, it was necessary to construct and validate the SynGAP–Rap model.

### Plexin-like non-canonical deactivation of Rap1b-GTPase by SynGAP

Rap-GTPases deactivation is expected to follow a catalytic mechanism similar to the pathway described for Ras (Fig. 2A). However, where traditional RapGAPs utilize an Asn-thumb, analogous to Ras-Gln61, to coordinate GTP hydrolysis and a “closed” Rap-Tyr32 conformation that stabilizes its leaving group (Scrima et al., 2008; Daumke et al., 2004), SynGAP relies on a non-canonical Arg-finger mechanism for Rap activation. While mutation of the Arg-finger severely impaired both Ras- and Rap-GAP activities, systematic scanning of asparagine residues failed to identify an essential Asn-thumb, as none of the substitutions substantially affected Rap-GTP hydrolysis (Kupzig et al., 2006; Kupzig et al., 2009). Instead, Rap is proposed to undergo a conformational shift allowing Rap-Gln63 to mimic the catalytic role of Ras-Gln61, a hypothesis supported by the significant loss of activity caused by the Q63A mutation with the dual-specificity GAPs (Sot et al., 2010).

To characterize this association at the structural level, the WT-SynGAP–Rap1b complex was modelled using the PlexinC1–Rap1b complex (PDB: 4M8N (Wang et al., 2013); Fig. 2C) as a template, featuring a Switch II hairpin that orients Gln63 toward the γ-phosphate of GTP, analogous to the Ras-Gln61 and Asn-thumb, and maintains Tyr32 in an “open” state to avoid steric clashes (Fig. 2C vs. Fig. S1). During the initial 200-ns MD simulations, performed to validate the SynGAP–Rap1b model and identify representative low-energy conformations for subsequent in silico mutagenesis, Arg485 remained stably engaged with the γ-phosphate, while Rap-Gln63 exhibited higher flexibility than Ras-Gln61 yet, it consistently remained extended and within the active-site. This flexibility influenced catalytic water coordination and produced relatively high standard deviation across replicas (55.2% ± 39%; Table S3); however, its significance should not be overinterpreted, as it may simply reflect that the SynGAP–Rap complex had to be modelled from the Plexin–Rap template instead of relying on closer homolog structure.

### GTPase active-site stability during extended simulations defines the analysis window

Following establishment of the WT-SynGAP–GTPase models, representative low-energy structures were selected from the initial MD simulations based on MM/GBSA interface energy and preservation of the catalytically competent active-site arrangement (Fig. 2B-C). These structures were then used as common templates for in silico mutagenesis and for the corresponding WT systems that served as references for subsequent comparative simulations (Fig. S2). The stability of the catalytically competent WT state over extended simulation times was next examined to assess both overall complex stability and the active-site geometry or competency.

Although both WT-SynGAP–Ras/Rap-GTPase complexes remained associated throughout the 1 μs simulations (Fig. S7), gradual deterioration of the catalytically relevant geometries and other features characteristic of the pre-reaction state were eventually observed, suggesting limited stability of the enzymatically competent configurations in classical MD simulations. Specifically, the catalytic glutamine residue (Ras-Gln61 or Rap-Gln63), which is critical for positioning the nucleophilic water molecule during GAP-mediated GTP hydrolysis, did not permanently maintain its reaction-competent orientation during extended simulations (Fig. 4A-B). Over time, the side-chain at least occasionally rotated away from the active-site, disrupting the GTP-water-glutamine coordination network or water-bridge (Fig. 4C). The timing of this transition or its permanence varied between the two GTPases and three simulation replicas. The SynGAP–Ras systems lost the water-bridge after ∼400 ns in two of the replica simulations, although the connection was intermittently upkept or re-established in the third one (Fig. 4D). With the SynGAP–Rap systems the water-bridge arrangement was far more dynamic or labile from the start but, unlike with the SynGAP–Ras complex, it was also more regularly re-established (Fig. 4D). In contrast, the SynGAP-Arg-finger remained permanently interacting with GTP throughout the trajectories.

Notably, both the stable Arg-finger positioning and gradual loss of the catalytic glutamine orientation in the SynGAP–GTPase simulations mirrored a recent MD simulation study of the GAP–HRas complex by Patel et al. (Sapozhnikov et al., 2023). During the simulations, the HRas active-site structure relaxed away from the original X-ray crystal arrangement and lost the GTP–glutamine contact in 9 of 10 independent 1 μs replicas. The HRas-Gln61 side-chain shifted from an enzymatically competent distance of <4 Å to a non-competent position >9 Å, and, thus, the catalytically competent state was maintained for only ∼15% of the total simulation time.

Several factors may contribute to the degradation or loss of the catalytically competent arrangement at the Ras- and Rap-GTPase active-sites with SynGAP, including inevitable inaccuracies in the complex starting geometry, limitations of the employed force field parameters (e.g., Mg^2+^, water model). However, as discussed by Patel et al. (Patel et al., 2023), the catalytically active glutamine conformation is likely inherently transient and, therefore, should not be expected to remain locked in a productive arrangement over extended timescales even with bound GAP. This interpretation is further supported by ground-state Ras–GTP analog structures, which lack the highly constrained active-site geometry observed in transition-state mimic complexes containing GDP/AlF₃ (Rabara et al., 2019; Yan et al., 2020). Importantly, despite the gradual relaxation of the catalytic glutamine orientation, overall, the SynGAP–GTPase complexes remained structurally stable throughout the 1 μs simulations (Fig. S7).

Based on these observations, 0 to 500 ns was considered a reasonable analysis window for both WT and subsequent missense variant simulations as it balances conformational sampling with preservation of a catalytically competent active-site geometry.

### The stabilization of GTPases: Key contributions from the α6 helix, α7 helix and C2 domain of SynGAP

While maintaining a catalytically competent active-site arrangement is essential, disease-associated SynGAP missense mutations are distributed across the entire SynGAP–GTPase interface (Fig. 1D). Consequently, these variants may impair complex formation and stability rather than directly disrupting catalytic competency. Therefore, next, stabilizing interactions across the broader interface were examined to assess whether the SynGAP–Ras and SynGAP–Rap models within the 0-500 ns simulation window provide a reliable framework for the in silico mutagenesis analyses (Section 0). Although the primary analysis focus was on the first 500 ns, interaction stability and persistence over the full 1 μs trajectories were also considered and are discussed where relevant.

#### Stabilization of the WT-SynGAP–Ras Interface

Ras-Switch I is stabilized through hydrophobic packing between Ras-Pro34/Ile36 and SynGAP-α6 helix residues Leu595, Cys599, Pro600, and Met603 (Fig. 5B), reinforced by salt-bridges from Lys628 and Lys642 to Ras-Asp33 and -Asp38, respectively. Lys642 and Glu643 form an electrostatic clamp that anchors the SynGAP-α7 helix, with further contributions from Asn635 and His481 (Fig. 5C). Switch II stabilization arises from Ras-Tyr64 packing against α6 helix and H-bonding involving Ras-Leu595. Moreover, transient Ser604–Ras-Ser65 interactions (Fig. 5B), and salt-bridges/H-bonds from Asn487/Lys442/Lys492/Arg596 to Ras-Gly60/Glu62/Glu63, maintaining the geometry around Ras-Gln61 (Figs. 6B, S13A, and S14). Additional interface stability is provided by the C2 domain through favorable electrostatic interactions between SynGAP residues Arg272, Glu360, and Arg401 and their Ras counterparts Asp105, Gln99, and Arg102 (Fig. 6C) (Scheffzek et al., 1997; Pena et al., 2008; Paul et al., 2025a).

Notably, the onset of Gln61 relaxation at ∼400–500 ns coincided with the progressive disruption of the supporting interaction network involving Asn487, Lys442, and Lys492, as well as weakening of the C2 domain–Ras contact in two of the three replicas. Because these changes emerged only during the latter stages of the simulations, they further support the selection of the 0–500 ns interval as the primary analysis window.

#### Stabilization of the WT-SynGAP–Rap1b Interface

PlexinC1 stabilizes the extended Rap-Switch II conformation through a H-bond network involving Arg1001, Asn1005, and Asn1009, that orients Rap-Gln63 and anchors the Arg-finger loop. Mutations of these residues severely impair Plexin-induced GTP hydrolysis in vitro, underscoring their critical catalytic role (Wang et al., 2013; Rohm et al., 2000; Wang et al., 2012).

While SynGAP lacks some of the Switch II stabilizing residues of Plexin (Asn1005, Asn1009; Fig. 3B–D), WT-SynGAP–Rap1b simulations suggest a plausible compensating stabilization network. In this network arrangement, SynGAP-Arg596, analogous to Plexin-Arg1001, stabilizes the Arg-finger loop, while Asn487 and Lys492 coordinate the Rap-Switch II backbone to maintain Gln63 within the active-site. Moreover, Arg596 indirectly supports Rap-Switch II positioning by coordinating Asn487 placement (Figs. 6D–E, S13B, and S14). Similar to SynGAP–Ras complex, the stabilizing interactions between SynGAP-Asn487/Lys492 and Rap-Switch II progressively weaken over the course of the simulations, likely contributing to increased Switch II mobility and relaxation away from the catalytically competent conformation. Additionally, Ser604, at the position equivalent to the Plexin-Asn1009, interacts with the backbone atoms of Rap-Switch II further stabilizing the extended conformation (Fig. 5D-E). The residue corresponding to SynGAP-Ser604 is conserved among dual-specificity GAPs, whereas Rasal3 contains alanine at the equivalent position and lacks Rap-GTPase activity in vitro, suggesting that this sequence difference may be functionally relevant (Fig. 3B) (Saito et al., 2015; Muro et al., 2015).

SynGAP-helix α6 further stabilizes Switch II through refined hydrophobic packing with the Rap residues Phe64, Pro34, and Ile36 based on the simulations (Fig. 5E). Furthermore, the C2 domain provides critical contacts that contribute to the stabilization of the complex during the simulations through a robust interprotein salt-bridge network, involving Arg272, Glu360, and Arg401, with Arg401 anchoring the extended Rap-Switch II geometry. Here, the Arg401–Asp69 salt-bridge, a highly stable interaction between SynGAP and Rap, is maintained throughout all three 1 μs replicas (Figs. 6F and 3D). This interaction is critical for the GTPase deactivation, as the R401S mutation has been shown in vitro to completely abolish GTP hydrolysis (Paul et al., 2025a). Moreover, this modelling result is in line with the essential role of the C2 domain that has been reported for the SynGAP-mediated Rap-GTPase activity based on in vitro experiments (Pena et al., 2008; Paul et al., 2025a).

In Plexin, an “activation segment” has been experimentally established to sterically regulates Rap1b access by transitioning from a “closed” to an “open” conformation upon dimerization (Wang et al., 2013). While the equivalent trigger in SynGAP has not been established, it could, if required at all, involve for example post-translational phosphorylation. Regardless, modelling revealed an analogous clash between the SynGAP-helix α7-loop segment (residues 628-645) and Rap1b-Switch I. This segment, referred hereafter as “SynGAP-activation segment”, was repositioned in the modelled complex into an open conformation analogous to the active Plexin pose (Fig. 3E), establishing several subsequent stabilizing SynGAP–Rap contacts: Lys628-Asp38 and Arg621-Glu37 salt-bridges, as well as Ser637-Asp33 and Ser641-Gln25 H-bonds. Additionally, His481 stacks with the open Rap-Tyr32 to stabilize this state (Fig. 5F). Highlighting the potential importance of these modelled interactions for SynGAP, disrupting the equivalent activation-segment contacts in Plexin have been determined in vitro to impair its Rap-GTPase activity (Wang et al., 2013).

Collectively, the MD simulations suggest which explicit SynGAP–Rap interface contacts stabilize the extended Rap-Switch II conformation and, thus, could compensate for the missing Plexin residues, ensuring a catalytically competent pre-reaction placement of Rap-Gln63. Encouraged by these interface stability results for the 500 ns time window; next the analysis focused on determining how SynGAP could act as dual-GTPase activator and differentially engage Ras and Rap.

#### Modelling highlights the structural differences in SynGAP engagement of Ras- and Rrap-GTPases

Based on the WT-SynGAP–Rap simulations, unlike in the SynGAP–Ras complex where Lys442 predominantly interacts with Ras-Glu62, with Rap, Lys442 switches salt-bridging between Rap-Glu62 and Rap-Asp95, alternating between these two contacts throughout the 1-μs simulations. This modelled interaction is potentially important for Rap binding, consistent with the analogous and experimentally verified Plexin–Rap1b set-up (Wang et al., 2013). The modelled ionic interaction would be unique to Rap, as Ras carries an asparagine at the equivalent position (Fig. 6B vs. 6E). Likewise, SynGAP–Ras modelling identifies Glu486 as a potentially critical anchor coordinating with Ras-Lys88/Asn86, while maintaining an intramolecular charge pair with Arg479 (Figs. S15A–B, S16 and S17). Alternatively, based on the simulations, Rap-Glu123 recruits Arg479 into an intermolecular salt-bridge, while Glu486 engages Arg475 and Rap-Ser88 (Figs. S15C–D and S16 and S17).

Based on the WT simulations, the SynGAP activation segment residues Lys642 and Glu643 contribute to SynGAP–Ras complex stability but do not maintain persistent contacts with Rap1b throughout the full 1 μs trajectories. Instead, the modelling shows that the segment adopts an open conformation farther from the Rap-GTPase (Fig. 5C vs. 5F). In this modelled arrangement, SynGAP-Lys642 is analogous to Plexin-Lys1047 that has been established to contribute to the dimerization-dependent regulation rather than monomeric GAP–GTPase binding (Wang et al., 2013), suggesting a potential role in additional protein–protein interactions relevant to the Rap-GTPase activity regulation. Nevertheless, because the SynGAP-activation loop was modelled from Plexin, its true positioning in the SynGAP–Rap complex could differ in reality.

Overall, the modelling provides a plausible structural basis for SynGAP's ability to bind both Ras and Rap by reconfiguring its contact interface and charge networks.

### Missense mutations at the SynGAP–GTPase Interface disrupting the complex stability

To identify SynGAP missense mutations that perturb GTPase regulation, 214 missense variants were compiled from cancer datasets and the literature, 121 of which (e.g., R401Q, R596H, and S604L) overlap with the ClinVar-listed SRD entries (Fig. 1E). This cancer–SRD overlap (e.g., Arg272, Arg401, Arg596, Lys628) (Scheffzek et al., 1997; Paul et al., 2025b), implies that although GTPase dysregulation may not be the primary driver of the core behavioral SRD phenotype (Araki et al., 2024; Brill et al., 2025), it could be a contributing factor influencing a sizable subset of patients.

The dual FoldX-MD/Rosetta interface-energy protocol was applied to prioritize interface-facing candidate missense variants for more detailed classical MD simulation analysis (Fig. 1D). The predictive value of the approach was supported by its successful identification of experimentally validated functional residues, including Arg401 (Pena et al., 2008; Paul et al., 2025a), Arg596 (Wang et al., 2013; Wang et al., 2012), and Pro600 (Wang et al., 2013; Kupzig et al., 2009) (Table 1). The protocol also captured differential specificity; for example, Arg401 variants (e.g., R401Q) were predicted to destabilize the SynGAP-Rap1b complex (ΔΔG = 3.9 kcal/mol) more strongly than the SynGAP–Ras complex (ΔΔG = 1.6 kcal/mol; Table 1), consistent with the established role of Arg401 in Rap-specific GAP activity (Paul et al., 2025a). A small subset of variants showed substantial divergence between replica energy calculations, reflected in distinct interaction-energy profiles (Fig. S5) and high standard deviations relative to the mean values (Tables 1 and S2). This variability highlights a limitation of mutation-effect analyses based on alternative WT conformations, as the predicted energetic impact can depend on the local structural state used as the mutagenesis template. To account for this uncertainty, variants were assigned three-tier confidence annotations based on replica consistency (Tables 1 and S2). Only a small number of variants showed complete disagreement in the direction of the predicted energetic effect, including E360K (with Ras; Rosetta), N487K (with Rap; Rosetta), and S604L (with Ras; FoldX-MD), and were therefore classified as uncertain.

27 of SynGAP–Ras/Rap variant systems were prioritized based on the interaction energy calculations (ΔΔG ≥ 0.5 or ≤ −0.5 kcal/mol) and, thus, put forward for in silico mutagenesis using the representative low-energy WT complex structures as common templates (Section 0.). These variant systems were initially MD simulated, alongside the corresponding WT systems, in three independent 100-ns replicas to screen for mutation-induced structural changes flagged by the interface energy calculations. Although backbone RMSD and MM/GBSA interface energies showed initial fluctuations before stabilizing, the introduced mutations did not substantially disrupt the overall integrity of the SynGAP–GTPase complexes (Table S4; Figs. S3 and S8). In contrast, local structural inspection revealed apparent variant-induced perturbations already within the first 100 ns, and several variants also showed reduced catalytic-water coordination relative to the WT systems (Table S3). Based on this initial assessment, the simulations were extended to 500 ns for 14 of the variants in order to determine whether the observed early-stage perturbations persisted or developed further and to provide broader sampling for structural and energetic characterization against the WT systems within the same timeframe.

None of 14 selected missense mutations induced full complex dissociation even during the extended 500-ns simulations (Fig. S3). The most prominent energetic perturbations in variant simulations localized near Switches I and II (Table 1; Fig. 1D), correlating with elevated B-factor atomic fluctuations in these regions (Fig. S10). Furthermore, certain missense mutations (e.g., N487K, K492E, and S604L) were found to weaken specifically the catalytic water coordination, suggesting reduced active-site stability and lower hydrolysis efficiency (Fig. S6; Table 2). Perplexingly, despite the markedly impaired complex formation energies predicted by the FoldX-MD/Rosetta analyses (Fig. S5; Table 1), some variants, such as E486K and H481D, retained catalytic-water coordination comparable to, or in certain simulation replicas even more stable than, that observed for the WT protein (Fig. S6). This apparent enhancement beyond WT behavior is likely a modelling artifact arising from compensatory local interactions that transiently stabilize catalytic water positioning or reduce conformational flexibility within the active-site pocket. Hence, these observations alone should not be interpreted as strong evidence that the variants are functioning normally, as the unfavorable interaction-energy calculations still point to an overall detrimental effect.

**Table 2:** Percentage of coordinated water at the SynGAP-induced Ras/Rap-GTPase active-site between catalytic glutamine and GTP.

Variant	SynGAP–Ras Complex		SynGAP–Rap Complex	
	% of Coordinated Water Bridge^1^	%Average	% of Coordinated Water Bridge^1^	%Average
WT- #1	71.7	77.6 ± 9.6	68.9	56.7 ± 21.5
WT-#2	69.8		74.7	
WT-#3	91.2		26.5	
R401Q-#1	24.6	70 ± 32.1	61.2	**24.7 ± 25.9**
R401Q-#2	92.3		7.6	
R401Q-#3	93.2		5.2	
R479Q-#1	38.6	**46.3 ± 5.4**	53	51.7 ± 27.8
R479Q-#2	50.2		17.1	
R479Q-#3	50		85.1	
H481D-#1	37.8	77.5 ± 28.1	19.4	52.6 ± 25.2
H481D-#2	98.7		80.3	
H481D-#3	96.1		58	
R485L-F484A-#1	94.3	**58.9 ± 35.2**	36.7	43.4 ± 16.7
R485L-F484A-#2	71.6		27.2	
R485L-F484A-#3	11		66.3	
R485K-#1	74.2	**64.6 ± 31**	22.8	**23.4 ± 4**
R485K-#2	96.9		18.8	
R485K-#3	22.8		28.5	
R485P-#1	26.6	**52.9 ± 24.1**	3.5	**23.8 ± 14.7**
R485P-#2	84.9		30.2	
R485P-#3	47.2		37.7	
E486K-#1	99.3	78.1 ± 26.1	58	47.4 ± 13
E486K-#2	93.7		55.2	
E486K-#3	41.3		29.2	
N487K-#1	63.4	76.7 ± 15.3	30.8	53.8 ± 16.5
N487K-#2	98.1		68.1	
N487K-#3	68.6		62.6	
N487T-#1	**57.2**	**62.5 ± 15.4**	41.5	**22.9 ± 13.7**
N487T-#2	46.8		8.8	
N487T-#3	83.4		18.5	
K492E-#1	93.3	**54.3 ± 29.8**	54.3	45.6 ± 11.5
K492E-#2	21		53.1	
K492E-#3	48.5		29.3	
R596H-#1	19.4	**33 ± 9.8**	18.8	**26.7 ± 11.4**
R596H-#2	37.7		18.3	
R596H-#3	41.8		42.8	
P600V-#1	86.2	89.4 ± 2.3	16.6	**31.3 ± 24.3**
P600V -#2	91.5		65.6	
P600V-#3	90.6		11.7	
S604L-#1	33.3	**34.9 ± 7.6**	12.5	**33.2 ± 30.5**
S604L-#2	26.6		76.3	
S604L-#3	44.9		10.8	
K628N-#1	6.3	**43.5 ± 26.57**	31.7	**20.4 ± 9.1**
K628N-#2	57.7		9.3	
K628N-#3	66.6		20.4	

Percentage of water molecule being coordinated at the catalytic position between GTP and residues Gln61 (SynGAP–Ras) or Gln63 (SynGAP–Rap) across three 500-ns replica MD simulation trajectories (Fig. 4 A and B). Values represent averages over three (#1, #2 and #3) independent replicas. Values **±** differing from the WT standard deviation are highlighted in bold. H-bond distances and angles were computed using the *hbond bridgeout* in CPPTRAJ (Case et al., 2023b), considering the water molecule closest to the GTP γ-phosphate at each simulation frame. Water behavior was evaluated using:

1 Coordinated-water criterion: Water classified as coordinated only when simultaneously satisfying both the H-bond angle and distance requirements (≤3.6 Å heavy-atom distance and ≥ 135° Acceptor–H–Donor angle) between the catalytic Gln and GTP.

Overall, the fact that the most prominent variant effects were centered on the Switches in the simulations aligns well with the experimentally established role of GAPs in stabilizing the inherently mobile Switch regions to orient the catalytic glutamine (Scheffzek and Shivalingaiah, 2019). However, focusing solely on the complex formation interactions and energy or binding affinity predictions would overlook distal, allosteric or folding stability-related effects of missense mutations (Barlow et al., 2018; Potapov et al., 2009; Wodak et al., 2019). For instance, FoldX-MD approach identified 39 mutations (e.g., G344S: ${\mathrm{\Delta \Delta G}}_{\mathrm{fold}}$ = 7.25 kcal/mol vs. ${\mathrm{\Delta \Delta G}}_{\mathrm{bind}}$ = 0.00 kcal/mol; Table S1) that are predicted to markedly destabilize the protein fold (${\mathrm{\Delta \Delta G}}_{\mathrm{fold}}\;$> 3 kcal/mol; Table S1) despite having no apparent effect for the binding energy based on the affinity calculations. In other words, fold-disrupting missense variants could impair GAP function through the loss of SynGAP structural integrity rather than merely causing local interface disruptions.

#### Variants in the helical platform: Disrupting Switch I/II stabilization and compromising catalytic geometry

The α6–α7 helical platform constitutes the structural core of SynGAP that is needed to stabilize Ras/Rap-Switches I and II based on the modelling (Fig. 5). Helix α6 acts as a central scaffold for the catalytic site, supporting the Arg-finger loop and anchoring both Switch regions through a coordinated H-bond network and hydrophobic packing (Fig. 5B,E). Helix α6 missense variants (e.g., R596H, P600V, S604L; Table 1) consistently weaken binding affinities and compromise the required catalytic geometries in the simulations. Similarly, helix α7 primarily secures the flexible Switch I loop via conserved electrostatic contacts (Fig. 5C,F). FoldX-MD/Rosetta interaction energy calculations predicted that several missense variants (R621Q, A627V, K628N, N635S, D644A; Table 1) would profoundly perturb this network and reduce the Switch I stability.

A set of variants was selected for in-depth MD analysis to study the missense effects on the key helical platform interactions:

• **R596H** alters a strictly conserved residue that, while having minimal impact on Ras binding affinity in GAPs, is known to be essential for preserving catalytic GTPase function by stabilizing the Arg-finger loop and promoting optimal positioning of Arg485 (Wang et al., 2013; Wang et al., 2012; Sermon et al., 1998). In the WT-SynGAP–Ras/Rap complex simulations, Arg596 extends from helix α6 to form a stable H-bonding network with the Arg-finger loop (Fig. S13), rigidifying the loop itself and assisting the optimal positioning of Arg485 for catalysis. The Arg-finger loop further stabilizes Switch II and helps position the catalytic glutamine. The R596H mutation disrupts this architecture, as histidine side-chain fails to maintain a similar network in both complexes in the simulations (Figs. S13-S14). This loss of scaffolding destabilizes the positioning of the Arg-finger and Switch II causing poor catalytic water coordination (Fig. S6, Table 2), providing a plausible structural explanation for the reported decrease in catalytic activity (Wang et al., 2013; Wang et al., 2012; Sermon et al., 1998).
• **P600V** impairs SynGAP-mediated Rap-GTPase activity while sparing Ras activity with *GAP1*^*IP4B*^ based on in vitro experiments (Kupzig et al., 2009). In the WT-SynGAP–Ras/Rap simulations, Pro600 stacks against residue 64 of Switch II (Ras-Tyr64; Rap-Phe64; Fig. S18A–B, D–E). The P600V mutation is structurally accommodated in the SynGAP–Ras complex model, as Ras-Tyr64 maintains stable stacking with the introduced valine (Figs. S18B and S19). Conversely, the unique Rap-Switch II conformation in the model positions Rap-Phe64 a lot closer to helix α6 causing steric clashing with the bulkier valine side-chain (Figs. S18E and S19). This steric conflict displaces Rap-Switch II away from the interface in the simulations, providing an atomistic rationale for this selective loss of Rap-GTPase activity with P600V in vitro.
• **S604L** disrupts a highly conserved residue among dual-specificity GAPs (Fig. 3B) that serves as a key determinant of Rap-Switch II stabilization based on modelling. In the WT-SynGAP–Ras/Rap simulations, Ser604 frequently H-bonds with the Switch II backbone residues (Figs. 5B,E and S11). With the S604L variant, the leucine packs hydrophobically against Ras-Switch II, potentially rigidifying the complex interface (Fig. 5B). This is supported by the predicted interface stabilization (Rosetta: ΔΔG = −0.53 kcal/mol) and consistent with the notion that helix α6 stabilizes SynGAP–Ras binding primarily via hydrophobic interactions (Scheffzek et al., 1997). Regardless of this apparent stability, S604L does end up disrupting the native α6–Switch II interaction network and local alignment, with catalytic-water coordination subsequently lost in all three SynGAP–Ras simulation replicas after ∼200 ns (Figs. 5B and S6). Meanwhile, the modelling suggests that the Rap-GTPase activity requires polar stabilization of the “open” Switch II. With the SynGAP–Rap complex, S604L mutation eliminates this essential H-bond, destabilizing Switch II and misplacing the catalytic Gln63 at the active-site (Fig. 5E). This predicted destabilization, which is picked up by both binding energy protocols (FoldX-MD: ΔΔG = 1.4 kcal/mol; Rosetta: ΔΔG = 1.6 kcal/mol), mirrors the catalytic impairment observed for the analogous Plexin-Asn1009 mutations (Wang et al., 2013).
• **K628N** is predicted to eliminate a critical electrostatic interaction based on the modelling, as Lys628 normally forms salt-bridges with Glu37 or Asp38 in both Ras- and Rap-GTPases (Fig. 5C), resulting in destabilization of the interface and increased solvent exposure of Switch I and II regions.

These in silico mutational analyses suggest that several of the established α6–α7 helical platform missense variants could potentially disrupt SynGAP–GTPase regulation by destabilizing Switch I/II positioning and compromising the catalytic geometry, thereby weakening both complex stability and GAP-mediated hydrolysis.

#### Mutating the arginine finger can abolish GTPase activity, yet complex formation can persist

Because Arg485 primarily stabilizes the GTP transition state during hydrolysis, mutating the Arg-finger has been well-established to weaken or even abolish GTPase activity altogether in vitro (Pena et al., 2008; Sermon et al., 1998; Ahmadian et al., 1997; Ahmadian et al., 2003). It is, however, less clear how missense mutations directly targeting the Arg-finger affect SynGAP–GTPase complex formation or binding affinity. The WT-SynGAP–GTPase simulations suggest that Arg485 not only stabilizes the transition state but also contributes to maintaining the structural integrity of the broader Arg-finger loop. Specifically, Arg485 forms a stabilizing H-bond with the His481 backbone, rigidifying the loop to ensure its precise positioning within the GTPase active-site (Figs. S20A–B and S21).

To investigate these non-catalytic contributions, three engineered/artificial Arg-finger missense variants from prior studies (Pena et al., 2008; Araki et al., 2024), were probed in depth using MD simulations:

• **R485P**, which removes completely the SynGAP-mediated Ras/Rap GTP hydrolysis in vitro (Pena et al., 2008), is clearly detrimental also for the complex interface organization with both GTPases based on the simulations. The introduced proline induces a collapse of the Arg-finger loop, as it lacks both the charged/bulky side-chain and the free backbone amide required to maintain structural integrity. This disruption breaks critical Switch I interactions, likely preventing formation of a catalytically competent active-site geometry (Fig. S20A–B).
• **F484A/R485L** has been shown to drastically reduce SynGAP–Ras GTPase activity, yet the double mutation does not induce the SRD phenotype in mice, suggesting preservation of a separate “structural” role (Araki et al., 2024). Whether SynGAP–GTPase complex formation is required for this role remains unclear. If complexing is indeed essential, Rosetta predicts a stabilizing effect of the double mutation on the SynGAP–Ras complex (${\mathrm{\Delta \Delta G}}_{\mathrm{bind}}$ = −0.6 kcal/mol), with MD simulations revealing a compensatory interaction in which Leu485 packs against Ras-Tyr32 (Fig. S20A). This effect is absent in the modelled double mutant–Rap complex, where Leu485 cannot access the “open” Tyr32 conformation, resulting in only a negligible change in the calculated affinity (Figs. S20B).
• **R485K** strongly diminishes SynGAP-induced Rap-GTPase activity while only partially affecting its Ras activity according to Pena et al. (Pena et al., 2008), whereas, at the interface-level, only negligible differences between the GTPases were found based on the binding affinity calculations (Table 1). The WT-SynGAP–Rap complex simulations reveal that Arg485 forms a robust H-bond with the backbone of Rap-Lys31, stabilizing the “open” conformation of the adjacent Tyr32 that is essential for the Rap-GTPase catalysis (Figs. S20B). Besides, the bulky guanidinium group of Arg485 may sterically induce Rap-Tyr32 conformational transition to an open state, whereas the smaller lysine side-chain in R485K may be insufficient to drive or stabilize the open state, explaining the loss of Rap-specific catalytic efficiency.

In general, substituting the Arg-finger in RasGAPs drastically reduces Ras-GTPase activity while exerting minimal effects on complex formation affinity; in some cases, such mutations even increase binding by “locking” the non-reactive complex in the ground state (Sermon et al., 1998; Ahmadian et al., 1997; Ahmadian et al., 2003). Likewise, the modelling indicates that Arg485-targeting missense variants retain a structurally intact SynGAP–GTPase complex. Notably, this includes the F484 A/R485L double mutant (Figs. S20–S21), whose predicted interaction affinity was even improved in comparison to the WT system, and, which, importantly, has been used in vivo to demonstrate that SynGAP's catalytic activity would be decoupled from the behavioral SRD phenotype (Araki et al., 2024). While this double mutation attenuates catalytic activity toward Ras (its effect on Rap remains untested), some residual activity may persist; moreover, whether stable complex formation between SynGAP and its effector GTPases is required for normal non-SRD synaptic function remains unknown. Here, missense variants that interfere with SynGAP's interaction with the membrane-tethered GTPases could therefore compromise its structural role at the synapse, in ways that simple catalytic substitutions do not.

#### Mutations in the arginine finger loop disrupt the active-site preorganization

In-depth analysis of established cancer and SRD missense variants, combined with binding affinity calculations and WT-SynGAP–Ras/Rap MD simulations, identified a network of Arg-finger loop residues (e.g., His481, Glu486, Asn487, Lys492) that likely are needed stabilize the Ras/Rap-Switch I region and the catalytic centre. These in silico findings suggest that, beyond the Arg-finger itself, adjacent loop residues are also likely critical for GAP activity and could contribute to GTPase binding.

Three missense variants were selected for in-depth MD analysis to assess their effects on these key interactions:

• **H481D** disrupts the π-stacking interactions formed by the His481 side-chain with Tyr32 in the WT-SynGAP–GTPase complexes based on the modelling. In the WT simulations, His481 stabilizes Switch I via π–π interactions with Tyr32, while adopting an edge-to-face orientation in Ras and a parallel stack in Rap. In the SynGAP–Ras complex simulations, His481 also forms a very stable stabilizing H-bond with Asp30. Histidine substitution with aspartate introduces an anionic side-chain that abolishes both the π-stacking and the Ras-specific H-bond, thereby destabilizing the GAP–Switch I interface in both complexes (Figs. S18C, F and S19).
• **N487K/T** destabilizes the Arg-finger loop and catalytic center based on the modelling. In the WT simulations, Asn487 provides dual stabilization by interacting with Switch II to help position the catalytic glutamine, while also stabilizing the Arg-finger loop through interaction with Arg596, a key anchoring residue (Figs. 6B,E and S12). The N487K/T substitutions are predicted to disrupt these interactions, destabilizing the SynGAP-mediated catalytic environment for both Ras and Rap (Figs. 6B,E and S13). Despite the 20-fold reduction in the Rap-GTPase activity for the N487T variant reported in vitro (Pena et al., 2008), binding affinity predictions indicate a near-neutral effect on complex formation (Table 1). This discrepancy likely reflects limitations of the WT-SynGAP–Rap model, which captures a relatively loose conformation in which Asn487 interacts directly with the Gln63 side-chain (Fig. 6E). In contrast, within the SynGAP–Ras complex, Asn487 engages the Gly60 backbone, effectively anchoring Gln61 in its catalytic orientation during the first half of the simulations, before the pre-reaction state starts to gradually relax (Fig. 6B). By analogy, Asn487 in Rap is proposed to coordinate a backbone atom within Switch II, thereby more effectively constraining Gln63 in a catalytically competent orientation during the pre-reaction state. This context provides a plausible explanation for both the increased mobility of Rap-Gln63 relative to Ras-Gln61 observed in WT simulations (Fig. 4A-B) and the underestimation of the functional importance of this residue in the calculations.
• **K492E** disrupts Switch II and catalytic glutamine positioning based on the modelling. Lys492 acts as a peripheral anchor, stabilizing Switch II and the catalytic glutamine via a salt-bridge with acidic residues on the GTPase (Ras-Glu63; Rap-Glu62). In the charge-reversal K492E variant, the introduced glutamate side-chain is repelled in the simulations from the interface, destabilizing the catalytic glutamine in both complexes and disrupting Switch II α-helical structure in the SynGAP–Ras complex (Figs. S13 and S14).

These variant-GTPase complex simulations highlight the notion that integrity of the Arg-finger loop, beyond the Arg-finger residue itself, is essential for both RasGAP and RapGAP activity, reinforcing its role as a shared structural determinant of SynGAP's dual specificity. Although GTP was not included in the complex-interface stability scoring in this study, it is expected to provide additional stabilizing contributions.

#### The C2 domain missense variants: Weakening SynGAP–GTPase complex formation?

Several SynGAP missense variants within the C2 domain were predicted to destabilize the SynGAP–GTPase interface (e.g., R272W, E360K, R401Q/W; Table 1), mainly by perturbing electrostatic contacts that are firmly established and upkept in the WT simulations.

Focusing on Arg401, the positively charged residue forms a critical inter-protein salt-bridge with Ras/Rap-Asp69, serving as the primary anchor for the C2–GTPase association (Figs. 6C,F and S12). The R401Q variant simulations indicated that the shorter glutamine side-chain fails to connect with Asp69. In the SynGAP–Ras complex, this loss of anchoring directly weakens the interface in the simulations, providing a structural basis for the reduction in activity also observed in vitro (Paul et al., 2025a). However, the modelling also suggests that Arg401 plays a more crucial role in the SynGAP–Rap complex by stabilizing the “open” loop conformation of Rap-Switch II necessary for the GTP hydrolysis. In fact, the R401Q mutation could disrupt the essential helix-to-loop transition of Rap-Switch II (Fig. 6F) and this inability to induce or stabilize the catalytically competent state would explain why mutating Arg401 in vitro completely abolishes SynGAP-mediated Rap-GTPase activity while only weakening the Ras activity (Paul et al., 2025a).

Furthermore, missense variants located near the putative SynGAP–membrane interface, particularly in the C2 domain (Fig. 1A) (Ali et al., 2024), may impair membrane binding and thereby indirectly reduce SynGAP interaction with native membrane-tethered GTPases (Ali et al., 2024; Grewal et al., 2011). Notably, the in vitro work on SynGAP–GTPase activity and association have so far lacked the membrane component, and, accordingly, this hypothesis will require validation in membrane-based experimental systems in the future.

#### SynGAP missense variants as putative Ras/Rap-GTPase specificity modifiers

Based on SynGAP–GTPase complex modelling, particularly the complex-affinity calculations, two missense variants emerged as putative modifiers of SynGAP interactions with Ras and Rap, exhibiting differential effects on the two GTPases.

• **E486K** preferentially perturbed the SynGAP–Ras interface based on the modelling. In the WT-SynGAP–Ras complex simulations, Glu486 participates in a robust electrostatic network involving Arg479, Ras-Asn86, and Ras-Lys88. Charge reversal in E486K introduces a severe electrostatic clash with Ras-Lys88 and Arg475, disrupting this network. The disruption was already apparent by 100 ns and became more pronounced by 500 ns, markedly destabilizing the modelled interface. In contrast, the WT complex model retained an overall intact interaction network at 500 ns despite signs of gradual weakening (Figs. S15A and S15B). The mutation appeared to be better tolerated in the SynGAP–Rap complex, where the equivalent residue is the neutral Rap-Ser88, reducing electrostatic repulsion. Nevertheless, E486K progressively perturbed the SynGAP–Rap interface as well, with disruption becoming more evident by ∼500 ns (Figs. S15D and S17; Table 1).
• **R479Q** preferentially disrupted the SynGAP–Rap interface based on the modelling. In WT-SynGAP–Rap simulations, Arg479 serves as a key intermolecular anchor by forming a stable salt-bridge with Rap-Glu123 throughout the 500-ns simulations. The R479Q substitution abolishes this ionic interaction, substantially weakening SynGAP–Rap affinity (Fig. S15D). In the SynGAP–Ras complex simulations, Arg479 interacts primarily with SynGAP-Glu486 rather than acting as a direct intermolecular anchor, resulting in a comparatively smaller effect on interface stability. However, loss of this local interaction may still perturb the Arg-finger-loop environment and associated catalytic network, consistent with the reduced catalytic-water coordination observed in the SynGAP–Ras simulations (Table 2; Fig. S6).

In short, E486K and R479Q exhibit distinct Ras- and Rap-dependent effects and may therefore modulate the GTPase selectivity of SynGAP. Affinity calculations suggest that E486K is more detrimental to Ras binding, whereas R479Q more strongly impairs Rap binding. However, MD simulations suggest that both variants would affect the catalytic-site organization through indirect structural perturbations, even when complex formation is preserved. Experimental validation will be required to determine whether these predicted interaction biases translate into altered GTPase specificity.

### Differential GTP binding dynamics behind higher potency for Rap1b over Ras?

SynGAP deactivates Rap1b in vitro ∼100 times more potently than Ras, despite being a RasGAP based on homology (Pena et al., 2008; Krapivinsky et al., 2004; Walkup et al., 2015). The WT-SynGAP–Ras/Rap simulations provide a plausible structural rationale for this massive difference. While protein–protein binding energies from MM/GBSA calculations are at a comparable level for the SynGAP–Ras and SynGAP–Rap complexes (Table S4; Fig. S7), GTP itself is predicted to bind with substantially higher affinity within the SynGAP-activated Rap1b (ΔG = −283.3 ± 4.4 kcal/mol) than within its Ras counterpart (ΔG = −231.3 ± 16.4 kcal/mol; Table 3; Fig. 7A-B). Based on the calculations, this divergence is driven by more favorable electrostatic contributions with the former complex (SynGAP–Rap: −1100.8 ± 29 kcal/mol vs. SynGAP–Ras: −880.9 ± 67.0 kcal/mol).

**Table 3:** Binding affinity and stability of GTP within SynGAP–Ras and SynGAP–Rap GTPase complexes.

**Variant**	**SynGAP–Ras Complex**				**SynGAP–Rap Complex**			
	**MM/GBSA**$\;{\mathrm{\Delta G}}_{\mathrm{bind}}$^1^	**MM/GBSA**$\Delta {\mathrm{\Delta G}}_{\mathrm{bind}}$^2^	**RMSF**^3^	**RMSD**^4^	**MM/GBSA**$\;{\mathrm{\Delta G}}_{\mathrm{bind}}$^1^	**MM/GBSA**$\Delta {\mathrm{\Delta G}}_{\mathrm{bind}}$^2^	**RMSF**^3^	**RMSD**^4^
WT	-231.3 ± 16.4	N/A	2.4 ± 0.6	0.49 ± 0.08	-283.3 ± 4.4	N/A	3.1 ± 0.2	0.61 ± 0.07
R401Q†	-236.2 ± 14.8	-4.9	3.2 ± 0.8	**0.75 ± 0.13**	-269.9 ± 12.9	13.4	2.9 ± 0.2	0.66 ± 0.13
R479Q†	-232.0 ± 12.2	-0.7	2.8 ± 0.9	**0.65 ± 0.06**	-289.2 ± 9.6	-5.9	2.6 ± 0.5	0.73 ± 0.08
H481D	**-279.4 ± 16.5**	**-48.1**	2.6 ± 0.7	0.66 ± 0.20	-281.9 ± 5.5	1.4	2.5 ± 0.8	0.66 ± 0.18
R485L-F484A	-204.9 ± 29.4	26.4	**3.9 ± 0.2**	**0.81 ± 0.06**	**-226.0 ± 4.2**	**57.3**	3 ± 0.5	**0.75 ± 0.05**
R485K	**-262.3 ± 13.6**	**-31**	**3.6 ± 0.2**	0.75 ± 0.19	**-260.1 ± 12.5**	**23.2**	2.6 ± 0.5	0.71 ± 0.08
R485P	-210.8 ± 5.3	20.5	**4.2 ± 1**	**0.80 ± 0.07**	**-217.2 ± 7.8**	**66.1**	2.9 ± 0.3	**0.78 ± 0.06**
E486K†	**-279.8 ± 6.5**	**-48.5**	2.8 ± 0.5	0.68 ± 0.15	-278.7 ± 6.4	4.6	**2.5 ± 0.3**	0.56 ± 0.17
N487K	-248.4 ± 23.7	-17.1	2.8 ± 0.5	0.59 ± 0.08	-289.8 ± 5.6	-6.5	**2 ± 0.4**	0.54 ± 0.10
N487T	-228.3 ± 12.4	3	2.2 ± 0.9	0.51 ± 0.14	-283.9 ± 7.1	-0.6	**2.4 ± 0.4**	0.83 ± 0.08
K492E†	-236.0 ± 25.0	-4.7	2.3 ± 0.8	0.63 ± 0.22	-283.7 ± 5.5	-0.4	2.5 ± 0.4	0.63 ± 0.15
R596H†	-251.1 ± 32.9	-19.8	2.8 ± 0.9	0.58 ± 0.05	-288.9 ± 6.3	-5.6	2.6 ± 0.6	0.67 ± 0.06
P600V	-249.2 ± 22.1	-17.9	2.3 ± 1.2	0.57 ± 0.07	-277.2 ± 7.4	6.1	2.3 ± 0.8	**0.82 ± 0.05**
S604L†	-221.8 ± 15.2	9.5	2.5 ± 1.1	0.57 ± 0.17	-281.9 ± 7.6	1.4	**1.7 ± 0**	0.65 ± 0.16
K628N	-210.3 ± 11.1	21	2 ± 0.6	0.61 ± 0.18	-290.3 ± 5.8	-7	**1.8 ± 0.3**	0.80 ± 0.21

Binding free energies and structural fluctuation metrics computed for each frame from 500-ns simulations for the GTP molecule in the WT and missense variant SynGAP–Ras and SynGAP–Rap complexes. ΔG and ΔΔG values are reported in kcal/mol while RMSF (root-mean-square fluctuation) and RMSD (root-mean-square deviation) in Å. For RMSF and RMSD, positive values indicate increased fluctuations or structural movement whereas negative value indicate substrate rigidification. While for MM/GBSA a positive ΔΔG reflects decreased binding affinity and a negative ΔΔG reflects increased affinity. Values are reported as the mean across three replica simulations ± the standard deviation between replicas. Values are highlighted in bold when the variant mean ± SD falls outside the WT mean ± SD for that metric, with WT and variant statistics computed from three independent simulation replicas. N/A stands for "not available".

1 MM/GBSA $\;{\mathrm{\Delta G}}_{\mathrm{bind}}\;$binding free energies of GTP were computed using AMBER *MMPBSA.py*, with averages reported across three replica simulations.

2 The reported value represents ${\mathrm{\Delta \Delta G}}_{\mathrm{bind}}\;$calculated as ${\mathrm{\Delta G}}_{\mathrm{bind}-\mathrm{WT}}$**-**${\mathrm{\Delta G}}_{\mathrm{bind}-\mathrm{variant}.}$

3 RMSF of GTP heavy atoms was averaged across three replica simulations.

4 RMSD of GTP heavy atoms relative to the starting structure was averaged across three replica simulations.

† Missense variants correspond to ClinVar-annotated substitutions, classified as VUS (R401Q, E486K, K492E, S604L) or as likely benign (R479Q, R596H, R621Q).

**Fig. 7:**
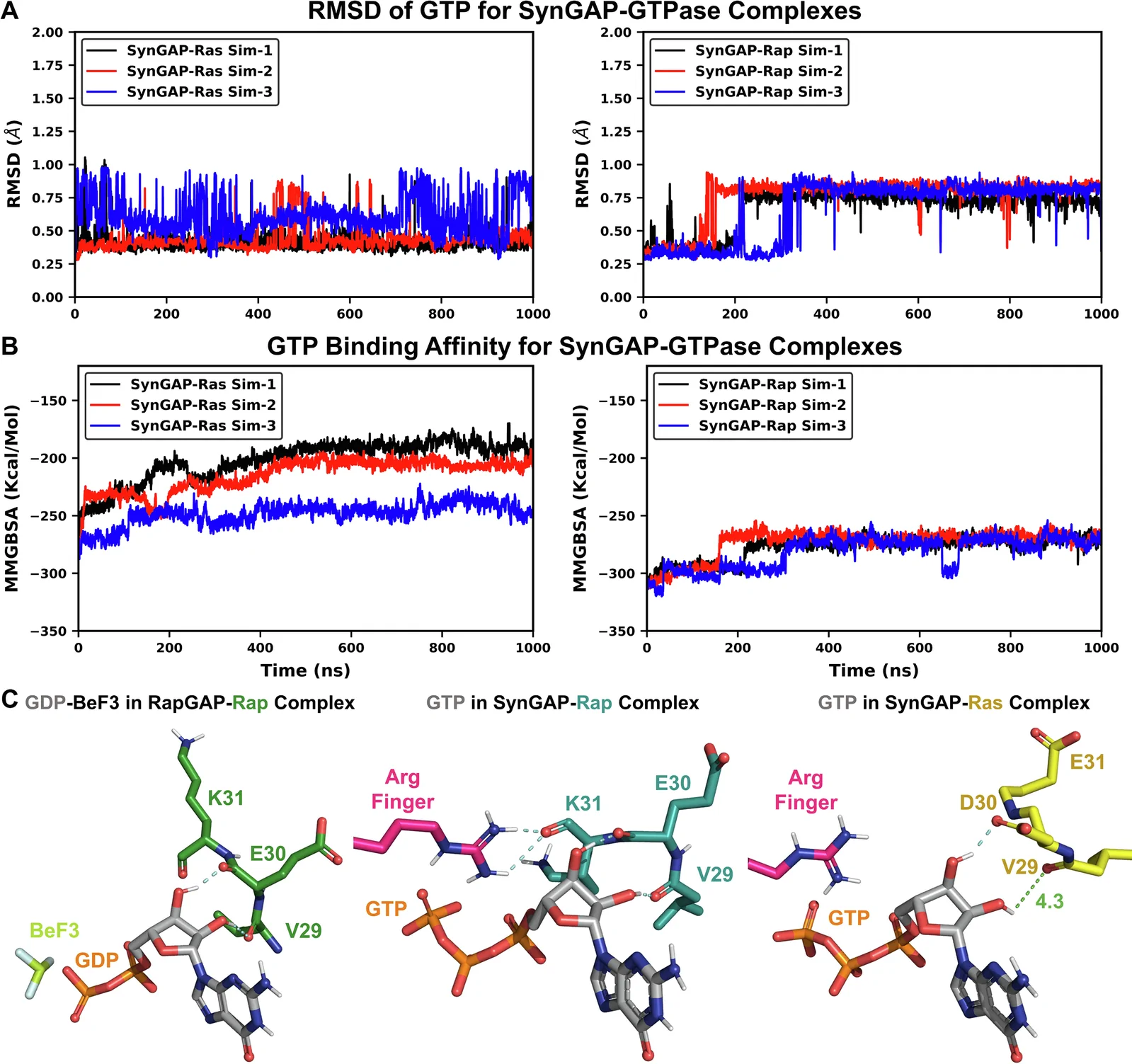
**Stability, substrate binding energy, and active-site structural preorganization of SynGAP–GTPase complexes. (A)** Root-mean-square deviation (RMSD) calculations track the positional stability of GTP heavy atoms over 1 μs of MD simulation across three independent replicas (Black: Sim-1, Red: Sim-2, Blue: Sim-3) for SynGAP–Ras (left) and SynGAP–Rap (right) complexes. In SynGAP–Ras, GTP maintains a baseline (∼0.4 Å) with frequent transient fluctuations to 0.5-1 Å mainly seen in the third simulation. In SynGAP–Rap, all replicas undergo a step-wise transition to a stable plateau at ∼0.8 Å with reduced high-frequency noise. **(B)** Time-series plots show the evolution of MM/GBSA binding free energy for GTP within the active-sites of SynGAP–Ras (left) and SynGAP–Rap (right) complexes over 1 μs simulation time. Replicas of the SynGAP–Rap simulation display consistently more favorable binding free energy (approximately –270 to –300 kcal/mol) with tightly clustered trajectories compared to SynGAP–Ras (–180 to –270 kcal/mol), demonstrating stronger substrate stabilization in the SynGAP*–*Rap complex. **(C)** The RapGAP–Rap1b X-ray crystal structure (left; PDB: 3BRW (Scrima et al., 2008)) shows that the Glu30 backbone carbonyl H-bonds with the 3′-hydroxyl, while the Val29 backbone engages the 2′-hydroxyl. A representative MD snapshot of the SynGAP–Rap1b complex (middle) shows that this Glu30/Val29 backbone coordination of the ribose locks the Lys31 backbone into a stable electrostatic interaction with the SynGAP-Arg-finger, enforcing catalytic preorganization. In contrast, a SynGAP–Ras simulation snapshot (right) shows the Asp30 side-chain forming dynamic, less stable H-bonds with the GTP ribose, failing to rigidly anchor the nucleotide compared with the Rap1b motif. All plotted curves are displayed using a 50-frame (0.5 ns) moving average for visual clarity. (For interpretation of the references to colour in this figure legend, the reader is referred to the web version of this article.)

At the structural level, this difference could stem from the Glu30-Lys31 motif of Rap1b, which is a known specificity determinant for effector interactions within the wider Ras-GTPase family (Devanand et al., 2018; Zhang et al., 2014; Sprang, 1995). In the SynGAP–Rap simulations, the Rap-Glu30 backbone carbonyl forms a stable H-bond with the 3′-hydroxyl of the GTP ribose, positioning Rap-Val29 to form a secondary H-bond with the 2′-hydroxyl of GTP, while also locking Rap-Lys31 into a direct electrostatic interaction with SynGAP's Arg-finger. These modelled interactions optimally anchor the guanine-ribose core without over-stabilizing the substrate's cleavable phosphate, enforcing rigid pre-organization of the active-site. The coordination of the Val29-Glu30 Rap segment observed in the SynGAP–Rap simulations mirrors interactions reported in the substrate-bound Rap1 X-ray crystal structures (Scrima et al., 2008; Zhang et al., 2014), supporting the biological relevance of the modelled active-site. Conversely, the equivalent interaction in the SynGAP–Ras complex simulations is mediated by the Asp30-Glu31 pair, of which Asp30 side-chain fluctuates dynamically between ribose hydroxyls (Fig. 7C).

This distinction in GTP binding between Ras and Rap was most evident during the early stage of the simulations (0-100 ns), when the SynGAP−Rap1b complex displayed low GTP mobility (RMSD = 0.36 ± 0.02 Å; normalized B-factors = 1.5 ± 0.2; Table S5). With extended sampling (100-500 ns), GTP in the WT complex underwent a stepwise positional shift, resulting in an approximately twofold increase in coordinate displacement (RMSD = 0.61 ± 0.07 Å). This transition occurred between ∼180 and 300 ns, depending on the simulation replica (Fig. 7A). The increase in GTP mobility coincided with weakening of the initial Switch I-GTP interaction network and is therefore consistent with the gradual relaxation of the fully competent pre-reaction state (Section 0.). Notably, several missense variants, including P600V and S604L, exhibited comparable GTP displacement already within the first 100 ns of simulation (Table S5), suggesting that these substitutions may accelerate disruption of the proposed pre-catalytic arrangement. However, because GTP in the WT complex also progressively diverged from the starting structure at longer timescales, RMSD and RMSF should be seen as less sensitive measures for distinguishing deleterious variant effects (Table 3).

In contrast, GTP bound in WT-SynGAP–Ras model was already more mobile during the initial 100 ns (RMSD = 0.53 ± 0.15 Å), making moderate variant effects harder to distinguish from WT fluctuations, except for some Arg-finger substitutions (Table S5). Several SynGAPvariants with Ras, particularly H481D, E486K, and R485K, nevertheless showed increased GTP affinity at 500 ns (Table 3), while catalytic-water coordination varied markedly between simulation replicas, ranging from highly preserved to severely disrupted (Table S3; Fig. S6). This suggests compensatory interactions that stabilize the GTP-bound state without necessarily preserving a uniformly competent catalytic arrangement. Notably, although R485K has previously been shown in vitro to retain partial RasGAP activity (Pena et al., 2008), it remains to be determined whether the stronger estimated GTP binding affinity observed for R485K reflects compensatory stabilization or altered catalytic efficiency.

## Conclusions

This study establishes a structural framework for understanding the dual-Ras/Rap1b-GTPase regulation by SynGAP using a multitude of in silico approaches. The molecular modelling proposes that SynGAP employs a canonical Arg-finger and Ras-Gln61 mechanism for the deactivation of Ras (Fig. 2B), but switches to a non-canonical, Plexin-like mode for Rap1b (Fig. 2C). A key discovery is the identification of regulatory SynGAP activation segment (residues 628–645), which, according to modelling, must adopt an “open” conformation to allow Rap binding (Fig. 3E). The modelling also indicates that missense mutations could potentially impair the SynGAP-mediated GTP hydrolysis (Fig. 1B) through several different ways (Fig. 8), including the destabilization of key catalytic residues or the disruption anchoring interactions at the SynGAP–Ras/Rap-Switch I/II interfaces. The study also provides plausible explanation how certain missense mutations (e.g., P600V) could selectively disrupt Rap but not Ras activation and highlights the essential roles for the GAP domain residue Ser604 and the C2 domain residue Arg401 in stabilizing the open state of Rap-Switch II. Additionally, the favorable pre-organization of the SynGAP-bound Rap active-site is proposed to lead into significantly higher GTP affinity based on the free energy calculations in the pre-reaction state, providing a structural rationale for the prior in vitro results in which SynGAP was measured to have 100-fold greater potency toward Rap1b than Ras (Pena et al., 2008; Walkup et al., 2015). Finally, the substantial overlap between cancer- and ClinVar-derived missense mutations (Fig. 1E) suggests indirectly that impaired GTPase function or at least binding could contribute to some SRD phenotypes. Overall, FoldX-MD/Rosetta calculations predicted 33 missense variants to destabilize the complex and, thus, potentially hinder its formation, including eight ClinVar variants and 18 cancer-associated variants.

**Fig. 8:**
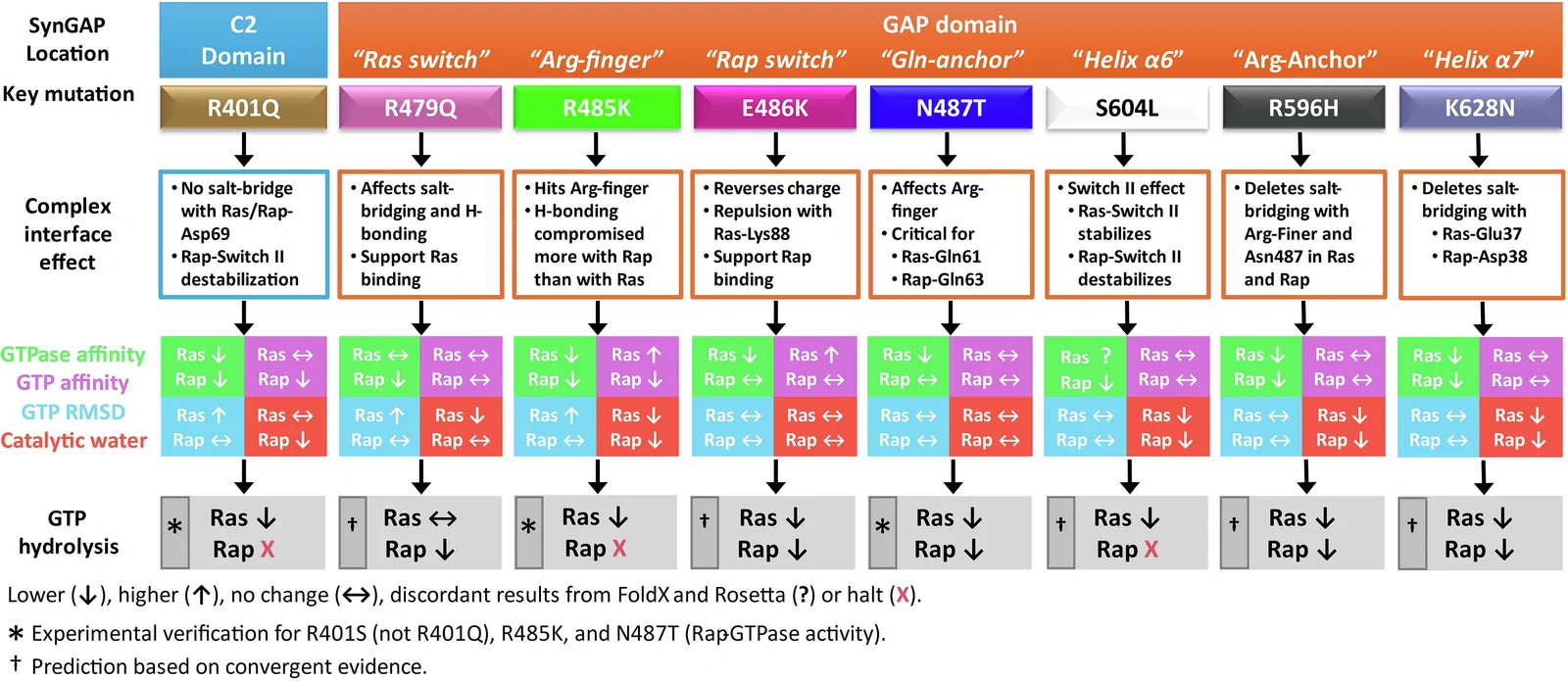
**Summary of potential SynGAP missense mutation effects on the Ras/Rap-GTPase binding specificity and catalytic activity based on molecular modelling.** The effects of key missense variants, including R401Q (Paul et al., 2025a) (Fig. 6C,F), R479Q (Figs. S15B,D and S16), E486K (Figs. S15B,D and S17), N487T (Pena et al., 2008) (Figs. 6B,E and S12), S604L (Fig. 5B,E), R596H (Figs. S13 and S14), and K628N (Fig. 5C,F), on the SynGAP–Ras/Rap-GTPase interactions are summarized to illustrate the dual activation mechanism. Arrows indicate missense mutation-induced changes relative to the WT systems across four metrics: SynGAP–GTPase binding affinity (FoldX-MD and Rosetta $\;{\mathrm{\Delta \Delta G}}_{\mathrm{bind}}$), GTP binding affinity (MM/GBSA$\;\;{\mathrm{\Delta \Delta G}}_{\mathrm{bind}}$), GTP positional stability (RMSD of GTP heavy atoms), and catalytic-water coordination frequency. Upward arrows denote increased affinity, mobility, or water coordination, whereas downward arrows indicate decreases. Convergent effects are assigned when any of the evaluated metrics indicates an unfavorable change relative to WT; for example, any decrease in affinity or catalytic-water coordination results in a downward assignment for overall catalytic impact. The S604L missense mutation is estimated to not only decrease GTP binding but be disastrous for the Rap-GTPase GTP hydrolysis (see red X) due to its role in Switch II stabilization. Notably, experimental validation is essential for making any definitive determinations on any of these predicted effects of missense mutations on the GTPase binding or activity. For cross-reference, the full values and descriptions of the metrics used in the summary are provided in Table 1, Table 2, Table 3. (For interpretation of the references to colour in this figure legend, the reader is referred to the web version of this article.)

## Key highlights

1. **Plexin-like Rap Activation Mechanism of SynGAP.** The study provides the first structural model for SynGAP employing a “Plexin-like” non-canonical catalysis deactivation mechanism for Rap1b, utilizing an “Arg-finger” (Arg485) and Rap-Gln63, rather than the “Asn-thumb” used by canonical RapGAPs.
2. **Identification of the “Activation Segment” in SynGAP.** Modelling identifies a potential regulatory segment (res. 628–645) that acts as a gatekeeper, determining the accessibility of the catalytic pocket for GTPase binding. This SynGAP segment must undergo a conformational shift to an “open” state to resolve steric clashes allowing Rap binding.
3. **Mapping of SynGAP Missense Variants Disrupting Catalytic Ras/Rap Activity.** The modelling maps missense variants that potentially impair GTPase activity by either destabilizing key catalytic residues (SynGAP-Arg485, Ras-Gln61/Rap-Gln63) or disrupting the protein-protein interface at Switch I/II binding interfaces.
4. **Potential Role of Ser604 in Maintaining SynGAP–Rap Complex in a Catalytical State.** MD simulations suggest that Ser604, a conserved residue in the dual GAPs, may play a critical role in stabilizing the Rap-Switch II open conformation, role potentially analogous to the function of Asn1009 in Plexin-GAPs.
5. **Atomistic Rationale for Differential C2 Domain Role in Ras and Rap Activity.** The C2 domain facilitates full catalytic activity in both complexes by stabilizing α3-helix and Switch II of GTPases. However, it is essential for Rap deactivation because Arg401 anchors the Rap-Switch II “open” loop conformation via a critical salt-bridge with Asp69, providing a structural requirement for catalysis that is absent in the SynGAP–Ras complex.
6. **Structural Rationale for SynGAP's Higher Potency toward Rap1b than Ras.** SynGAP's 100-fold higher potency for Rap1b is driven by the pre-organization of the catalytic site. A specific Val29-Glu30-Lys31 motif in Rap1b creates a superior electrostatic stabilization that optimally anchors the GTP ribose core, resulting in significantly lower substrate mobility and fluctuations compared to Ras active-site.
7. **Predicted Specificity Modifiers for Ras/Rap Selectivity.** The study identifies E486K and R479Q as potential variants that differentially modulate GTPase affinity. E486K is predicted to selectively destabilize the Ras interface via Lys88 repulsion, while R479Q selectively impairs Rap affinity via loss of salt-bridging with Rap-Glu123.
8. **Overlap Between SynGAP-Related Disorder and Somatic Cancer Variants**. 60 of SRD-associated missense variants may impair SynGAP's ability to activate or complex with GTPases, suggesting that GTPase dysregulation may be a contributing factor to the clinical phenotype in some patients. Also, variants of homologous sites in other RasGAPs (e.g., Arg596) are implicated in cancer, highlighting that such mutations could also contribute to oncogenic processes.

## CRediT authorship contribution statement

**Aliaa E. Ali:** Writing – review & editing, Writing – original draft, Validation, Methodology, Formal analysis, Visualization, Data curation, Investigation.. **Arto Liljeblad:** Writing – review & editing, Writing – original draft, Investigation. **Jukka V. Lehtonen:** Writing – review & editing, Visualization, Data curation. **Noboru H. Komiyama:** Writing – review & editing. **Pekka A. Postila:** Writing – review & editing, Writing – original draft, Visualization, Validation, Supervision, Project administration, Methodology, Investigation, Funding acquisition, Formal analysis, Conceptualization.

## Declaration of competing interest

The authors declare that they have no known competing financial interests or personal relationships that could have appeared to influence the work reported in this paper.

## Data Availability

Data will be made available on request.
