# Supplementary material for: Investigating the effects of missense mutations on the dual Ras/Rap activation mechanism of SYNGAP1 using molecular modelling
Source: J Struct Biol X. 2026 Sep 10;14:100162. doi: 10.1016/j.yjsbx.2026.100162 (PMC13634398; doi:10.1016/j.yjsbx.2026.100162)
Supplement: Supplementary file 1 [file mmc1.pdf]

Supplementary Information

for

Investigating the Effects of Missense Mutations on the Dual Ras/Rap Activation Mechanism of SYNGAP1 Using Molecular Modelling

Aliaa E. Ali [1,2], Arto Liljeblad [1,2], Jukka V. Lehtonen [3,4], Noboru H. Komiyama [5,6,7], Pekka A. Postila [1,2,*]

[1] Institute of Biomedicine, Integrative Physiology and Pharmacology, University of Turku, FI-20014 Turku, Finland.

[2] InFLAMES Research Flagship, University of Turku, 20014 Turku, Finland.

[3] Structural Bioinformatics Laboratory, Department of Natural and Health Sciences, Åbo Akademi University, FI-20500 Turku, Finland.

[4] InFLAMES Research Flagship, Åbo Akademi University, FI-20500 Turku, Finland.

[5] Institute for Neuroscience and Cardiovascular Research, University of Edinburgh, Edinburgh EH16 4SB, UK.

[6] Simons Initiative for the Developing Brain (SIDB), Centre for Discovery Brain Sciences, University of Edinburgh, Edinburgh EH8 9XD, UK.

[7] The Patrick Wild Centre, Centre for Discovery Brain Sciences, University of Edinburgh, Edinburgh EH8 9XD, UK.

[*] Correspondence: pekka.postila@utu.fi

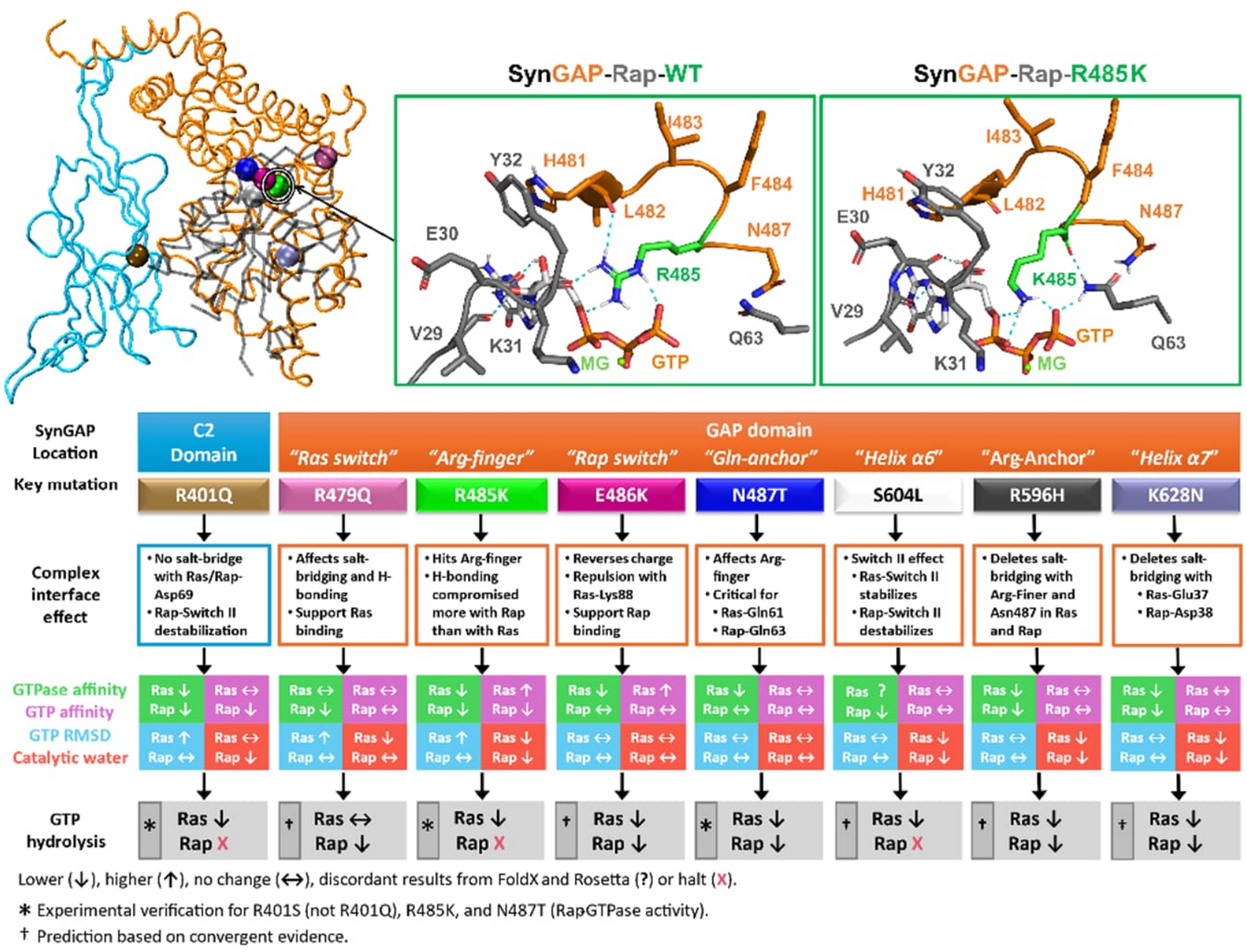

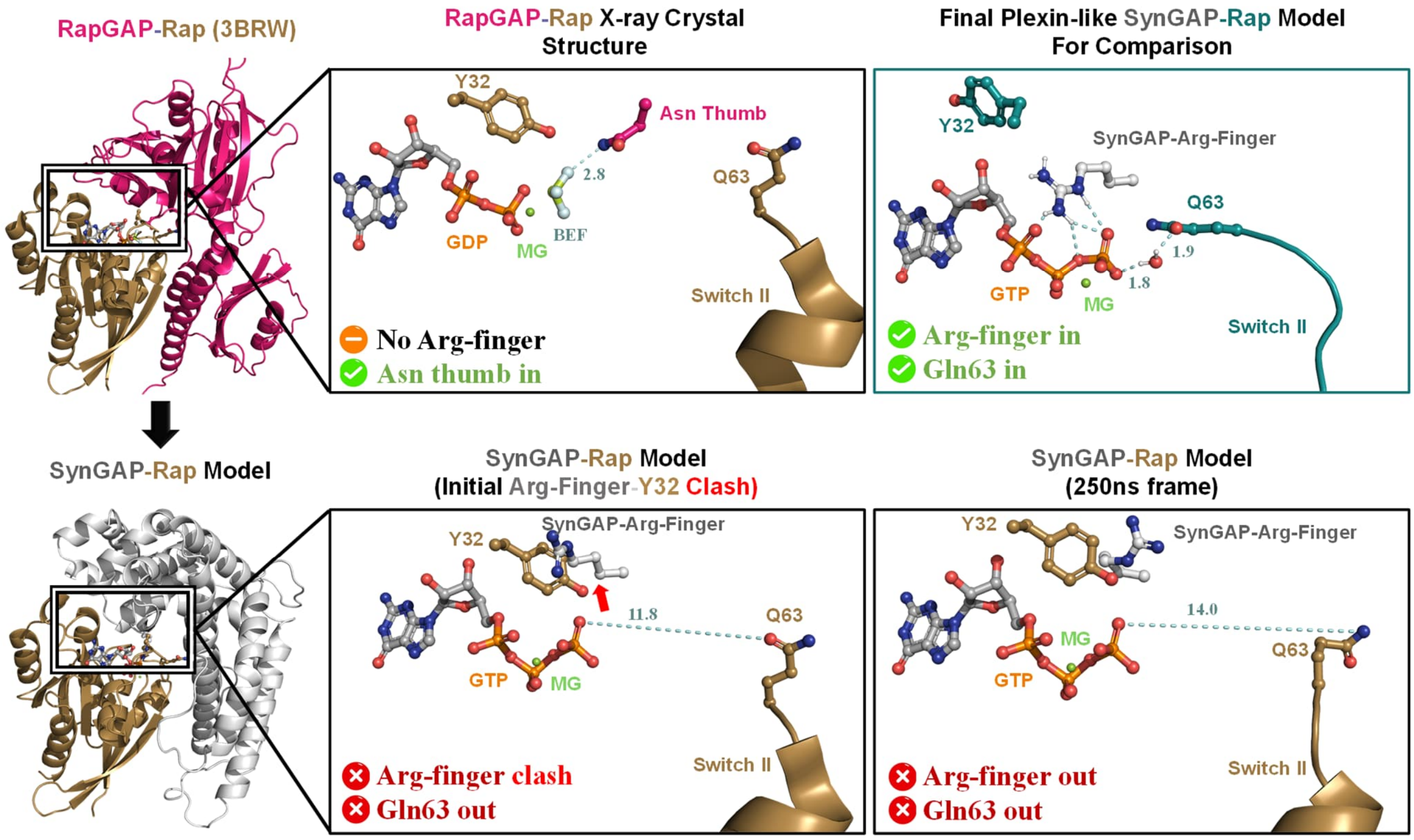

Figure S1. Structural incompatibility of SynGAP with the canonical RapGAP–Rap architecture. (Top left and middle) The canonical RapGAP–Rap X-ray crystal structure (PDB: 3BRW[1]) utilizes an Asn-thumb (pink) for catalysis rather than an Arg-finger. In this conformation, Rap-Tyr32 (tan) occupies the active site, and the Switch II Gln63 is oriented distally ("out") from the GDP/Mg^{2+}/BEF complex. (Bottom left and middle) A superimposition of SynGAP onto this canonical Rap architecture forces the SynGAP Arg-finger (white sticks) and Rap-Tyr32 into the same spatial coordinates, creating a severe steric clash (red arrow). (Bottom right) Following 250 ns of molecular dynamics simulation, this superimposition-based model fails to resolve into a catalytically competent state; the Arg-finger is away from the GTP ("out"), and Gln63 remains distal from the catalytic site. (Top right) For comparison, the final Plexin-like SynGAP–Rap model successfully resolves this incompatibility. Rap-Tyr32 (teal) uptakes an "open conformation", allowing the SynGAP Arg-finger to be inserted directly into the active site ("in") while switch II adopts to an open loop conformation bringing Gln63 "in" analogous to the "Asn thumb". For further details regarding the system build-up of the Plexin-based SynGAP–Rap complex, see Fig. 2.

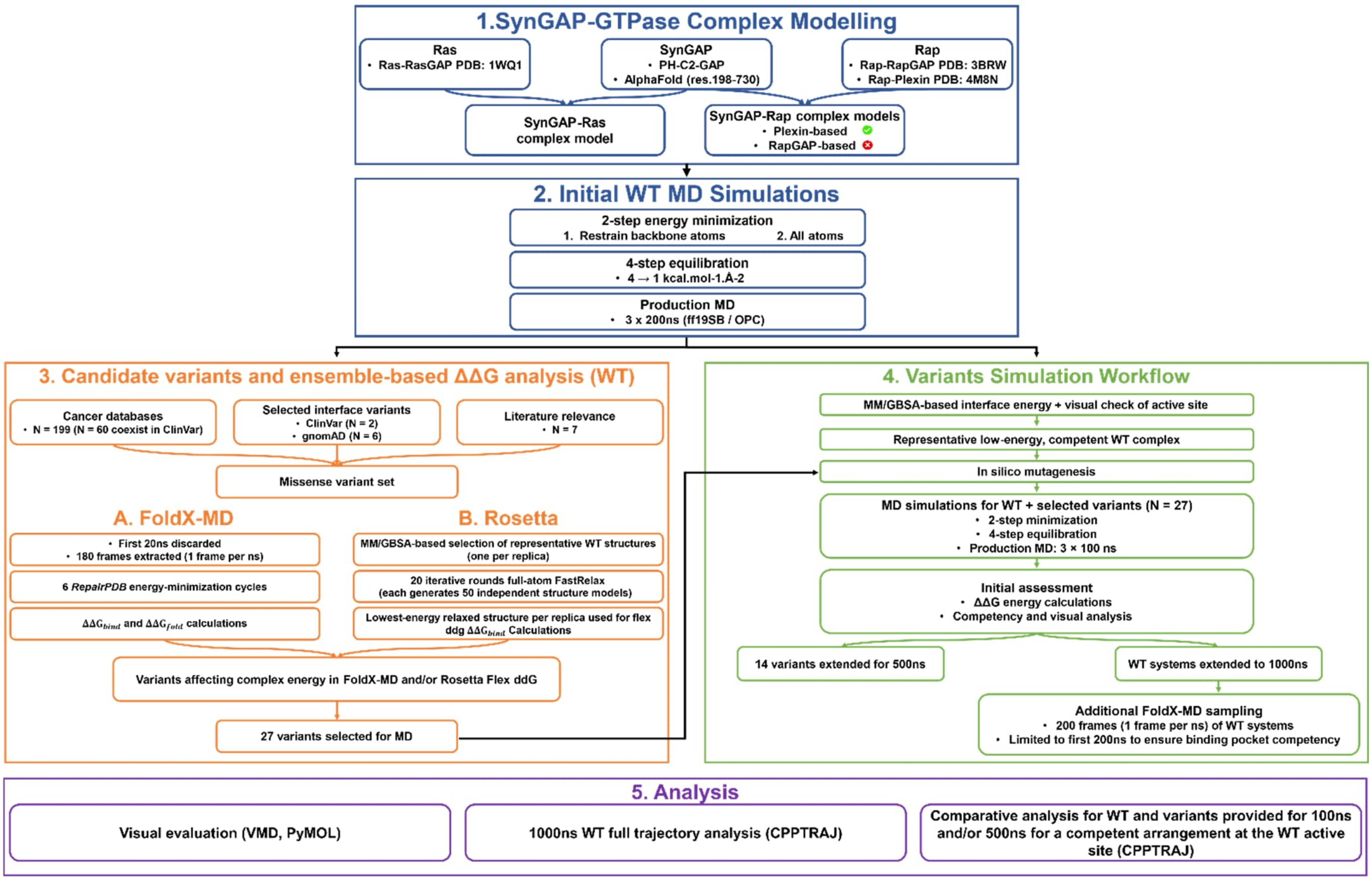

Figure S2. Computational workflow for SynGAP–GTPase complex modelling and variant analysis. The workflow summarizes the modelling, energetic screening, and molecular dynamics (MD) simulations of SynGAP–GTPase complexes. The WT-SynGAP-Ras/Rap complexes were initially modelled and simulated in three 200-ns replicas. The candidate missense variants were selected from cancer-associated datasets and supplemented with additional interface variants from ClinVar, gnomAD, and the literature. FoldX-MD and Rosetta Flex ddG were used to evaluate missense variant effects on complex stability, and variants predicted to affect complex energy (N = 27) were prioritized for production MD simulations (100 ns). The WT-SynGAP-Ras/Rap systems were extended to 1000 ns and a subset of variants (N = 14) were also extended (500 ns) to enable more detailed structural and energetic characterization.

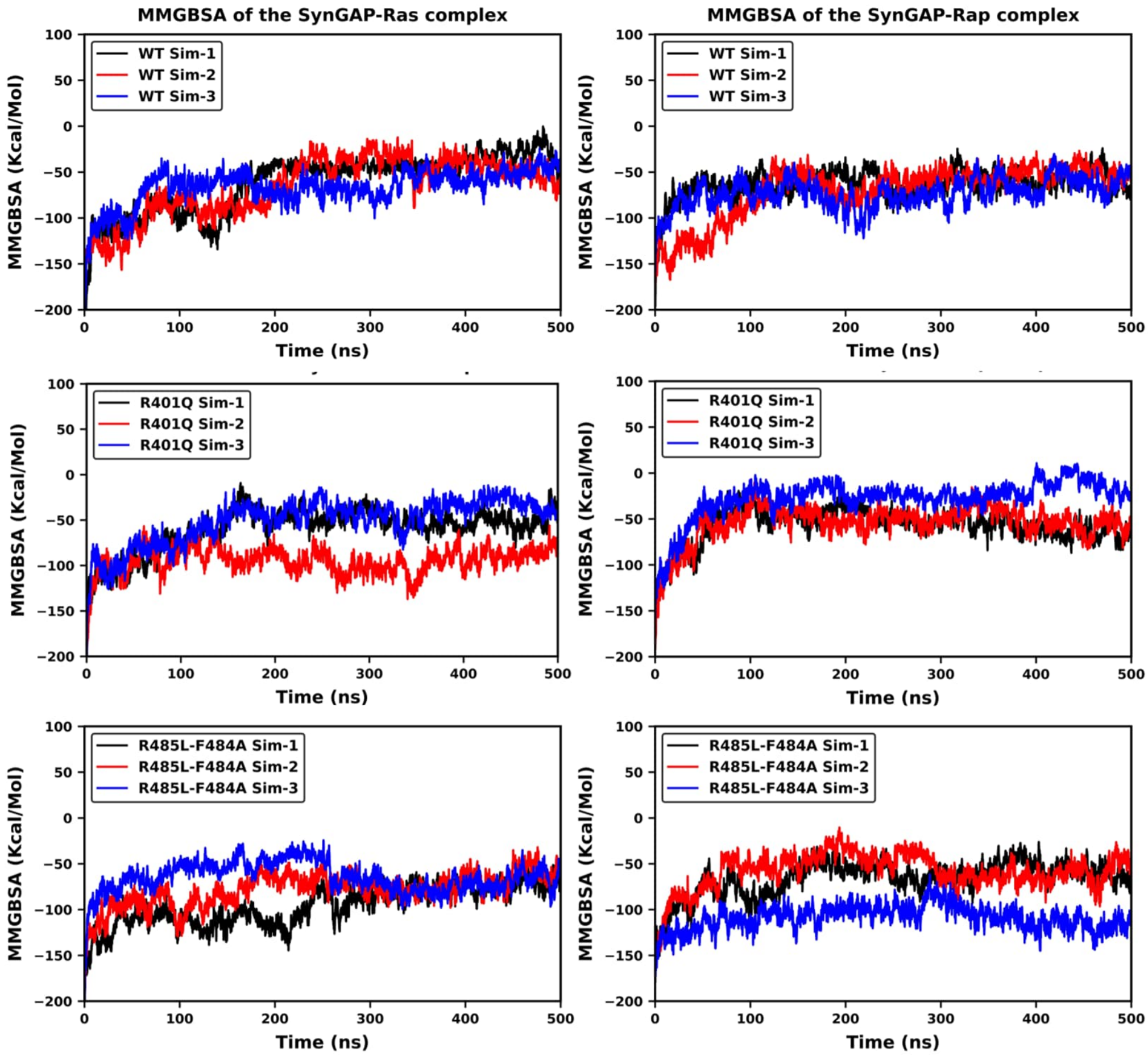

Figure S3. Binding free energy calculations show SynGAP–GTPase interface stability changes across SynGAP missense variants. The plots display representative binding free energies for missense variant trajectories on the protein–protein interface (SynGAP–GTPase) over 500 ns calculated using MM/GBSA (Molecular Mechanics with Generalized Born and Surface Area Solvation) methodology. These samples illustrate that while these mutations can impair catalytic activity, they do not cause a global dissociation of the complex within the simulated timeframe. In the SynGAP–Ras complex (on the left), there is a substantial overlap between the WT range and the profiles of the R401Q and R485L/F484A variants. These results suggest that the SynGAP–Ras interface remains "binding-competent" despite the mutations, indicating that the reduction in the GAP activity is likely driven by active-site misalignment rather than diminished protein–protein affinity. The SynGAP–Rap complex (on the right) similarly maintains a relatively stable interface across variants where the introduction of the R401Q and R485L/F484A mutations results in subtle energetic shifts. The SynGAP–GTPase complexes remain energetically stable throughout the 500 ns window. Each plot displays three independent 500 ns replicas (Black: Sim-1, Red: Sim-2, Blue: Sim-3) using a 20-frame moving average for visual clarity.

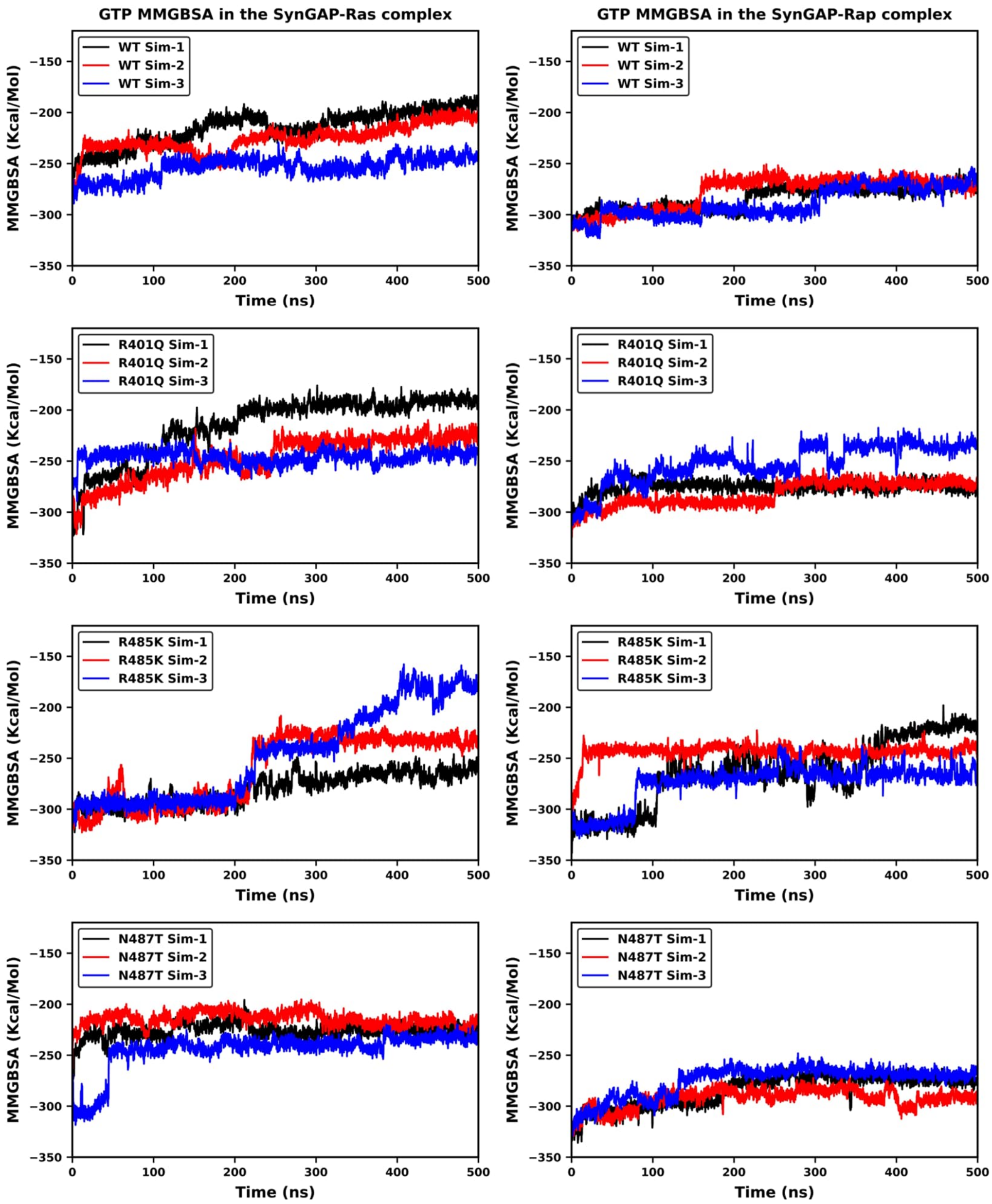

Figure S4. Binding free energy changes for GTP within Ras and Rap catalytic pockets. The plots display binding free energy trajectories (kcal/mol) for GTP over 500 ns calculated using MM/GBSA method. These data represent selected variants to illustrate typical energetic behaviour of the active-site across different variants. In the SynGAP–Ras complex (on the left), the WT binding energy is notably less stable than that of the Rap counterpart, with values typically ranging between -200 and -275 kcal/mol. The high inter-replica standard deviation (SD) observed in the WT trajectories reflects a dynamic and "loose" catalytic pocket. This inherent variability effectively masks the relative impact of the missense mutations with Ras, as their energetic fluctuations often overlap with the WT range. Conversely, the SynGAP–Rap complex (on the right) exhibits an overall more favourable energy profile. Although all WT replicas undergo a step-wise transition, shifting from an initial baseline of ~ -300 kcal/mol to a slightly less favourable, yet remarkably stable plateau near -280 kcal/mol. The markedly lower binding free energy in the Rap trajectories indicates a more rigid and tightly coordinated catalytic environment. Each plot displays three independent 500 ns replicas (Black: Sim-1, Red: Sim-2, Blue: Sim-3) using a 20-frame moving average for visual clarity.

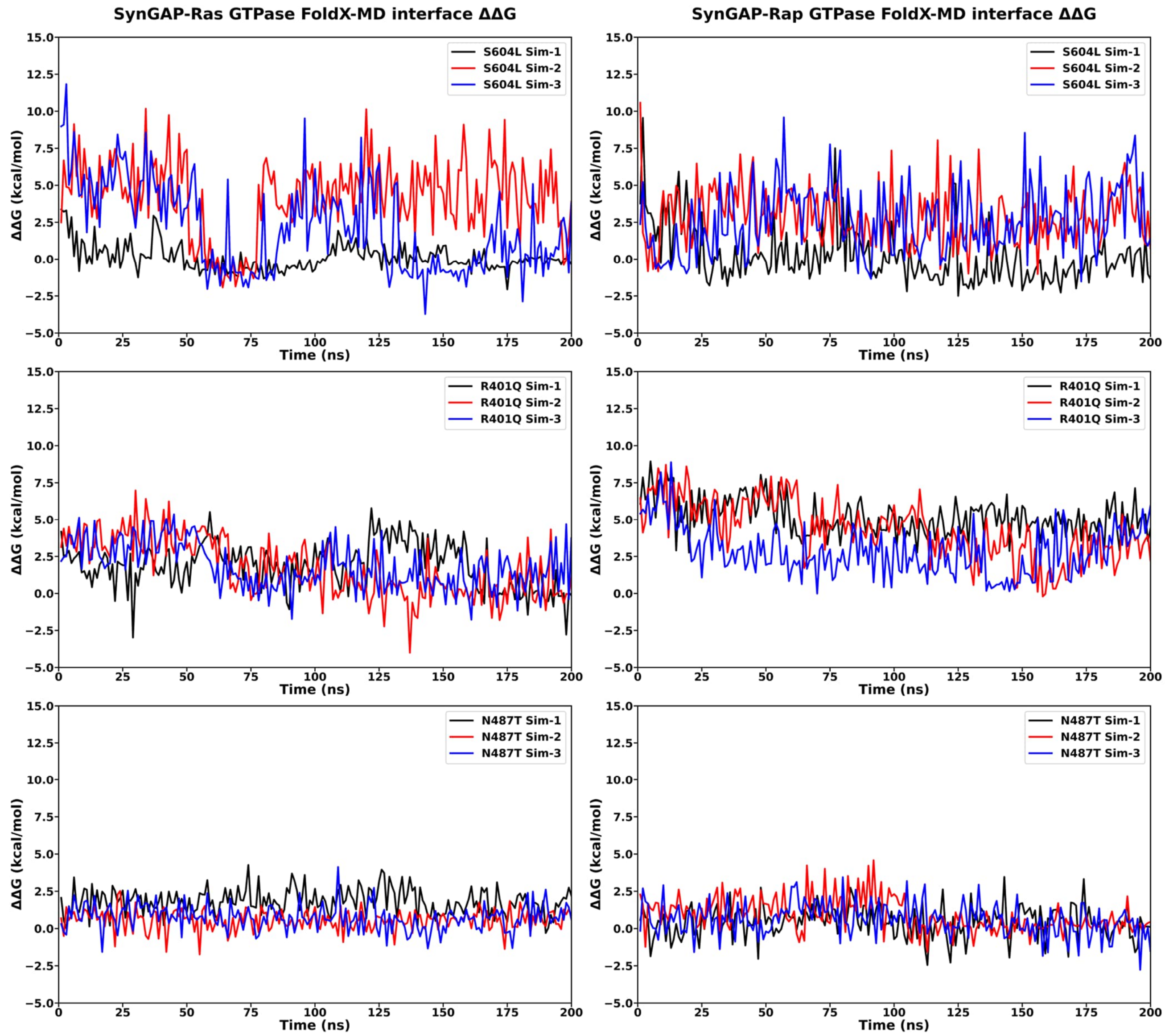

Figure S5. FoldX-MD analysis reveals variant-dependent changes in SynGAP–GTPase interface energetics. The plots show representative FoldX AnalyseComplex ΔΔG profiles for the S604L, R401Q, and N487T variants across the additional 200 ns of simulation sampling, using one frame per ns. The SynGAP–Ras complexes are shown on the left and the corresponding SynGAP–Rap complexes on the right. All three variants show positive ΔΔG fluctuations from lower-energy states, with differences in their magnitude and persistence across variants and replicas. These increases are not maintained throughout the trajectories, and energy fluctuates between higher and lower ΔΔG values, suggesting transient interface destabilization rather than sustained loss of binding or complete complex dissociation. Positive ΔΔG values indicate destabilization relative to WT, whereas negative values indicate stabilization. Each plot shows three independent replicas (black: Sim-1, red: Sim-2, blue: Sim-3).

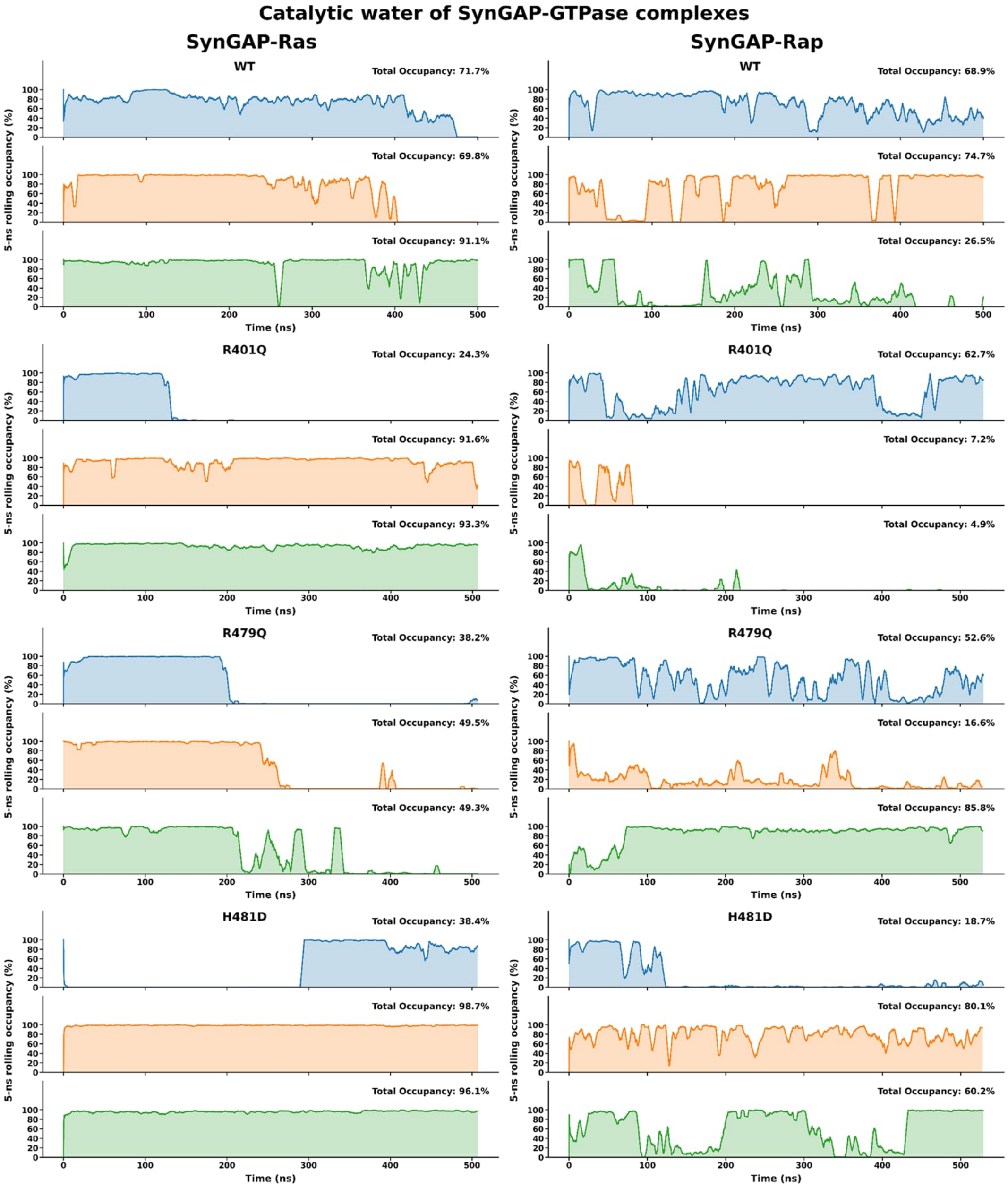

Figure S6. Catalytic water bridge occupancy profiles across SynGAP missense variants in SynGAP–Ras and SynGAP–Rap complexes. Time-series plots display the 5-ns rolling occupancy (%) of the catalytic water bridge over 500-ns molecular dynamics trajectories for wild-type (WT) SynGAP and 14 missense variants in complex with Ras (left panels) and Rap (right panels). Three independent replicas per system are shown (Blue: Sim-1, Orange: Sim-2, Green: Sim-3). H-bond distances and angles were computed using the *hbond bridgeout* in CPPTRAJ[2], considering the water molecule closest to the GTP γ-phosphate at each simulation frame. Water classified as coordinated only when simultaneously satisfying both the H-bond angle and distance requirements (≤3.6 Å heavy-atom distance and ≥135° Acceptor–H–Donor angle) between the catalytic Gln and GTP. In WT-Ras, the water bridge exhibits high initial stability but eventually breaks down and decreases over time. In contrast, WT-Rap displays a highly dynamic, fluctuating water network throughout the simulation. While many mutations (e.g., K628N, S604L) severely disrupt coordination entirely, several variants (e.g., H481D) maintained or exceeded WT retention levels in some replicas. This hyper-stability likely stems from localized pocket rigidification or compensatory interactions that unnaturally trap the water molecule.

Figure S6. Continues

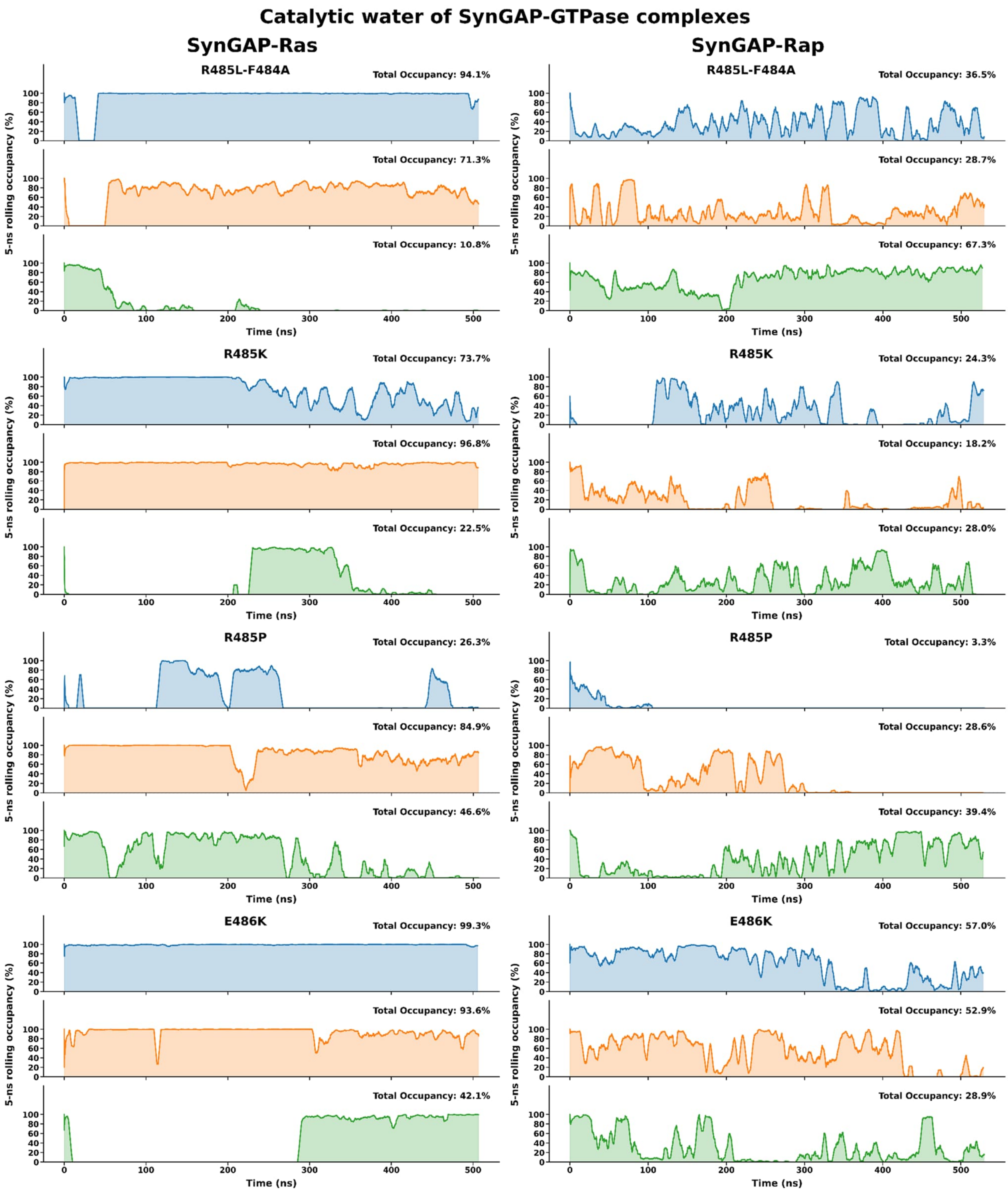

Figure S6. Continues

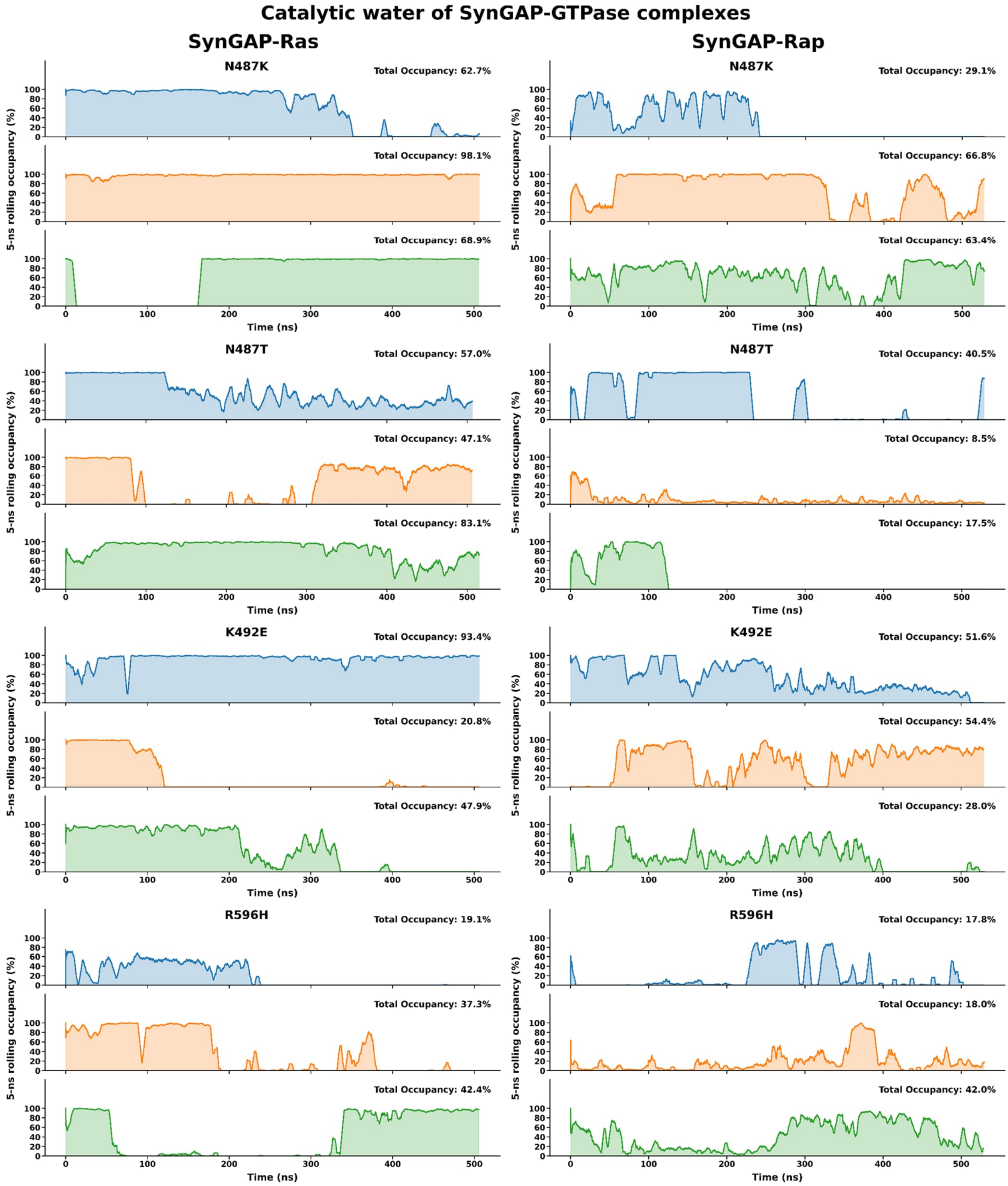

Figure S6. Continues

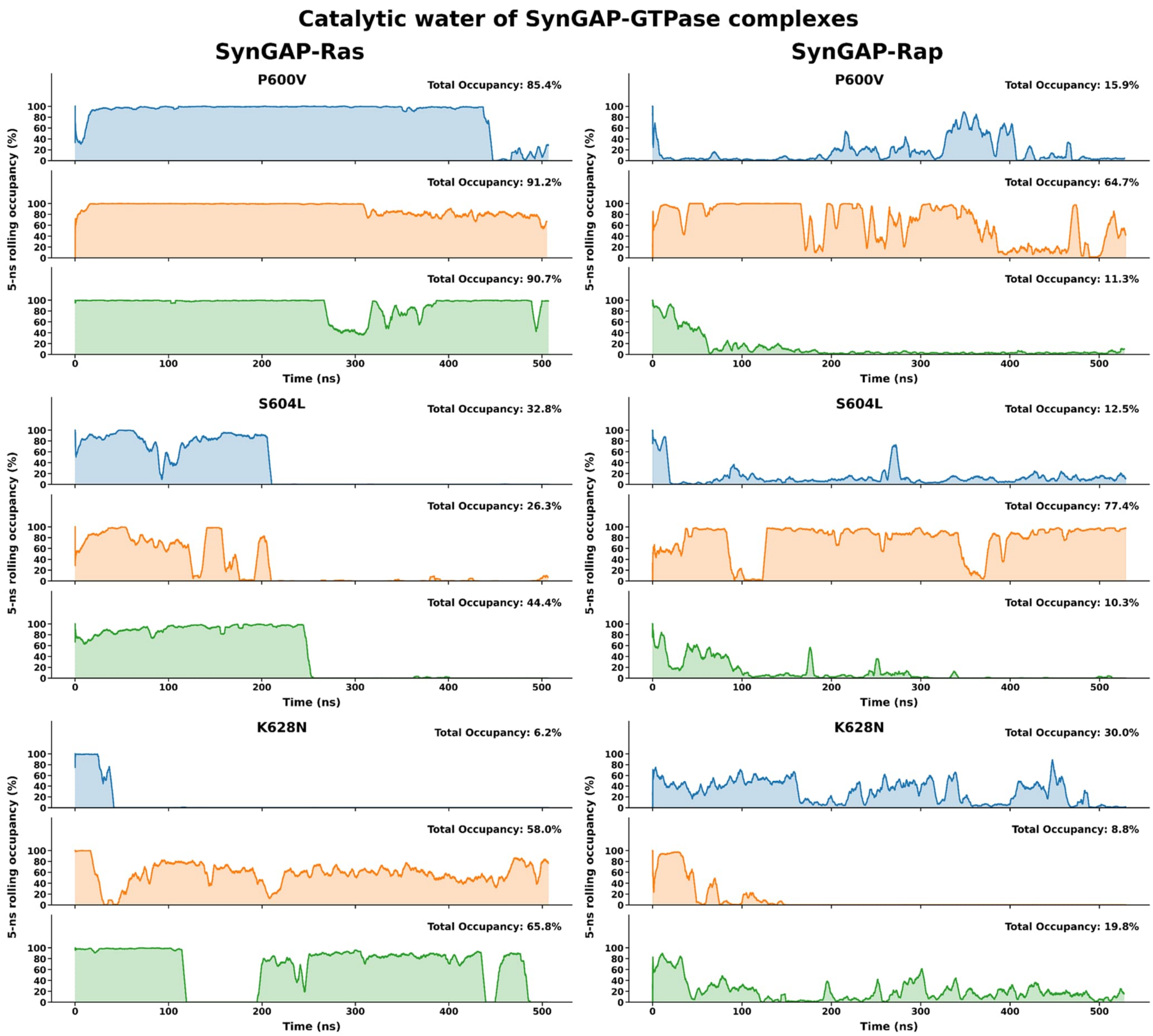

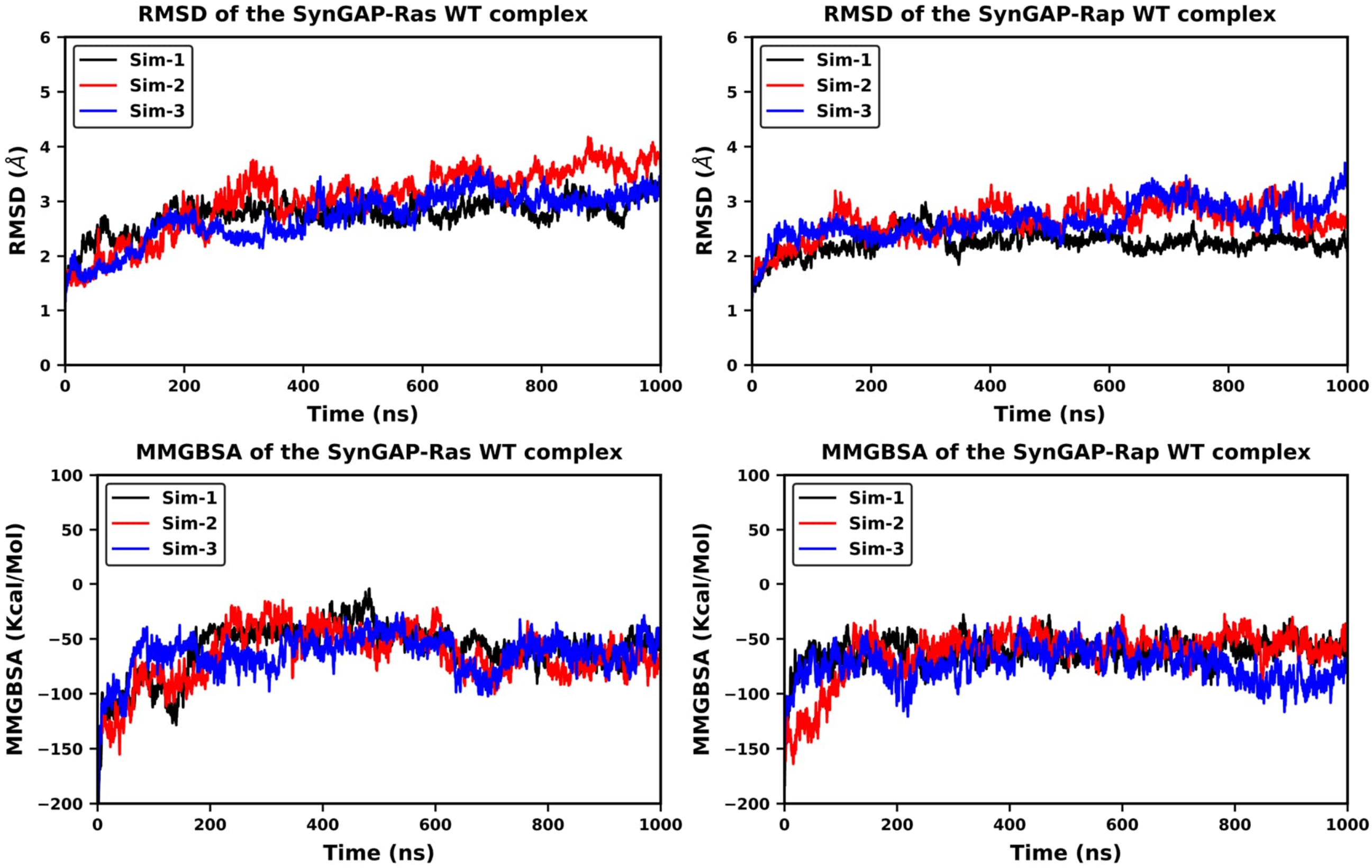

Figure S7. Global structural stability and protein–protein interface binding free energies of wild-type SynGAP–Ras and SynGAP–Rap complexes over 1-µs trajectories. (A) Root-mean-square deviation (RMSD) trajectories track the overall backbone stability of the WT SynGAP–Ras (top left) and SynGAP–Rap (top right) complexes over 1 µs across three independent replicas (Black: Sim-1, Red: Sim-2, Blue: Sim-3). Both complexes display an initial equilibration phase during the first 200 ns before stabilizing**. (B)** Time-series MM/GBSA profiles show the binding free energy across the protein–protein interface for SynGAP–Ras WT (bottom left) and SynGAP–Rap WT (bottom right) over 1 µs. Following an initial surge, both complexes converge onto stable, favourable binding energy plateaus (~ -50 to -75 kcal/mol), demonstrating that the wild-type interfaces remain structurally intact and binding-competent throughout the extended 1-µs timeframe. All curves are displayed using a 50-frame moving average for visual clarity.

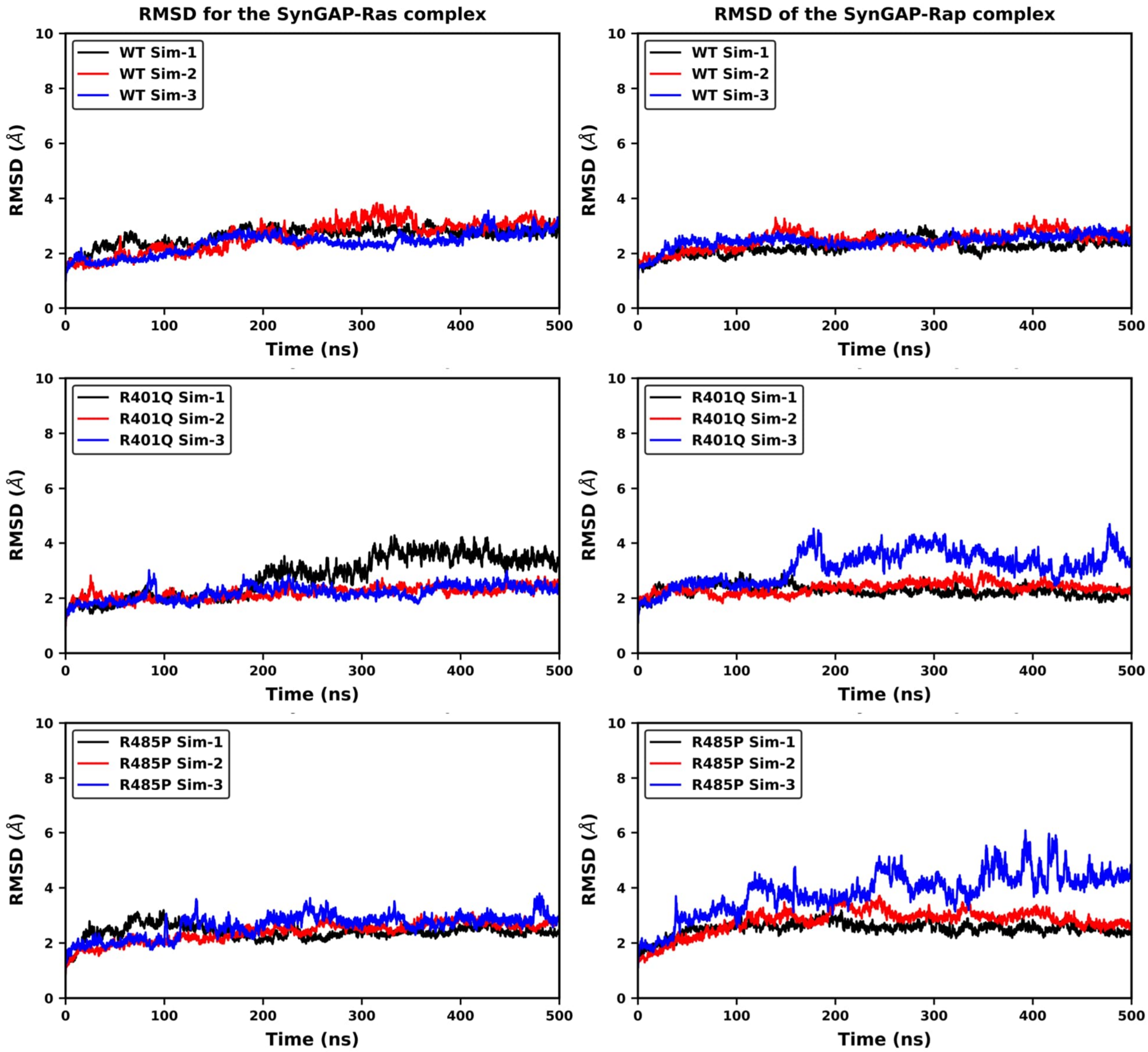

Figure S8. Protein backbone fluctuations demonstrate global structural stability of SynGAP–GTPase complexes. The plots show the RMSD (Root-Mean-Square-Deviation) of the SynGAP protein backbone atoms (Å) over 100 ns trajectories. These representative missense variants illustrate that the global fold of both SynGAP remains stable throughout the MD simulations, with no large-scale structural unfolding or shift across the different variants. In the SynGAP–Ras complex (on the left), the WT and variant trajectories (R401Q and R485P) maintain a consistent RMSD typically between 1.5 and 4.0 Å. The SynGAP–Rap complex (on the right) exhibits similar backbone stability, with trajectories equilibrating between 2.0 and 4.0 Å. The plot profiles suggest that the mutations primarily disrupt the catalytic core and/or complex affinity without compromising the overall structural integrity of the complex. The flexible Ω Gly-rich loop in the C2 domain was excluded from the calculations. Each plot displays three independent 500 ns replicas (Black: Sim-1, Red: Sim-2, Blue: Sim-3) using a 20-frame moving average for visual clarity.

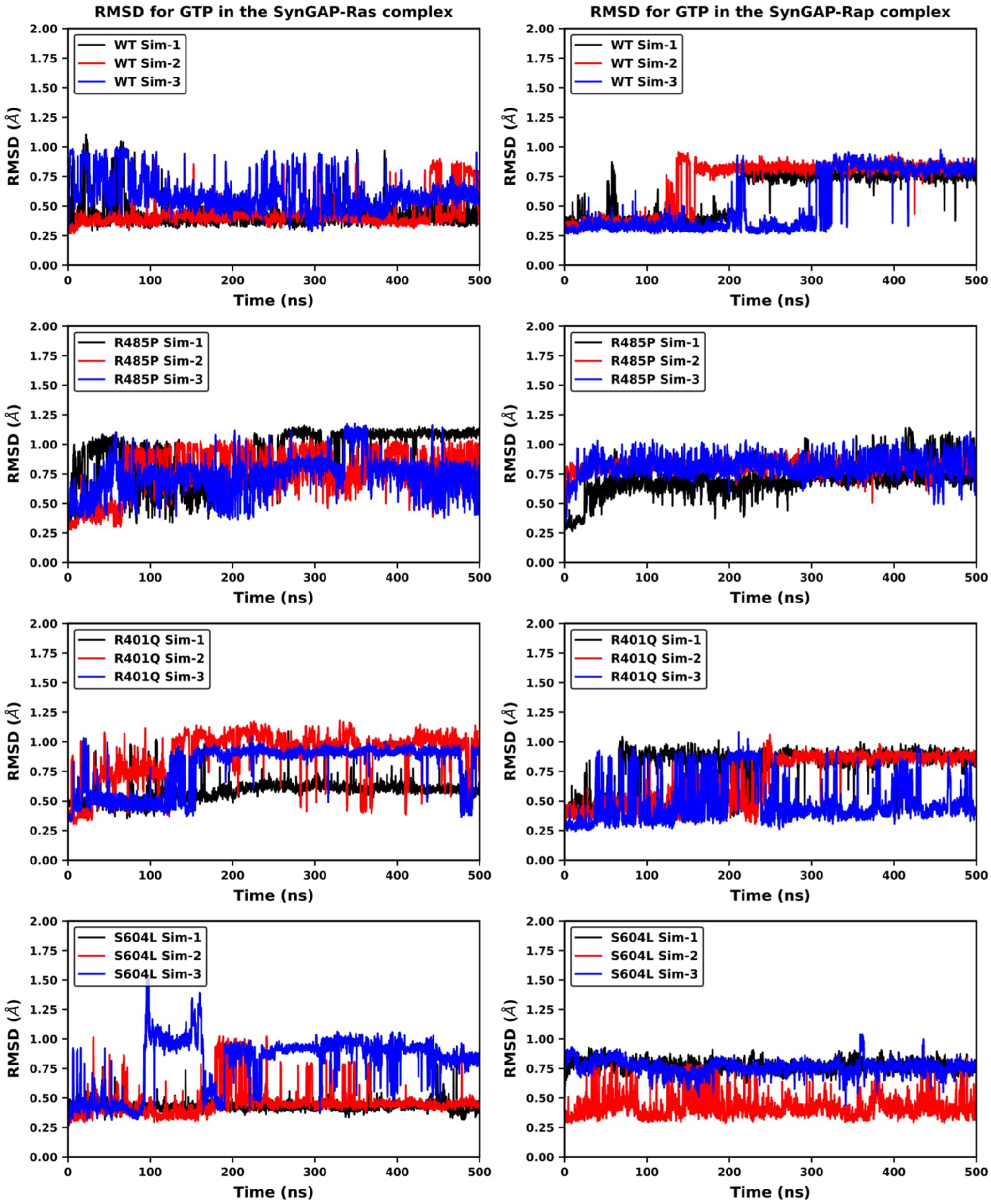

Figure S9. The GTP fluctuations highlight the differential positional stability of SynGAP missense variants. The plots show the root-mean-square deviation (RMSD) of GTP heavy atoms (Å) over 500-ns trajectories across three independent replicas (Black: Sim-1, Red: Sim-2, Blue: Sim-3). In the SynGAP–Ras complex (left), the WT trajectories generally maintain a stable coordination environment (~0.4–0.5Å) with notable fluctuations (0.3–1.0Å) primarily in Sim-3 replica. Similarly, GTP in the SynGAP–Rap WT complex (right) maintained low-frequency RMSD fluctuations; although all three WT replicas underwent a structural transition that elevated the ligand RMSD to ~0.8–0.9Å, GTP subsequently converged onto a stable plateau at this value. In contrast, variants, most notably R485P in both complexes, exhibited continuous RMSD oscillations (0.4–1.1Å) throughout the 500-ns trajectories, reflecting persistent dynamic instability.

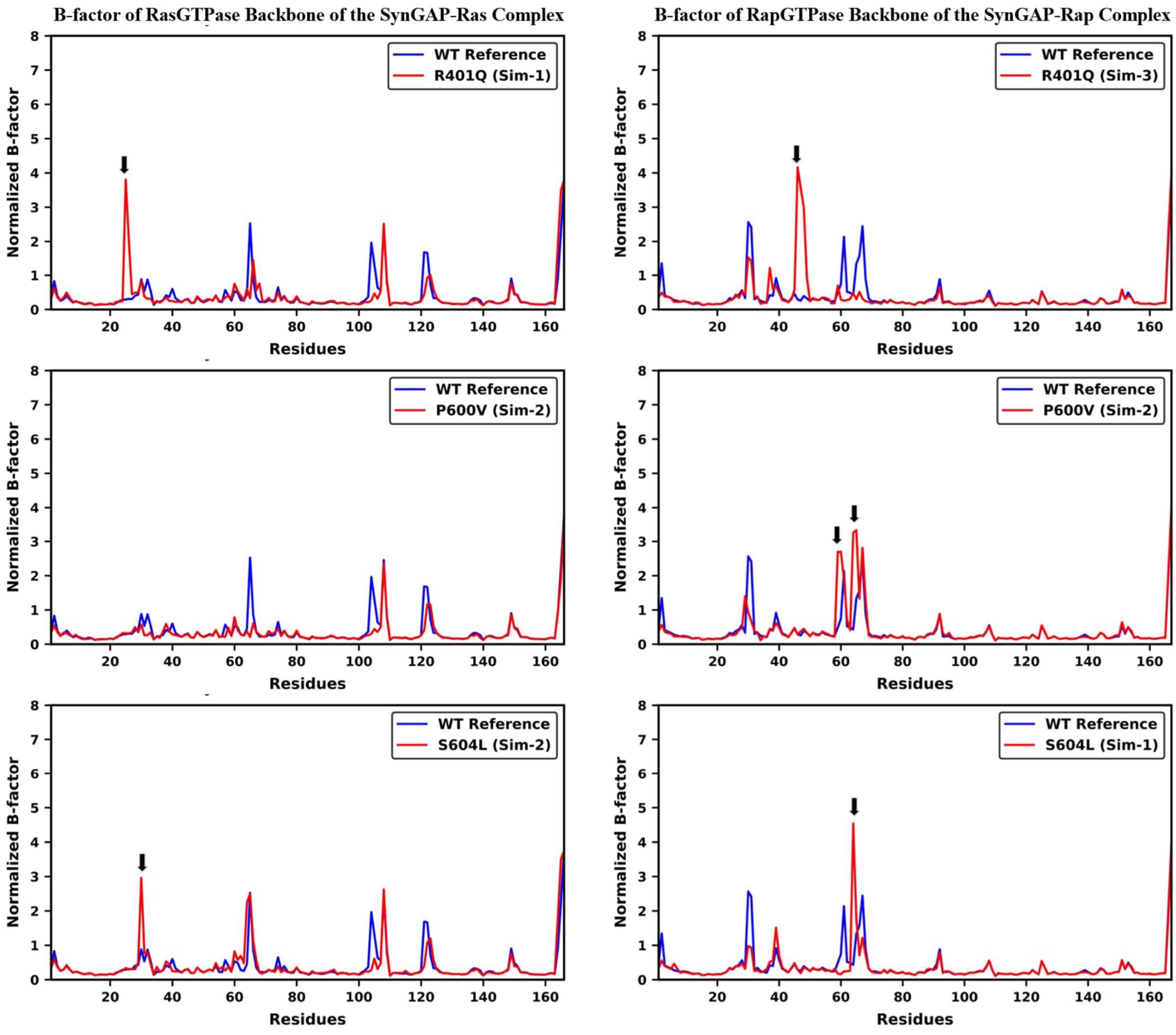

Figure S10. SynGAP missense mutations increase backbone atomic fluctuations in the GTPase-Switches. The plots compare the normalized backbone B-factors of the GTPase in selected variant replicas (red) against a WT baseline (blue). Black arrows highlight specific peaks in the Switch I and Switch II region (residues 30–37 and 60–76). While these increased fluctuations are observed in specific variants such as R401Q, P600V, and S604L, this effect is not uniform across all simulation replicas. In the SynGAP–Ras complex (on the left), arrows indicate localized spikes in Switch I B-factors, most notably in the R401Q and S604L variants. In the SynGAP–Rap complex (on the right), the variants exhibit increases in fluctuations that extend into the Switch II region. These localized events, captured in selected trajectories, correlate with the loss of the specific structural anchors and coordinating hydrogen bonds (H-bonds) required to maintain a catalytically competent active-site orientation. The WT profile represents the per-residue average of three 500 ns replicas representing the upper limit of native structural fluctuation. X-axis residue numbering corresponds to the GTPase (Ras or Rap) sequence.

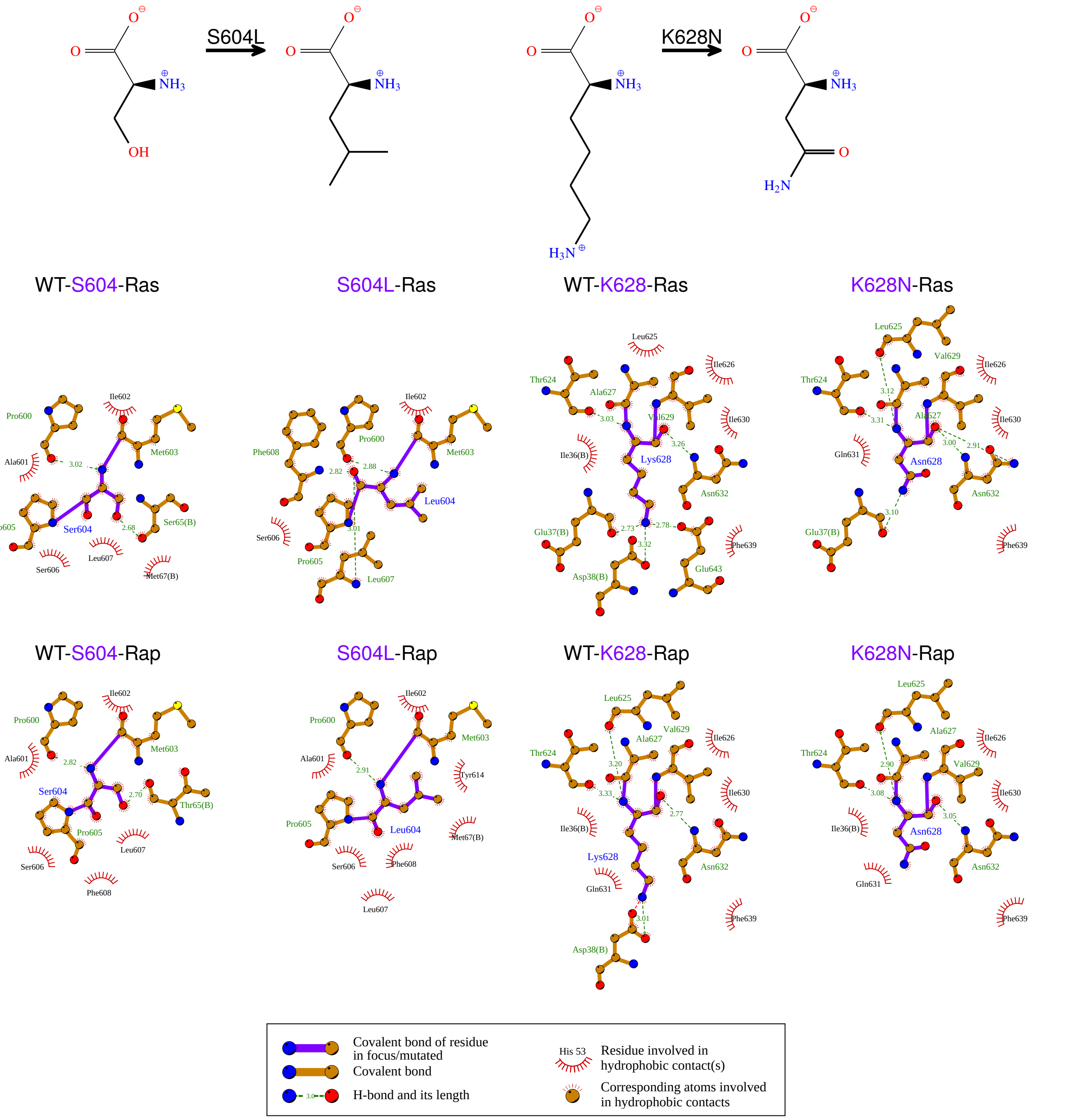

Figure S11. Interaction diagrams for the SynGAP missense mutations S604L and K628N with the Ras/Rap-GTPases. The WT-Ser604 and the mutated Leu604 for the missense mutation S604L and the WT-Lys628 and the mutated Asn628 for the missense mutation K628N are shown as 2D plots (bonds/carbons = black lines). The residues are highlighted (magenta bonds) in the WT and mutated systems as 2D interaction diagrams in complex with Ras-GTPase. Likewise, the corresponding WT and mutated residue are highlighted as interaction diagrams in complex with Rap GTPase. In all diagrams, amino acids are shown as ball-and-stick representations (bonds/carbons = orange), however, GTPase residues are assigned chain identifier B. Coordinates for both the WT and missense variant structures are the same 100-ns MD snapshots as seen in Fig. 5. Interaction diagrams were generated using LigPlot+ v.2.3.1[3]. For further interpretation see Fig. 5.

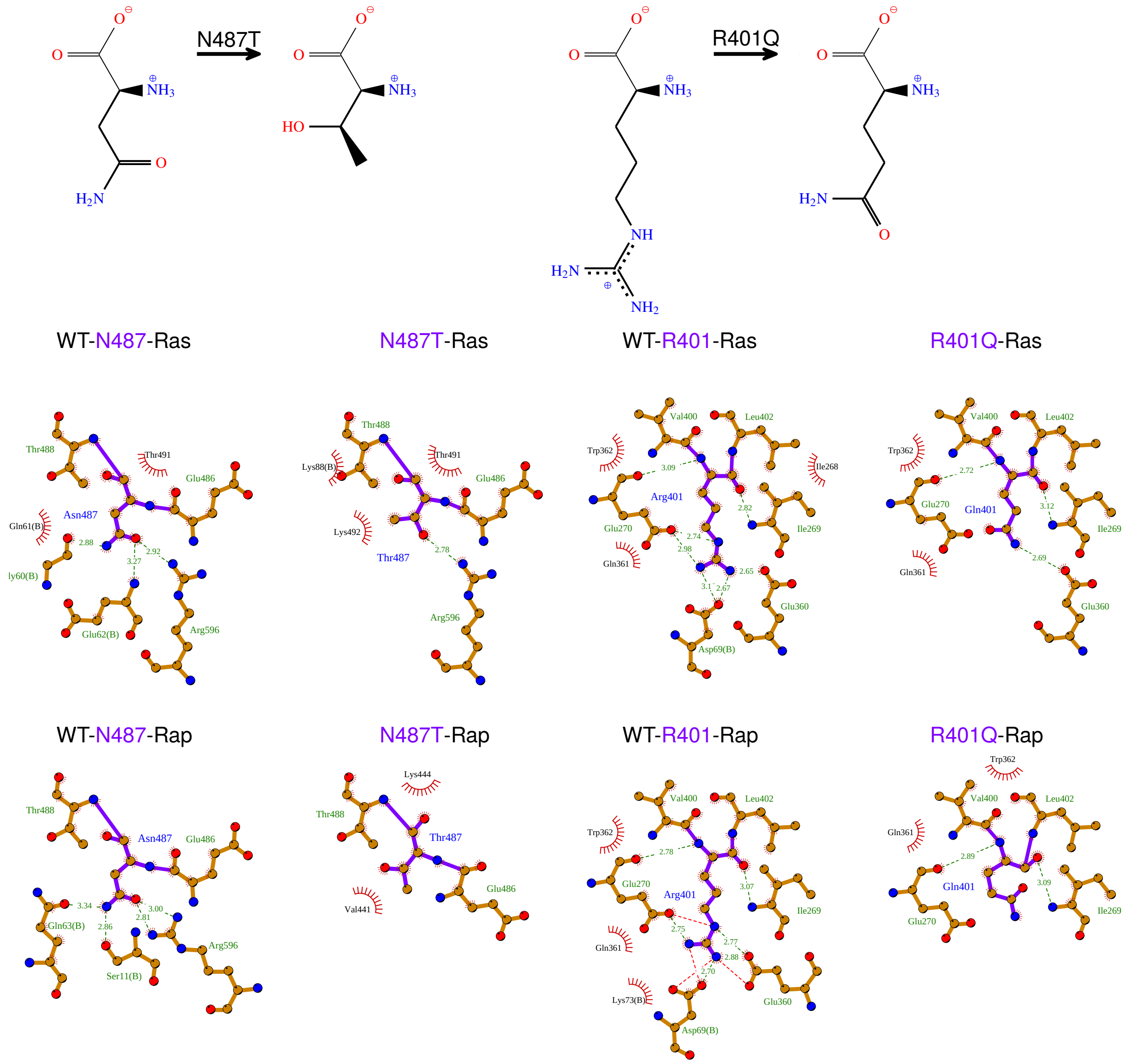

Figure S12. Interaction diagrams for the missense mutations N487T and R401Q with the Ras/Rap-GTPases. The WT-Asn487, the mutated Thr487 for the missense mutation N487T, the WT-Arg401 and the mutated Gln401 for the missense mutation R401Q are shown as 2D plots (bonds/carbons = black lines). The residue is highlighted (magenta bonds) in the WT and mutated systems as 2D interaction diagrams in complex with Ras-GTPase. Likewise, the corresponding WT and mutated residue are highlighted as interaction diagrams in complex with Rap GTPase. Coordinates for both the WT and missense variant structures are the same 100-ns MD snapshots as seen in Fig. 6. Interaction diagrams were generated using LigPlot+v.2.3.1[3]. For further interpretation see Figs. S11 and 6.

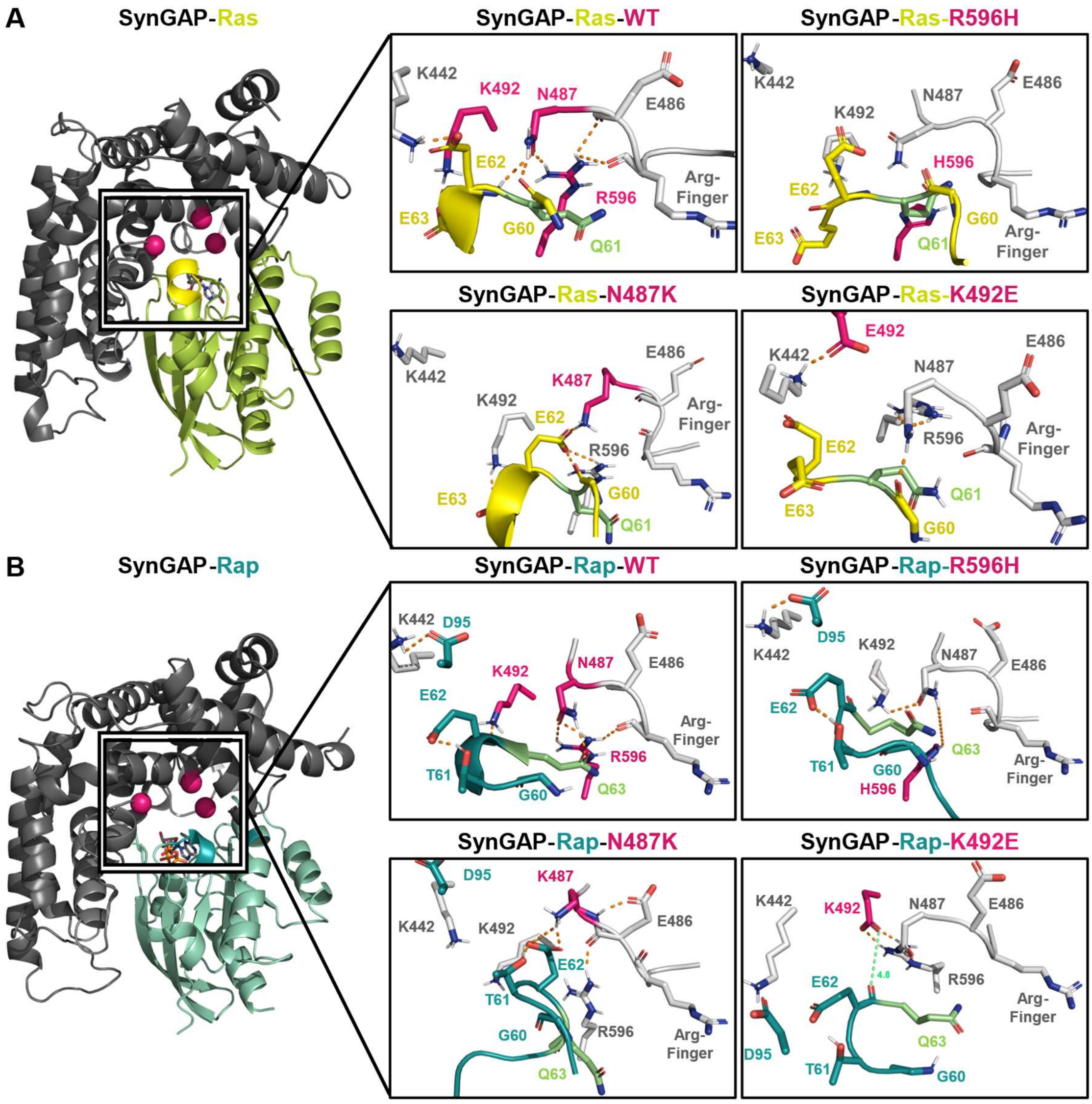

Figure S13. The Asn487-Lys492-Arg596 network stabilizes the catalytic core in the SynGAP–Ras/Rap complexes. (A) The overview (on the left) shows the location of the residues Arg596, Asn487, and Lys492 (magenta spheres) at the catalytic SynGAP–GTPase interface. In the WT-SynGAP–Ras interface, a comprehensive coordination network maintains the orientation of the Arg-finger and the catalytic Gln61 (sticks with green carbons). These stabilizing interactions are severely perturbed by the following two variants: 1) In the R596H variant, the substitution to histidine disrupts the central coordination of the catalytic loop. 2) The N487K mutation introduces a bulky lysine side-chain that sterically hinders the native interface. In the K492E variant, a charge-reversal mutation eliminates the salt-bridge with Ras-Glu62, perturbing the catalytic segment. **(B)** The overview (on the left) indicates the corresponding residues in the SynGAP–Rap complex. Similar to the SynGAP–Ras system, the WT-SynGAP–Rap interface relies on the precise positioning of the Arg-finger and Gln63 (green sticks) via the Asn487, Lys492 and Arg596 network. The variants cause a breakdown in the local H-bonding network, destabilizing the rigid environment necessary for efficient GTP hydrolysis. All panels show SynGAP in grey and Ras/Rap in yellow/teal, respectively. H-bonds are indicated by orange dashed lines and interatomic distances by green dashed lines. Key residues are shown as sticks, and, in the overview panels, GTP is visible at the active-site. See Fig. S14 for 2D interaction diagrams.

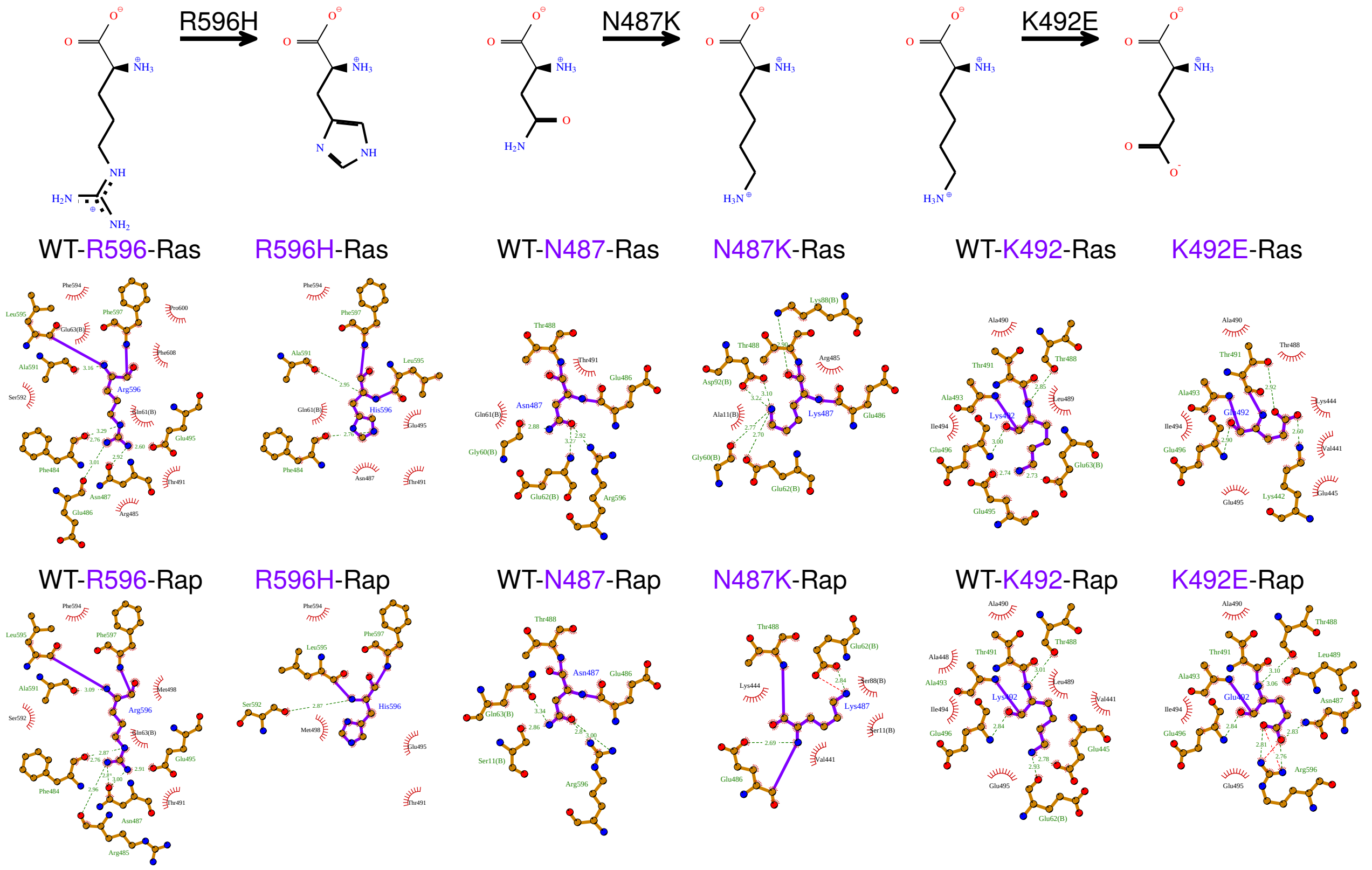

Figure S14. Interaction diagrams for the SynGAP missense variants R596H, N487K, and K492E with the Ras/Rap-GTPases. The WT-Arg401/Asn487/Lys495 and the mutated His596/Lys487/Glu492 for the missense mutations R596H, N487K, and K492E are shown as 2D plots (bonds/carbons = black lines). The residues are highlighted (magenta bonds) in the WT and mutated systems as 2D interaction diagrams in complex with Ras-GTPase. Likewise, the corresponding WT and mutated residues are highlighted as interaction diagrams in complex with Rap GTPase. Coordinates for both the WT and missense variant structures are the same 100-ns MD snapshots as seen in Fig. S13. For further interpretation see Figs. S11 and S13.

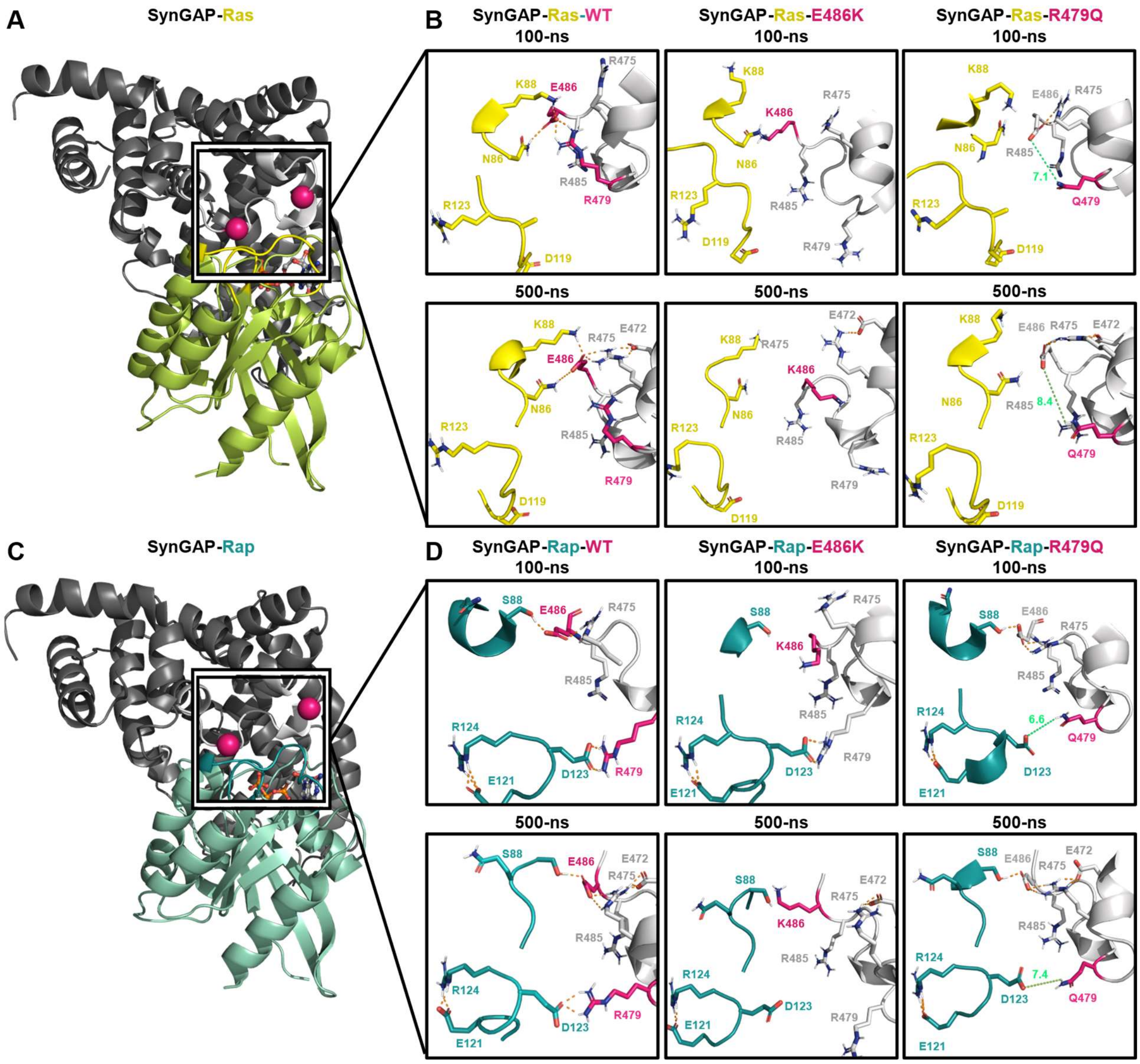

Figure S15. Distinct stabilization of SynGAP–Ras and SynGAP–Rap interfaces by the Arg479–Glu486 interaction network. **(A)** The overview of the SynGAP–Ras complex indicating the locations of Arg479 and Glu486 (magenta spheres). **(B)** In the WT-SynGAP–Ras interface at 100 ns, Glu486 (sticks with magenta carbons) forms a H-bond network with Ras residues Lys88 and Asn86, while maintaining an intramolecular salt-bridge with Arg479. By 500 ns, the WT structure starts to relax and the internal Glu486–Arg479 salt bridge breaks, yet the interface remains relatively intact. The E486K charge-reversal mutation disrupts the essential coordination with Ras-Lys88 and SynGAP-Arg479 early on, and this distal interface remains severely destabilized continuously through 500 ns. While in the R479Q variant, the substitution leads to the sustained loss of this internal salt-bridge. **(C)** The overview of the SynGAP–Rap complex indices the locations of Arg479 and Glu486 (magenta spheres). **(D)** In the WT-SynGAP–Rap interface, Arg479 (sticks with magenta carbons) anchors the complex by forming a salt-bridge with Rap-Asp123, while Glu486 interacts with Rap-Ser88 and SynGAP-Arg475 and these interactions remain remarkably stable through 500 ns. The E486K missense variant introduces a positive charge at the interface that repels SynGAP-Arg475. Unlike at 100 ns, this local coordination completely breaks by 500 ns. This indicates that while the destabilizing effect of the E486K mutation is severe and immediate in Ras, it eventually disrupts the Rap interface over longer simulation timescales. In the R479Q missense variant, the critical salt-bridge between residue 479 and Asp123 is broken, which disrupts the SynGAP–Rap complex stability. See Figs. S16 and S17 for 2D interaction diagrams.

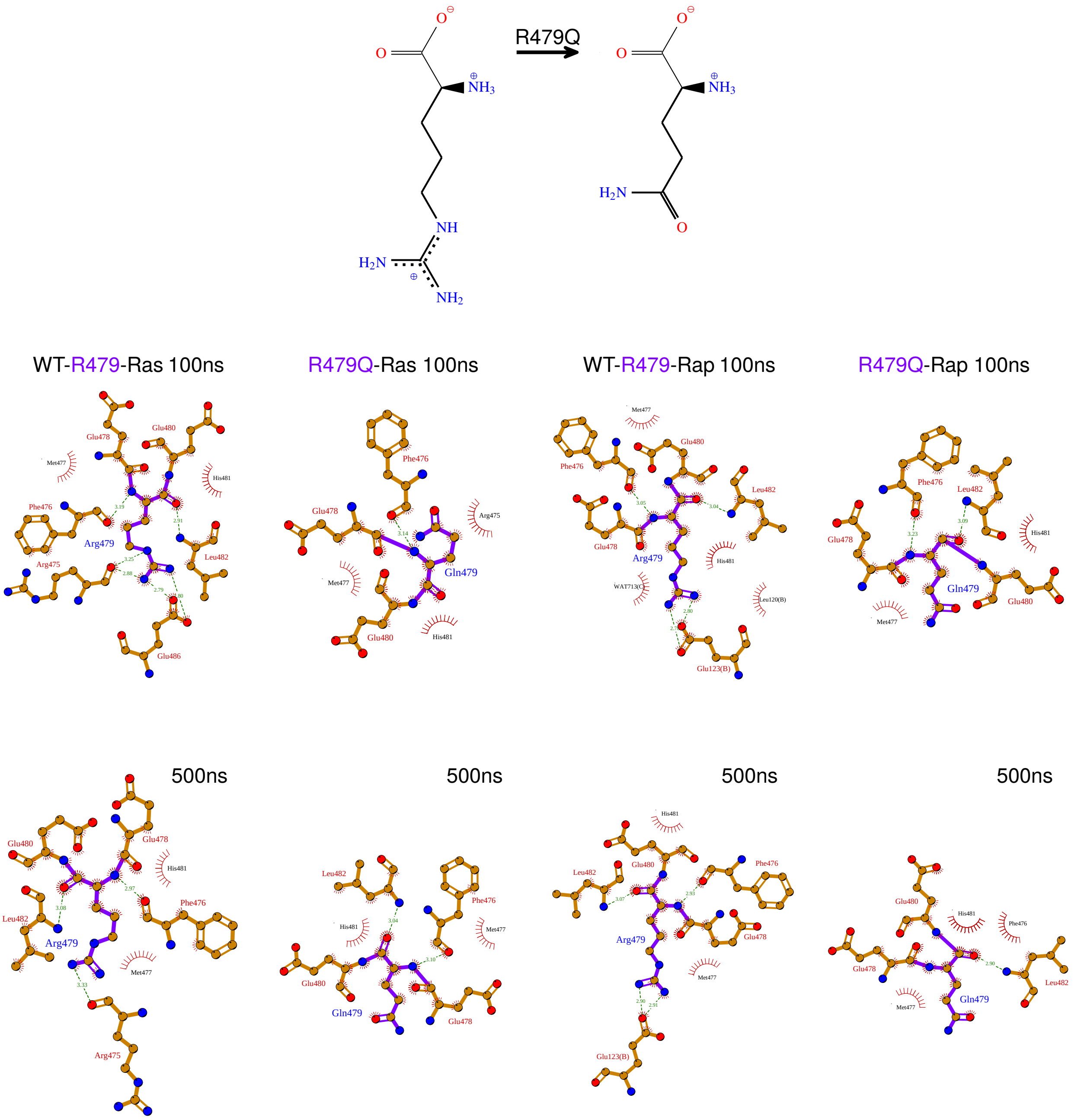

Figure S16. Interaction diagrams for the missense mutation R479Q with the Ras/Rap-GTPases. The WT-Arg479 and the mutated Gln479 for the missense mutation R479Q are shown as 2D plots (bonds/carbons = black lines). The residues are highlighted (magenta bonds) in the WT and mutated system as 2D interaction diagrams in complex with Ras-GTPase.) Likewise, the corresponding WT and mutated residue are highlighted as interaction diagrams in complex with Rap GTPase. Coordinates for both the WT and missense variant structure are the same 100-ns and 500-ns MD snapshots as seen in Fig. S15. For further interpretation see Figs. S11 and S15.

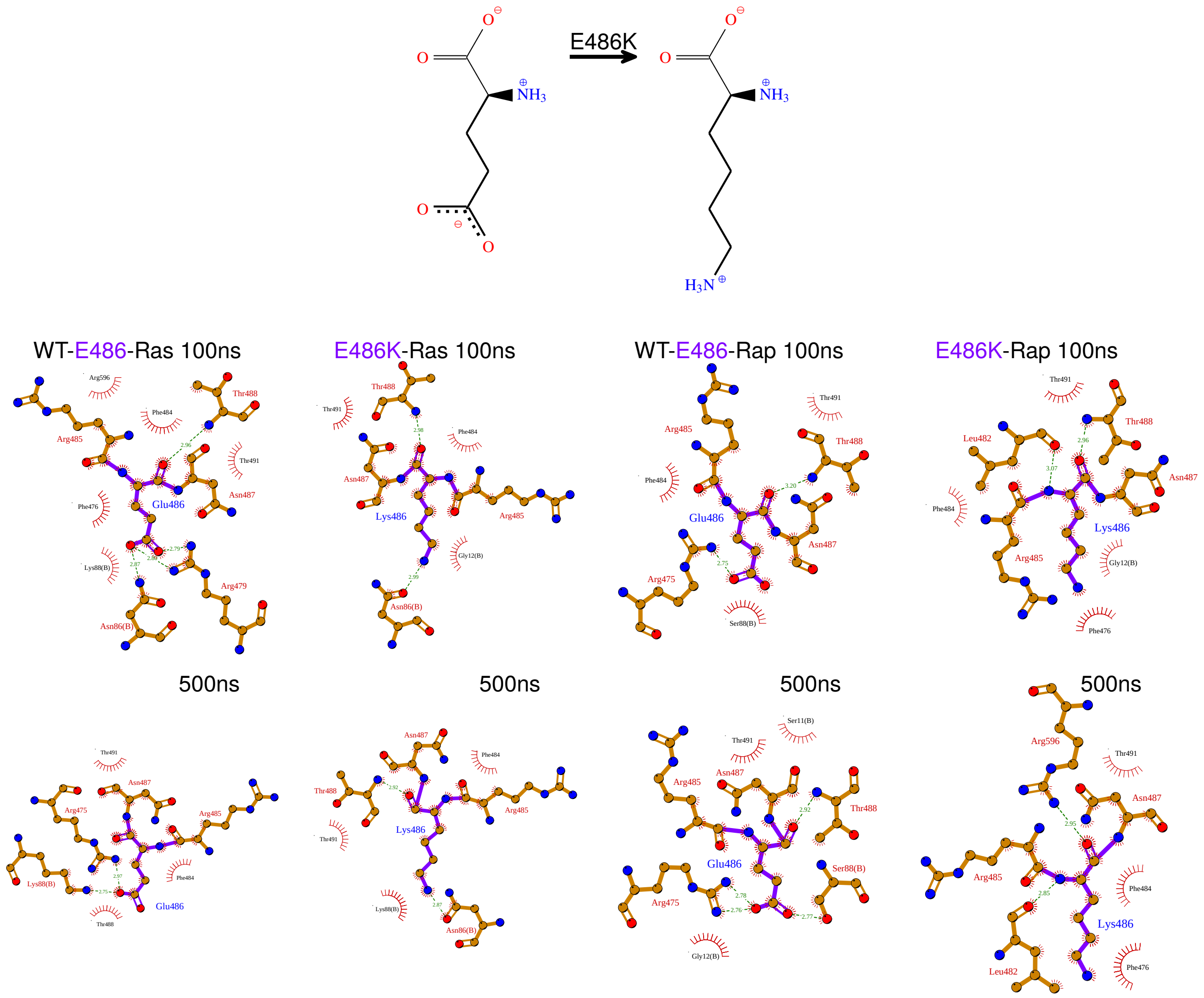

Figure S17. Interaction diagrams for the missense mutation E486K with the Ras/Rap-GTPases. The WT-Glu486 and the mutated Lys486 for the missense mutation E486K are shown as 2D plots (bonds/carbons = black lines). The residues are highlighted (magenta bonds) in the WT and mutated system as 2D interaction diagrams in complex with Ras-GTPase.) Likewise, the corresponding WT and mutated residue is highlighted as interaction diagrams in complex with Rap GTPase. Coordinates for both the WT and missense variant structures are the same 100-ns and 500-ns MD snapshots as seen in Fig. S15. For further interpretation see Figs. S11 and S15.

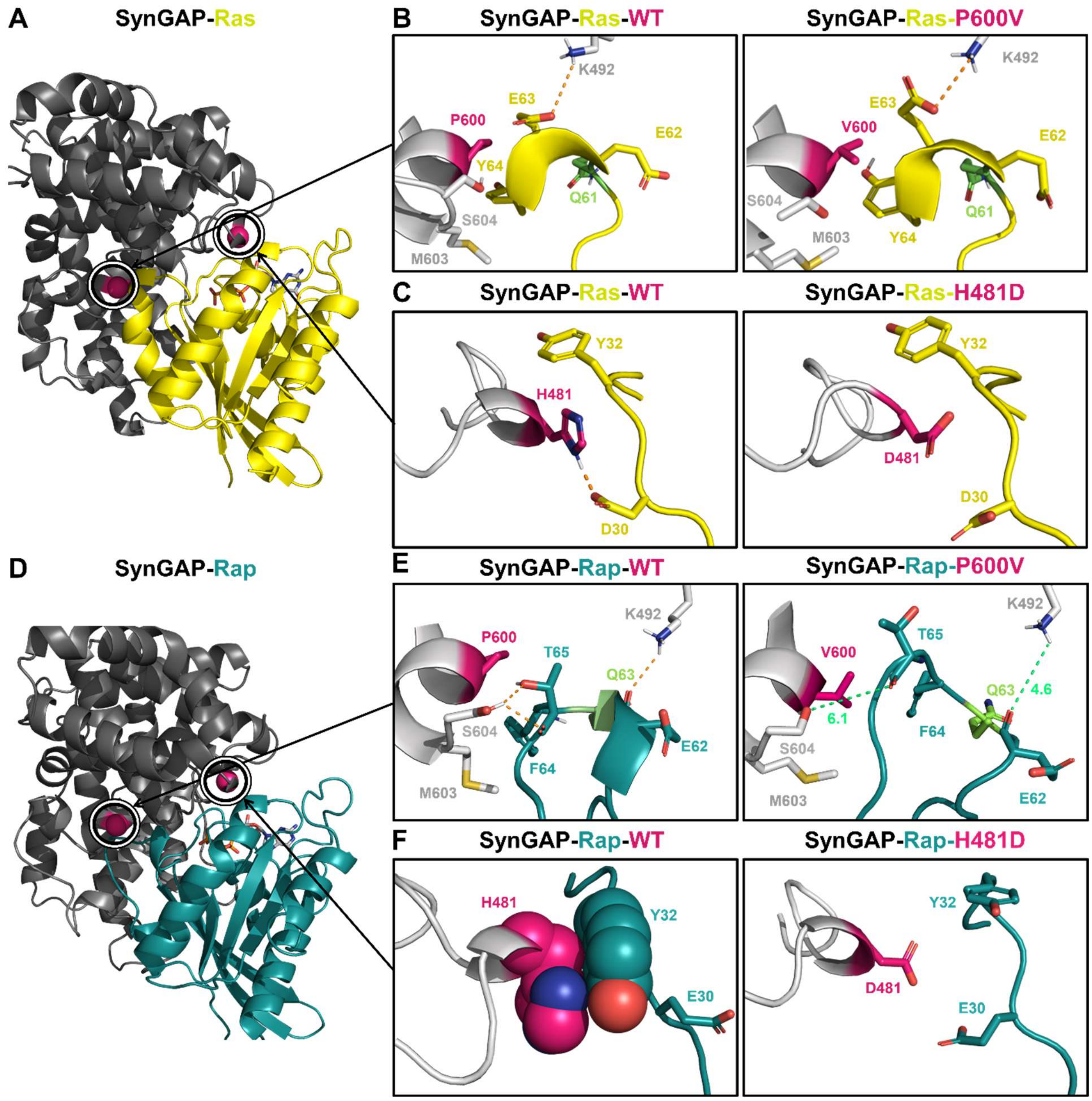

Figure S18. Impact of mutating P600 and H481 for the SynGAP–Ras/Rap interface stability. **(A)** The overview of the WT-SynGAP–Ras complex indicates the locations of Pro600 and His481 (magenta spheres). **(B)** At the complex interface, Pro600 (sticks with magenta carbons) on helix α6 maintains orientation of the helix-loop junction, while Lys492 stabilizes Ras-Glu63. The P600V is tolerated at this site, showing only minor disruption. **(C)** Also at the WT interface, His481 facilitates a stabilizing H-bond with Ras-Asp30, while the charge-reversal introduced by H481D triggers electrostatic repulsion that causes localized disengagement of the SynGAP–Ras tether. **(D)** The overview of the SynGAP–Rap complex, similarly, indicates the locations of Pro600 and His481 (magenta spheres) at the complex interface. **(E)** In contrast to the SynGAP-Ras complex, the P600V causes a substantial structural drift between the two proteins. This destabilization is primarily driven by a severe steric clash between the valine side-chain and Rap-Phe64, which pushes helix α6 away from the SynGAP–Switch II interface. **(F)** Finally, while the WT-SynGAP–Rap interface is characterized by a close π-stacking between His481 and Rap-Tyr32 (CPK models highlight the van der Waals contact), the H481D variant, eliminates the stacking and leads to a large separation between SynGAP and the Rap-Tyr32/Glu30 region. All panels show SynGAP in grey and Ras/Rap in yellow/teal, respectively, with H-bonds indicated by orange dashed lines and distances by green dashed lines. See Fig. S19 for 2D interaction diagrams.

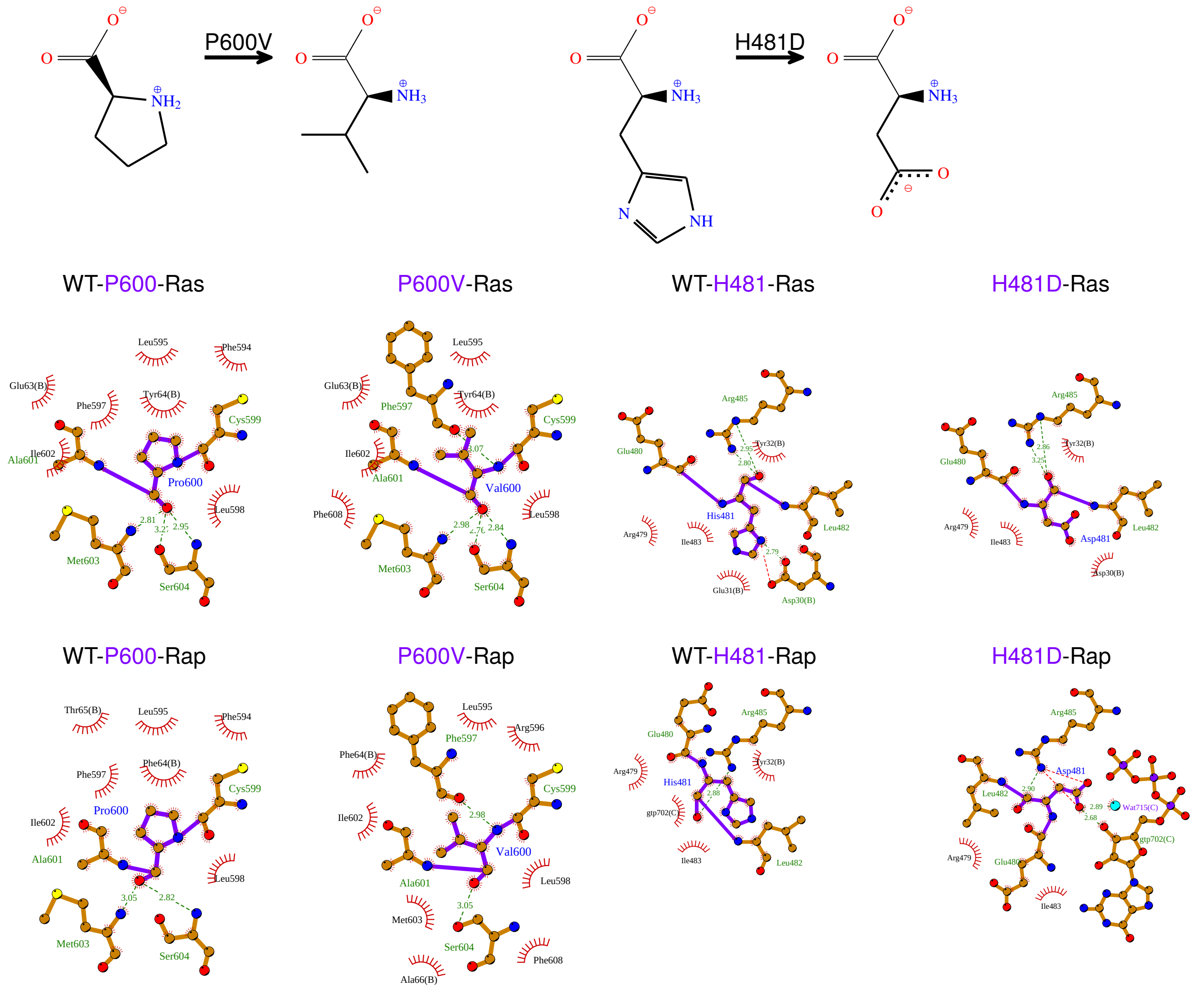

Figure S19. Interaction diagrams for the missense mutations P600V and H481D with the Ras/Rap-GTPases. The WT-Pro600/His481 and the mutated Val600/Asp481 for the missense mutations P600V and H481D are shown as 2D plots (bonds/carbons = black lines). The residues are highlighted (magenta bonds) in the WT and mutated systems as 2D interaction diagrams in complex with Ras-GTPase. Likewise, the corresponding WT and mutated residues are highlighted as interaction diagrams in complex with Rap GTPase. Coordinates for both the WT and missense variant structures are the same 100-ns MD snapshots as seen in Fig. S18. For further interpretation see Figs. S11 and S18.

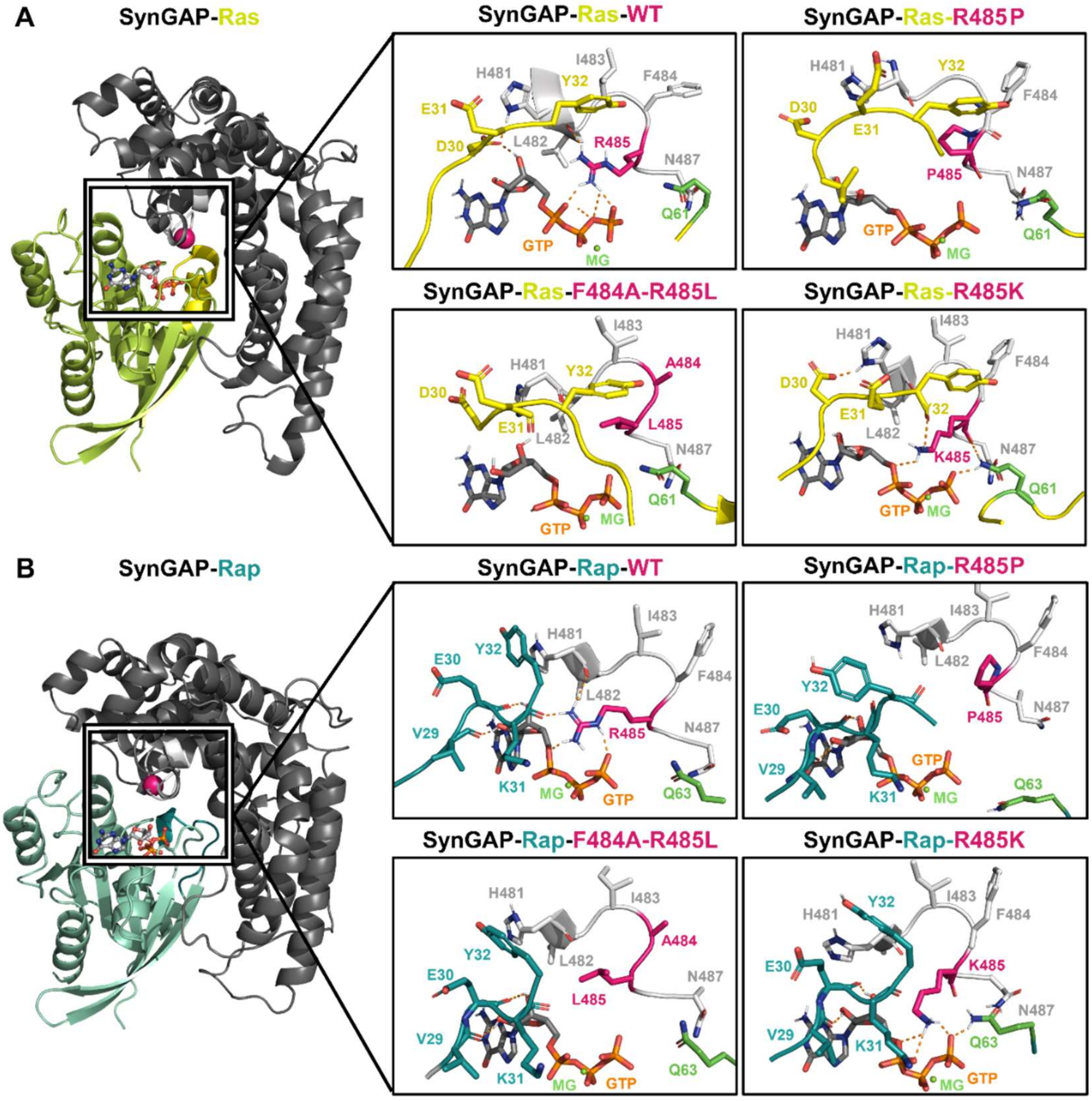

Figure S20. Impact of Arg-finger-targeting missense mutations on the catalytic machinery of Ras/Rap-GTPases. (A) The overview (on the left) shows the Arg-finger of SynGAP (Arg485; magenta sphere) inserted into the Ras-GTPase active-site. In the WT-SynGAP–Ras interface, Arg485 (sticks with magenta carbons) is precisely oriented to stabilize the negative charge of the *γ*-phosphate in the transition state GTP. The arginine acts as a critical structural anchor due to its unique size and geometry that provides steric packing for stabilizing the SynGAP–Ras interface. The R485P mutation causes a collapse of the catalytic site structure in the simulations. The F484A/R485L double mutant eliminates both the bulky hydrophobic support and the positive charge, essentially abolishing the catalytic machinery, but the Ala-Leu substitutions pack against Ras-Tyr32 to stabilize the loop within the active-site. In the R485K missense variant, while the positive charge of Arg-finger is preserved, the shorter and more flexible lysine side-chain slightly disrupts the catalytic site arrangement. **(B)** The overview (on the left) illustrates the SynGAP–Rap catalytic centre. Similar to WT-SynGAP–Ras complex, the interface of WT-SynGAP–Rap complex relies on Arg485 to neutralize the transition state of GTP and maintain steric stability within the active-site. The R485P mutation causes a similar structural disengagement in the SynGAP–Rap complex as happened with the SynGAP–Ras complex. The F484A/R485L double mutant shows a total loss of catalytic and hydrophobic anchoring, leading to a structural drift of SynGAP away from Rap. In the R485K variant, the local environment near Rap-Switch II is perturbed; the lysine substitution induces a shift in the surrounding residues causing interface misalignment. All panels show SynGAP in grey and Ras/Rap-GTPases in yellow/teal, respectively. The catalytic glutamine is shown in green sticks and the H-bonds are indicated by orange dashed lines. Key residues are shown as sticks and magnesium (MG; green sphere) as well as GTP are visible in the active-site. See Fig. S21 for 2D interaction diagrams.

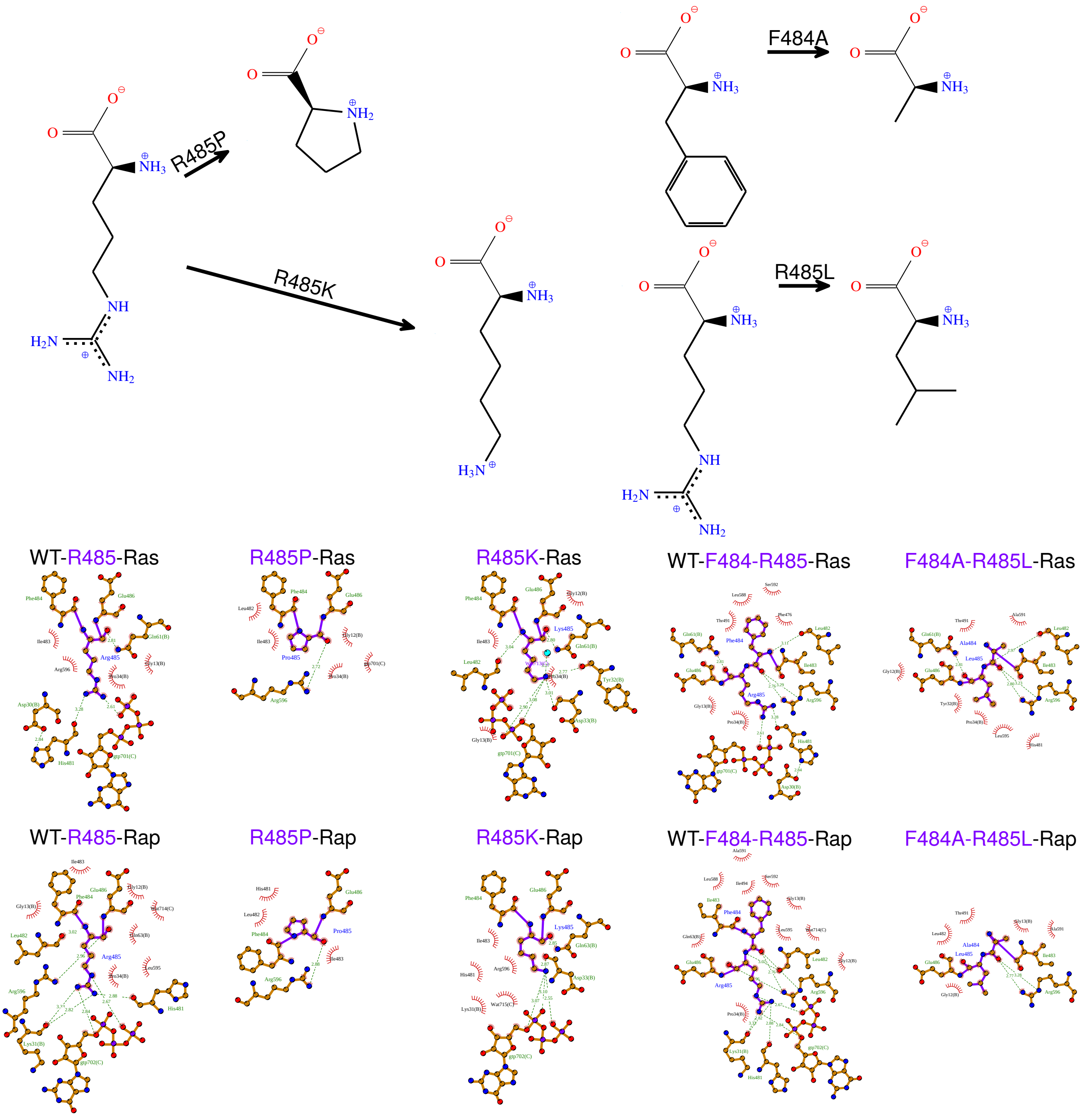

Figure S21. Interaction diagrams for the missense mutations R485P and R485K. The WT-Arg485 and the mutated Pro485/Lys485 for the missense mutations R485P and R485K and the WT-Phe484/Arg485 and the mutated Ala484-Leu485 for the F484A-R485L mutation are shown as 2D plots (bonds/carbons = black lines). The residues are highlighted (magenta bonds) in the WT and mutated systems as 2D interaction diagrams in complex with Ras-GTPase. Likewise, the corresponding WT and mutated residues are highlighted as interaction diagrams in complex with Rap GTPase. Coordinates for both the WT and missense variant structures are the same 100-ns MD snapshots as seen in Fig. S20. For further interpretation see Figs. S11 and S20.

Table S1. Binding affinities and folding stabilities for SynGAP–Ras and SynGAP–Rap GTPase variant complexes

Variant	SynGAP-Ras Complex					SynGAP-Rap Complex				
	FoldX-MD $\Delta\Delta G_{bind}$[1]	FoldX-MD $\Delta\Delta G_{bind}$[2]	Rosetta $\Delta\Delta G_{bind}$[3]	Foldx-MD $\Delta\Delta G_{fold}$[4]	Foldx-MD $\Delta\Delta G_{fold}$[5]	FoldX-MD $\Delta\Delta G_{bind}$[1]	FoldX-MD $\Delta\Delta G_{bind}$[2]	Rosetta $\Delta\Delta G_{bind}$[3]	Foldx-MD $\Delta\Delta G_{fold}$[4]	Foldx-MD $\Delta\Delta G_{fold}$[5]
N199S	0 ± 0	0 ± 0	0 ± 0	0.2 ± 0.2	0.2 ± 0.1	0 ± 0	0 ± 0	0 ± 0	0.1 ± 0.0	0.1 ± 0.1
E202K	0 ± 0	0 ± 0	0 ± 0	-0.4 ± 0.2	-0.4 ± 0.1	0 ± 0	0 ± 0	0 ± 0	-0.3 ± 0.0	-0.4 ± 0.1
S204F	0 ± 0	0 ± 0	0 ± 0.1	9.9 ± 4.4	13.2 ± 2.2	0 ± 0	0 ± 0	0.1 ± 0.1	7.8 ± 4.6	10.3 ± 0.5
I206M	0 ± 0	0 ± 0	0 ± 0	1.3 ± 0.4	2.0 ± 0.3	0 ± 0	0 ± 0	0 ± 0	1.0 ± 0.6	1.3 ± 0.0
I206T	0 ± 0	0 ± 0	0 ± 0	3.1 ± 0.2	3.2 ± 0.1	0 ± 0	0 ± 0	0 ± 0	2.9 ± 0.4	3.1 ± 0.1
S211N	0 ± 0	0 ± 0	0 ± 0	0.2 ± 0	0.3 ± 0.2	0 ± 0	0 ± 0	0 ± 0	0.2 ± 0.3	0.3 ± 0.2
S212F	0 ± 0	0 ± 0	0 ± 0	1.6 ± 0.2	1.6 ± 2.0	0 ± 0	0 ± 0	0 ± 0	1.7 ± 1.2	2.2 ± 1.7
I213M	0 ± 0	0 ± 0	0 ± 0	0.5 ± 0.1	0.5 ± 0.2	0 ± 0	0 ± 0	0 ± 0	0.7 ± 0.4	0.5 ± 0.3
E221K	0 ± 0	0 ± 0	0 ± 0	-0.7 ± 0	-0.7 ± 0.1	0 ± 0	0 ± 0	0 ± 0	-0.8 ± 0.2	-0.7 ± 0.1
T223I	0 ± 0	0 ± 0	0 ± 0	-0.6 ± 0.5	-0.6 ± 0.3	0 ± 0	0 ± 0	0 ± 0	-0.8 ± 0.4	-1.0 ± 0.0
T224A	0 ± 0	0 ± 0	0 ± 0	0.7 ± 0.1	0.6 ± 0.1	0 ± 0	0 ± 0	0 ± 0	0.6 ± 0.1	0.6 ± 0.1
S226L	0 ± 0	0 ± 0	0 ± 0	-0 ± 0.2	0.1 ± 0	0 ± 0	0 ± 0	0 ± 0	-0.1 ± 0.1	-0.2 ± 0.0
T228I	0 ± 0	0 ± 0	0 ± 0	-0 ± 0.3	-0.1 ± 0.3	0 ± 0	0 ± 0	0 ± 0	0.3 ± 0.3	0.5 ± 0.1
A232T	0 ± 0	0 ± 0	0 ± 0	0.3 ± 0.1	0.5 ± 0.2	0 ± 0	0 ± 0	0 ± 0	0.4 ± 0.1	0.4 ± 0.1
R234L	0 ± 0	0 ± 0	0 ± 0	1.0 ± 0.1	1.0 ± 0.3	0 ± 0	0 ± 0	0 ± 0	1.1 ± 0.3	1.2 ± 0.1
R234Q	0 ± 0	0 ± 0	0 ± 0	0.9 ± 0.1	1.0 ± 0.3	0 ± 0	0 ± 0	0 ± 0	1.0 ± 0.2	1.1 ± 0.1
A236S	0 ± 0	0 ± 0	0 ± 0	0.4 ± 0.1	0.4 ± 0	0 ± 0	0 ± 0	0 ± 0	0.4 ± 0.0	0.5 ± 0.0
A236V	0 ± 0	0 ± 0	0 ± 0	0.6 ± 0	0.5 ± 0.1	0 ± 0	0 ± 0	0 ± 0	0.6 ± 0.1	0.5 ± 0.0
A237S	0 ± 0	0 ± 0	0 ± 0	0.4 ± 0	0.4 ± 0.1	0 ± 0	0 ± 0	0 ± 0	0.4 ± 0.0	0.4 ± 0.1
E238K	0 ± 0	0 ± 0	0 ± 0	0.7 ± 0.1	0.8 ± 0.4	0 ± 0	0 ± 0	0 ± 0	0.8 ± 0.3	0.5 ± 0.0
K241R	0 ± 0	0 ± 0	0 ± 0	-0.2 ± 0	-0.2 ± 0.1	0 ± 0	0 ± 0	0 ± 0	-0.2 ± 0.1	-0.2 ± 0.0
E244K	0 ± 0	0 ± 0	0 ± 0	0.7 ± 0.3	0.4 ± 0.2	0 ± 0	0 ± 0	0 ± 0	0.6 ± 0.0	0.4 ± 0.3
E244Q	0 ± 0	0 ± 0	0 ± 0	0.5 ± 0.2	0.3 ± 0.2	0 ± 0	0 ± 0	0 ± 0	0.5 ± 0.1	0.1 ± 0.2
Q247L	0 ± 0	0 ± 0	0 ± 0	-0.9 ± 0	-0.9 ± 0	0 ± 0	0 ± 0	0 ± 0	-0.9 ± 0.1	-1.0 ± 0.1
R248W	0 ± 0	0 ± 0	0 ± 0	2.0 ± 0.7	1.6 ± 0.3	0 ± 0	0 ± 0	0 ± 0	1.5 ± 0.3	1.6 ± 0.3
N253S	0 ± 0	0 ± 0	0 ± 0	0.7 ± 0.1	0.8 ± 0.1	0 ± 0	0 ± 0	0 ± 0	0.7 ± 0.1	0.7 ± 0.1
K254N	0 ± 0	0 ± 0	0 ± 0	0.3 ± 0.1	0.4 ± 0.2	0 ± 0	0 ± 0	0 ± 0	0.2 ± 0.2	0.1 ± 0.2
S257I	0 ± 0	0 ± 0	0 ± 0	0.6 ± 0.1	0.4 ± 0.3	0 ± 0	0 ± 0	0 ± 0	0.6 ± 0.2	0.6 ± 0.2
R258H	0 ± 0	0 ± 0	0 ± 0	2.1 ± 0.3	1.9 ± 0.2	0 ± 0	0 ± 0	0 ± 0	1.9 ± 0.1	2.0 ± 0.3
R258S	0 ± 0	0 ± 0	0 ± 0	2.4 ± 0.3	2.4 ± 0.1	0 ± 0	0 ± 0	0 ± 0	2.2 ± 0.0	2.3 ± 0.3
R259Q	0 ± 0	0 ± 0	0 ± 0	2.6 ± 0.2	3.2 ± 0.4	0 ± 0	0 ± 0	0 ± 0	2.8 ± 0.7	2.5 ± 0.9
R259W	0 ± 0	0 ± 0	0 ± 0	2.6 ± 0.3	3.2 ± 0.4	0 ± 0	0 ± 0	0.1 ± 0.1	2.8 ± 1.2	2.3 ± 0.8
D261H	0 ± 0	0 ± 0	0 ± 0	2.6 ± 0.7	1.8 ± 0.2	0 ± 0	0 ± 0	0 ± 0	1.9 ± 0.8	2.7 ± 0.3
W267L	0.1 ± 0.1	0 ± 0.1	0.2 ± 0.2	2.0 ± 0.6	2.1 ± 0.7	0 ± 0	0.1 ± 0.2	-0.1 ± 0	1.3 ± 0.3	1.9 ± 0.5
R272W	0.5 ± 0.4	1.0 ± 0.5	1 ± 0.3	4.4 ± 0.7	3.6 ± 0.1	0.6 ± 0.1	0.8 ± 0.1	0.6 ± 0.3	3.0 ± 0.3	3.5 ± 0.1
P276H	0 ± 0	0 ± 0	0 ± 0	2.1 ± 0.1	2.1 ± 0	0 ± 0	0 ± 0	0 ± 0	2.1 ± 0.0	2.1 ± 0.2
K277N	0 ± 0	0 ± 0	0 ± 0	-0 ± 0.1	-0.1 ± 0.1	0 ± 0	0 ± 0	0 ± 0	-0.0 ± 0.1	0.0 ± 0.1
R279L	0 ± 0	0 ± 0	0 ± 0	0.3 ± 0.1	0.3 ± 0.1	0 ± 0	0 ± 0	0 ± 0.1	0.4 ± 0.1	0.5 ± 0.4
R279Q	0 ± 0	0 ± 0	0 ± 0	1.2 ± 0.2	1.2 ± 0.1	0 ± 0	0 ± 0	0 ± 0	1.4 ± 0.2	1.9 ± 0.4
L284F	0 ± 0	0 ± 0	0 ± 0	1.7 ± 0.2	2.3 ± 0.2	0 ± 0	0 ± 0	0.1 ± 0	2.4 ± 0.5	2.8 ± 0.6
D287G	0 ± 0	0 ± 0	0 ± 0	1.2 ± 0.4	1.3 ± 0.7	0 ± 0	0 ± 0	0 ± 0	1.6 ± 0.4	1.4 ± 0.6
Y291S	0 ± 0	0 ± 0	0 ± 0	2.5 ± 0.2	2.3 ± 0.3	0 ± 0	0 ± 0	0 ± 0	2.7 ± 1.1	2.3 ± 0.5
R293C	0 ± 0	0 ± 0	0 ± 0	2.7 ± 0.1	3.1 ± 0.4	0 ± 0	0 ± 0	0 ± 0	2.8 ± 0.1	2.8 ± 0.1
R293H	0 ± 0	0 ± 0	0 ± 0	4.5 ± 0.8	3.3 ± 0.1	0 ± 0	0 ± 0	0 ± 0	4.3 ± 0.6	4.0 ± 1.2
S296F	0 ± 0	0 ± 0	0 ± 0	0.8 ± 0	1.2 ± 0.2	0 ± 0	0 ± 0	0 ± 0	1.1 ± 0.1	1.4 ± 0.4
R299H	0 ± 0	-0.1 ± 0	0 ± 0	4.3 ± 0	4.3 ± 0.5	0 ± 0	0 ± 0	0 ± 0	3.9 ± 0.2	4.0 ± 0.2
S300C	0 ± 0	0 ± 0	0 ± 0	0.7 ± 0.4	0.5 ± 0.1	0 ± 0	0 ± 0	0 ± 0	0.5 ± 0.1	0.6 ± 0.1
A301T	0 ± 0	0 ± 0	0 ± 0	0.3 ± 0.2	0.3 ± 0.2	0 ± 0	0 ± 0	0 ± 0	0.3 ± 0.0	0.3 ± 0.0
S302A	0 ± 0	0 ± 0	0 ± 0	0.4 ± 0.2	0.2 ± 0.1	0 ± 0	0 ± 0	0 ± 0.1	0.1 ± 0.0	0.2 ± 0.1
S302C	0 ± 0	0 ± 0	0 ± 0	0.4 ± 0.2	0.2 ± 0.1	0 ± 0	0 ± 0	-0.1 ± 0.1	0.2 ± 0.0	0.3 ± 0.1
S302F	0 ± 0	0 ± 0	0 ± 0	0.2 ± 0.3	-0.1 ± 0.1	0 ± 0	-0.1 ± 0.1	-0.2 ± 0.4	-0.1 ± 0.0	-0.2 ± 0.1
T305I	0 ± 0	0 ± 0	0 ± 0	0.3 ± 0.1	0.3 ± 0.5	0 ± 0.1	-0.1 ± 0	0.6 ± 0.9	0.5 ± 0.1	0.5 ± 0.1
V306I	0 ± 0	0 ± 0	0 ± 0	0.6 ± 0.5	1.1 ± 0.2	0 ± 0	0 ± 0	0 ± 0	1.1 ± 0.1	1.2 ± 0.4
W308R	0 ± 0.1	0.1 ± 0.1	0 ± 0	5.6 ± 0.2	6.0 ± 0	-0.1 ± 0	-0.1 ± 0.1	0 ± 0	5.7 ± 0.2	5.8 ± 0.2
E310K	0 ± 0	0 ± 0	0 ± 0	0.7 ± 0.4	1.1 ± 0.7	0 ± 0	0 ± 0	0 ± 0	1.9 ± 1.0	2.6 ± 0.2
N316K	0 ± 0	0 ± 0	0 ± 0	0.9 ± 0	0.5 ± 0.4	0 ± 0	0 ± 0	0 ± 0	1.0 ± 0.2	1.0 ± 0.3
R321H	0 ± 0	0 ± 0	0 ± 0	0.9 ± 0	0.7 ± 0.1	0 ± 0	0 ± 0	0 ± 0	0.9 ± 0.0	0.8 ± 0.0
A322T	0 ± 0	0 ± 0	0 ± 0	0.7 ± 0.3	0.7 ± 0.1	0 ± 0	0 ± 0	0 ± 0	0.4 ± 0.2	0.5 ± 0.0
A322V	0 ± 0	0 ± 0	0 ± 0	0.2 ± 0.3	0.1 ± 0.1	0 ± 0	0 ± 0	0 ± 0.1	0.0 ± 0.1	-0.0 ± 0.3
R324Q	0 ± 0	0 ± 0	0 ± 0	1.1 ± 0	1.1 ± 0.1	0 ± 0	0 ± 0	0 ± 0	1.1 ± 0.2	1.4 ± 0.1
R324W	0 ± 0	0 ± 0	0 ± 0	1.3 ± 0.1	1.6 ± 0.1	0 ± 0	0 ± 0	0 ± 0	1.6 ± 0.5	2.0 ± 0.1
R329C	0 ± 0	0 ± 0	0 ± 0	1.2 ± 0.4	1.2 ± 0.3	0 ± 0	0 ± 0	0 ± 0	1.1 ± 0.2	1.1 ± 0.1
R329H	0 ± 0	0 ± 0	0 ± 0	1.8 ± 0.6	1.8 ± 0.4	0 ± 0	0 ± 0	0 ± 0	2.1 ± 0.5	2.0 ± 0.2
R335C	0 ± 0	0 ± 0	0 ± 0	1.2 ± 0.2	1.1 ± 0.3	0 ± 0	0 ± 0	0 ± 0	1.1 ± 0.2	1.2 ± 0.3
R335H	0 ± 0	0 ± 0	0 ± 0	1.0 ± 0.2	0.8 ± 0.2	0 ± 0	0 ± 0	0 ± 0	1.0 ± 0.1	1.0 ± 0.3
D338H	0 ± 0	0 ± 0	0 ± 0	1.7 ± 0.6	1.4 ± 0.5	0 ± 0	0 ± 0	0 ± 0	1.3 ± 0.2	1.6 ± 0.5
D338Y	0 ± 0	0 ± 0	0 ± 0	1.3 ± 0.5	1.2 ± 0.4	0 ± 0	0 ± 0	0 ± 0	0.9 ± 0.3	1.3 ± 0.4
G341C	0 ± 0	0 ± 0	0 ± 0	0 ± 0.1	1.3 ± 1.0	0 ± 0	0 ± 0	0 ± 0	0.2 ± 0.3	0.1 ± 0.2
G344C	0 ± 0	0 ± 0	0 ± 0.1	6.9 ± 0.7	7.8 ± 0.2	0 ± 0	0 ± 0	-0.1 ± 0.2	6.8 ± 1.7	6.9 ± 1.3
G344S	0 ± 0	0 ± 0	0 ± 0	7.4 ± 0.7	8.2 ± 0.1	0 ± 0	0 ± 0	-0.1 ± 0.2	7.3 ± 1.7	7.4 ± 1.2
P349L	0 ± 0	0 ± 0	0 ± 0	0.9 ± 0.2	0.9 ± 0	0 ± 0	0 ± 0	0 ± 0	0.7 ± 0.2	0.9 ± 0.1
T352I	0 ± 0	0 ± 0	0 ± 0	-0.3 ± 0.1	-0.3 ± 0.1	0 ± 0	0 ± 0	0 ± 0	-0.4 ± 0.1	-0.3 ± 0.2
L353V	0 ± 0	0 ± 0	0 ± 0	3.2 ± 0.1	3.3 ± 0.3	0 ± 0	0 ± 0	0 ± 0	3.2 ± 0.2	3.0 ± 0.2
A354T	0 ± 0	0 ± 0	0 ± 0	0.4 ± 0.1	0.4 ± 0	0 ± 0	0 ± 0	0 ± 0	0.3 ± 0.1	0.8 ± 0.8
G355R	0 ± 0	-0.1 ± 0.1	0 ± 0	0.6 ± 0.2	0.8 ± 0.4	-0.1 ± 0.1	-0.1 ± 0.1	0 ± 0	0.2 ± 0.5	1.2 ± 0.4
R356C	0.1 ± 0	0.1 ± 0	0 ± 0	1.4 ± 0.2	1.6 ± 0.3	0.1 ± 0.1	0.1 ± 0	0 ± 0.1	1.5 ± 0.2	1.6 ± 0.4
R356H	0.1 ± 0	0.1 ± 0	0 ± 0	1.3 ± 0.3	1.3 ± 0.2	0.1 ± 0.1	0.1 ± 0	0 ± 0	1.4 ± 0.2	1.5 ± 0.6
E360K	0.3 ± 0.5	0.8 ± 0.1	-0.4 ± 1	2.1 ± 0.6	1.9 ± 0.2	1.0 ± 0.4	1.1 ± 0.6	0.3 ± 0.3	2.4 ± 0.2	2.8 ± 0.7
W362R	0.1 ± 0	0.4 ± 0.5	0 ± 0.2	4.4 ± 0.2	4.4 ± 0.5	-0.3 ± 0.5	-0.3 ± 0.5	0 ± 0.4	3.8 ± 0.4	4.1 ± 0.4
Y363S	0 ± 0	0 ± 0.1	0 ± 0	3.4 ± 0.2	3.3 ± 0.1	0 ± 0	0 ± 0	0 ± 0	3.2 ± 0.1	3.3 ± 0.2
P364S	0 ± 0	0 ± 0	0 ± 0	1.5 ± 0.2	1.3 ± 0.1	0 ± 0	0 ± 0	0 ± 0	1.3 ± 0.3	1.1 ± 0.1

Table S1. Continues

Variant	SynGAP-Ras Complex					SynGAP-Rap Complex				
	FoldX-MD $\Delta\Delta G_{bind}$[1]	FoldX-MD $\Delta\Delta G_{bind}$[2]	Rosetta $\Delta\Delta G_{bind}$[3]	Foldx-MD $\Delta\Delta G_{fold}$[4]	Foldx-MD $\Delta\Delta G_{fold}$[5]	FoldX-MD $\Delta\Delta G_{bind}$[1]	FoldX-MD $\Delta\Delta G_{bind}$[2]	Rosetta $\Delta\Delta G_{bind}$[3]	Foldx-MD $\Delta\Delta G_{fold}$[4]	Foldx-MD $\Delta\Delta G_{fold}$[5]
T366A	0 ± 0	0 ± 0	0 ± 0	0.1 ± 0	0.1 ± 0.1	0 ± 0	0 ± 0	0 ± 0	0.0 ± 0.1	0.1 ± 0.1
T369I	0 ± 0	0 ± 0	0 ± 0	0.2 ± 0	0.5 ± 0.2	0 ± 0	0 ± 0	0 ± 0	0.4 ± 0.1	0.4 ± 0.1
S371N	0 ± 0	0 ± 0	0 ± 0	-0 ± 0	-0 ± 0.1	0 ± 0	0 ± 0	0 ± 0	-0.1 ± 0.1	0.0 ± 0.1
G375V	0 ± 0	0 ± 0	0 ± 0	9.4 ± 2.6	8.6 ± 2.4	0 ± 0	0 ± 0	0 ± 0	5.5 ± 1.3	5.3 ± 2.1
M377I	0 ± 0	0 ± 0	0 ± 0	1.1 ± 0.1	0.7 ± 0.1	0 ± 0	0 ± 0	0 ± 0	0.5 ± 0.2	0.6 ± 0.2
S379L	0 ± 0	0 ± 0	0 ± 0	0.4 ± 0.4	0 ± 0.3	0 ± 0	0 ± 0	0 ± 0	0.3 ± 0.1	0.4 ± 0.1
G380E	0 ± 0	0 ± 0	0 ± 0	5.0 ± 0.1	5.0 ± 1.0	0 ± 0	0 ± 0	0 ± 0	4.1 ± 1.3	4.9 ± 0.5
G381V	0 ± 0	0 ± 0	0 ± 0	5.5 ± 0	4.3 ± 0.9	0 ± 0	0 ± 0	0 ± 0	5.8 ± 1.7	5.5 ± 1.0
G382E	0 ± 0	0 ± 0	0 ± 0	4.3 ± 0.8	5.1 ± 0.6	0 ± 0	0 ± 0	0 ± 0	3.6 ± 0.6	4.6 ± 0.7
G384C	0 ± 0	0 ± 0	0 ± 0	2.2 ± 0.3	2.0 ± 0.3	0 ± 0	0 ± 0	0 ± 0	2.5 ± 0.2	2.8 ± 0.5
S385W	0 ± 0	0 ± 0	0 ± 0	0.8 ± 0.7	0.6 ± 0.2	0 ± 0	0 ± 0	0 ± 0	0.6 ± 0.3	0.8 ± 0.4
G388D	0.2 ± 0.2	0.2 ± 0.1	0.1 ± 0.3	2.3 ± 0.4	3.8 ± 1.4	0 ± 0	0.1 ± 0.1	0 ± 0	2.8 ± 0.4	1.8 ± 0.5
G395R	-0.1 ± 0.2	-0.1 ± 0.2	-0.1 ± 0.1	1.8 ± 0.3	1.7 ± 0.3	-0.1 ± 0.1	-0.1 ± 0	0 ± 0	1.9 ± 0.9	2.0 ± 0.3
G396S	0 ± 0	0 ± 0	0 ± 0	2.9 ± 1.0	1.7 ± 0.3	0 ± 0	0 ± 0	0 ± 0	1.8 ± 0.2	2.4 ± 0.5
P398L	0 ± 0	0 ± 0	0 ± 0.1	0.7 ± 0.4	1.1 ± 0.6	0 ± 0	0 ± 0	0 ± 0.1	1.0 ± 0.1	1.2 ± 0.2
R401Q	1.4 ± 1.9	1.8 ± 0.1	0.4 ± 0.5	3.1 ± 1.6	2.8 ± 0.1	3.5 ± 0.7	4.1 ± 1.2	1.3 ± 0.4	4.4 ± 0.6	5.3 ± 1.4
R401W	1.0 ± 1.6	1.1 ± 0.1	-0.1 ± 0.4	3.3 ± 1.5	3.3 ± 0.5	2.7 ± 0.5	3.2 ± 1.3	0.6 ± 0.3	4.1 ± 0.4	5.1 ± 1.7
R405H	0.1 ± 0	0.1 ± 0	0 ± 0	4.1 ± 0.5	3.9 ± 0.5	0.1 ± 0.1	0.1 ± 0	0 ± 0	5.4 ± 0.7	3.1 ± 0.7
M409I	0 ± 0	0 ± 0	0 ± 0	0.9 ± 0.3	0.9 ± 0.1	0 ± 0	0 ± 0	0 ± 0	0.9 ± 0.3	1.0 ± 0.1
L412F	0 ± 0	0 ± 0	0 ± 0	6.1 ± 0.6	6.5 ± 0.3	0 ± 0	0 ± 0	0 ± 0	6.6 ± 0.4	6.7 ± 0.4
R429Q	0 ± 0	0 ± 0	0 ± 0	0.7 ± 0	0.6 ± 0.1	0 ± 0	0 ± 0	0 ± 0	0.7 ± 0.0	0.6 ± 0.0
R429W	0 ± 0	0 ± 0	0 ± 0	0.5 ± 0.1	0.5 ± 0.1	0 ± 0	0 ± 0	0 ± 0	0.6 ± 0.2	0.4 ± 0.1
M430T	0 ± 0	0 ± 0	0 ± 0	1.6 ± 0.1	1.7 ± 0	0 ± 0	0 ± 0	0 ± 0	1.7 ± 0.1	1.7 ± 0.1
L431M	0 ± 0	0 ± 0	0 ± 0	0.2 ± 0	0.2 ± 0	0 ± 0	0 ± 0	0 ± 0	0.2 ± 0.0	0.2 ± 0.0
A433T	0 ± 0	0 ± 0	0 ± 0	0.4 ± 0.1	0.4 ± 0	0 ± 0	0 ± 0	0 ± 0	0.3 ± 0.1	0.4 ± 0.0
A438T	0 ± 0	0 ± 0	0 ± 0	0.3 ± 0.1	0.3 ± 0.1	0 ± 0	0 ± 0	0 ± 0	0.3 ± 0.1	0.2 ± 0.1
A438V	0 ± 0	0 ± 0	0 ± 0	-0.1 ± 0.2	-0.1 ± 0.1	0 ± 0	0 ± 0	0 ± 0	-0.3 ± 0.2	-0.3 ± 0.1
N440I	-0.1 ± 0.1	0.1 ± 0.2	-0.5 ± 0.1	1.0 ± 0.2	1.1 ± 0.2	0.3 ± 0.2	0.7 ± 0.3	0 ± 0.1	1.3 ± 0.1	1.9 ± 0.4
V441I	0 ± 0.1	0 ± 0.1	0 ± 0.3	-0.2 ± 0	-0.4 ± 0.2	-0.1 ± 0	0.1 ± 0.1	0.2 ± 0.2	-0.2 ± 0.1	0.0 ± 0.2
K442R	-0.5 ± 0.2	-0.7 ± 0.1	0 ± 0.1	-0.3 ± 0.1	-0.5 ± 0.1	-0.8 ± 0.2	-1.4 ± 0.1	0.5 ± 0.3	-0.6 ± 0.2	-1.2 ± 0.1
L451I	0 ± 0	0 ± 0	0 ± 0	1.2 ± 0.1	1.3 ± 0.1	0 ± 0	0 ± 0	0 ± 0	1.3 ± 0.2	1.6 ± 0.1
H453Y	0 ± 0	0.1 ± 0	0 ± 0	-0.4 ± 0.1	-0.3 ± 0.1	0 ± 0	0 ± 0	0 ± 0	-0.6 ± 0.2	-0.5 ± 0.2
K460N	0 ± 0	0 ± 0	0 ± 0	0.3 ± 0.1	0.3 ± 0.1	0 ± 0	0 ± 0	0 ± 0	0.3 ± 0.0	0.3 ± 0.0
A461D	0 ± 0	0 ± 0	0 ± 0	1.5 ± 0.4	1.6 ± 0.1	0 ± 0	0 ± 0	0 ± 0	1.7 ± 0.3	1.6 ± 0.3
S466L	0 ± 0	0 ± 0	0 ± 0	-0 ± 0.3	-0.1 ± 0.2	0 ± 0	0 ± 0	0 ± 0	0.2 ± 0.4	-0.0 ± 0.1
D467G	0 ± 0	0 ± 0	0 ± 0	2.2 ± 0	2.1 ± 0.3	0 ± 0	0 ± 0	0 ± 0	2.1 ± 0.3	2.2 ± 0.1
M468I	0 ± 0	0 ± 0	0 ± 0	1.5 ± 0.6	1.1 ± 0.1	0 ± 0	0 ± 0	0 ± 0	1.6 ± 0.9	2.7 ± 0.1
M470I	0 ± 0	0 ± 0	0 ± 0	1.4 ± 0.1	1.3 ± 0.1	0 ± 0	0 ± 0	0 ± 0	1.5 ± 0.1	1.5 ± 0.1
E472K	0.1 ± 0.1	0.1 ± 0.1	0 ± 0	2.1 ± 0.5	1.2 ± 0.7	-0.1 ± 0.1	-0.3 ± 0.1	0 ± 0	1.5 ± 0.5	1.2 ± 0.3
V473I	0 ± 0	0 ± 0	0 ± 0	-0.3 ± 0.1	-0.2 ± 0.1	0 ± 0	0 ± 0	0 ± 0	-0.2 ± 0.2	-0.0 ± 0.0
D474Y	0 ± 0	0 ± 0	0 ± 0	0.5 ± 0.2	0.6 ± 0.1	0 ± 0	-0.1 ± 0	0 ± 0	0.4 ± 0.3	0.3 ± 0.1
R475G	-0.1 ± 0.1	-0.1 ± 0	-0.1 ± 0.1	2.6 ± 0.7	2.6 ± 0.3	0.3 ± 0.2	0.6 ± 0.2	0 ± 0.1	2.7 ± 0.5	2.6 ± 0.2
R475W	-0.1 ± 0.1	-0.2 ± 0	0 ± 0.1	2.0 ± 1.0	2.0 ± 0.5	0.3 ± 0.1	0.5 ± 0.2	0 ± 0	1.7 ± 0.4	1.6 ± 0.1
F476S	0.1 ± 0.1	0.3 ± 0.1	0 ± 0.1	3.1 ± 0.3	3.5 ± 0.1	0.1 ± 0.1	0.2 ± 0	-0.1 ± 0.1	2.9 ± 0.3	3.1 ± 0.2
R479Q	0.2 ± 0.3	0.4 ± 0.1	0 ± 0.1	1.7 ± 0.8	2.5 ± 0.6	1.7 ± 0.1	1.9 ± 0.2	0.2 ± 0.2	2.2 ± 0.2	2.2 ± 0.1
R479W	-0.1 ± 0.4	0.1 ± 0.2	-0.1 ± 0.1	1.3 ± 0.8	1.5 ± 0.5	1.5 ± 0.1	1.5 ± 0.2	0.1 ± 0.1	1.7 ± 0.3	1.7 ± 0.1
H481D	0.9 ± 0.3	1.2 ± 0.1	0.6 ± 0.2	0.6 ± 0.1	1.5 ± 0.1	0.9 ± 0.4	0.8 ± 0.1	0.7 ± 0.8	0.8 ± 0.0	1.5 ± 0.1
F484A	0 ± 0	0.1 ± 0	0 ± 0	4.4 ± 0.2	4.2 ± 0.2	0 ± 0	0 ± 0	0 ± 0.1	4.3 ± 0.4	4.8 ± 0.1
R485L-F484A	0.1 ± 0.1	-0.2 ± 0.3	-0.8 ± 0.1	4.5 ± 0.4	7.1 ± 0.5	0.2 ± 0.2	-0.4 ± 0.3	0.1 ± 0.4	4.3 ± 0.4	8.3 ± 0.2
R485L	0.1 ± 0.1	-0.3 ± 0.3	-0.7 ± 0.2	-0 ± 0.2	2.9 ± 0.4	0.1 ± 0.3	-0.5 ± 0.2	-0.1 ± 0.5	-0.0 ± 0.3	3.4 ± 0.1
R485K	0.7 ± 0.5	0.7 ± 0.2	0.8 ± 0.2	1.1 ± 0.5	2.9 ± 0.2	0.6 ± 0.4	0.3 ± 0.3	0.7 ± 0.4	0.8 ± 0.4	3.1 ± 0.2
R485H	1.2 ± 1.0	2.7 ± 0.9	0.2 ± 0.5	2.2 ± 0.8	6.6 ± 1.1	0.9 ± 0.4	0.5 ± 0.2	0.1 ± 0.4	1.6 ± 0.2	4.7 ± 0.4
R485P	1.8 ± 0.6	2.4 ± 0.3	1.1 ± 0.9	6.4 ± 1.7	9.2 ± 0.4	1.3 ± 0.5	0.7 ± 0.2	0.8 ± 0.4	4.9 ± 1.9	9.1 ± 0.4
E486K	0.5 ± 0.6	1.3 ± 0.1	0.3 ± 0.2	1.4 ± 1.2	1.8 ± 0.4	0.1 ± 0.2	0.5 ± 0.2	0 ± 0.3	0.6 ± 0.2	0.5 ± 0.2
N487K	0.8 ± 0.8	0.7 ± 0.8	0.1 ± 0.6	2.6 ± 1.4	1.9 ± 0.7	0.1 ± 0.1	0.9 ± 0.4	0.5 ± 1.4	1.4 ± 0.5	2.5 ± 0.8
N487T	1.2 ± 0.5	1.0 ± 0.7	-0.3 ± 0.2	3.1 ± 1.0	2.3 ± 0.7	0.2 ± 0.4	0.6 ± 0.2	-0.1 ± 0.1	1.8 ± 0.6	3.1 ± 0.5
T488M	-0.1 ± 0.1	0 ± 0.1	0.1 ± 0.1	2.7 ± 0.6	3.4 ± 0.9	-0.1 ± 0.1	-0.1 ± 0	0 ± 0	2.1 ± 0.5	1.8 ± 0.1
T491I	0 ± 0.1	0.1 ± 0.1	0.2 ± 0.1	1.8 ± 0.2	2.9 ± 0.6	-0.1 ± 0.1	0 ± 0	-0.1 ± 0.1	1.4 ± 0.1	1.8 ± 0.4
T491S	0.1 ± 0.1	0.1 ± 0	-0.1 ± 0.1	1.3 ± 0.1	1.4 ± 0.2	0 ± 0	0.1 ± 0	0 ± 0	1.1 ± 0.2	1.2 ± 0.1
K492E	1.6 ± 0.5	1.7 ± 0.9	1 ± 0.2	3.8 ± 0.2	4.3 ± 0.8	1.1 ± 0.9	1.7 ± 0.3	0.5 ± 0.3	3.1 ± 0.7	3.7 ± 0.2
A493D	0.1 ± 0	0.1 ± 0	0 ± 0	3.6 ± 0.3	3.6 ± 0.8	0 ± 0	0 ± 0	0 ± 0	4.5 ± 0.6	4.3 ± 0.4
A493V	0 ± 0	0 ± 0	0 ± 0	0.4 ± 0.5	0.2 ± 0.3	0 ± 0	0 ± 0	0 ± 0	0.7 ± 0.3	0.7 ± 0.1
I494T	0 ± 0	0 ± 0	-0.1 ± 0.1	2.5 ± 0.1	2.8 ± 0.2	0 ± 0	0 ± 0	0 ± 0.1	2.5 ± 0.1	2.4 ± 0.1
Y505C	0 ± 0	0 ± 0	0 ± 0	3.6 ± 0.2	3.6 ± 0.1	0 ± 0	0 ± 0	0 ± 0	3.4 ± 0.1	3.5 ± 0.2
L506I	0 ± 0	0 ± 0	0 ± 0	1.5 ± 0.6	2.1 ± 0.2	0 ± 0	0 ± 0	0.1 ± 0.1	1.8 ± 0.3	2.0 ± 0.2
D508H	0 ± 0	0 ± 0	0 ± 0	0.3 ± 0.3	0.2 ± 0.2	0 ± 0	0 ± 0	0 ± 0	0.2 ± 0.1	0.4 ± 0.4
A509D	0 ± 0	0 ± 0	0 ± 0	2.9 ± 1.4	3.7 ± 0.2	0 ± 0	0 ± 0	0 ± 0	3.4 ± 0.5	3.9 ± 0.5
R515C	0 ± 0	0 ± 0	0 ± 0	2.1 ± 0.1	2.5 ± 0.9	0 ± 0	0 ± 0	0 ± 0	2.1 ± 0.1	2.1 ± 0.1
R515H	0 ± 0	0 ± 0	0 ± 0	1.7 ± 0.1	2.2 ± 0.9	0 ± 0	0 ± 0	0 ± 0	1.8 ± 0.1	1.8 ± 0.1
Y518C	0 ± 0	0 ± 0	0 ± 0	3.0 ± 0.5	3.2 ± 1.0	0 ± 0	0 ± 0	0 ± 0	3.3 ± 0.3	3.6 ± 0.6
C531R	0 ± 0	0 ± 0	0 ± 0	2.1 ± 1.0	1.8 ± 0.4	0 ± 0	0 ± 0	0 ± 0	0.9 ± 0.4	0.6 ± 0.7
C531Y	0 ± 0	-0.1 ± 0	0 ± 0	10.5 ± 3.1	10.6 ± 1.5	0 ± 0.1	0 ± 0	0 ± 0	6.3 ± 2.6	3.3 ± 1.6
R544L	0 ± 0	0 ± 0	0 ± 0	0.1 ± 0.7	-0.3 ± 0.1	0 ± 0	0 ± 0	0 ± 0	-0.1 ± 0.3	-0.2 ± 0.3
V554M	0 ± 0	0 ± 0	0 ± 0	-0.8 ± 0.2	-0.6 ± 0.2	0 ± 0	0 ± 0	0 ± 0	-0.7 ± 0.3	-0.7 ± 0.2
S557F	0 ± 0	0 ± 0	0 ± 0	11.6 ± 4.8	13.7 ± 1.8	0 ± 0	0 ± 0	0 ± 0	12.7 ± 1.3	14.6 ± 3.2
H558R	0 ± 0	0 ± 0	0 ± 0	-0.8 ± 0.1	-0.9 ± 0.1	0 ± 0	0 ± 0	0 ± 0	-0.6 ± 0.3	-0.1 ± 0.2
K566N	0 ± 0	0 ± 0	0 ± 0	2.4 ± 0.3	3.1 ± 0.1	0 ± 0	0 ± 0	0 ± 0	2.3 ± 0.2	2.3 ± 0.1
S571L	0 ± 0	0 ± 0	0 ± 0	-1.3 ± 0	-1.3 ± 0.1	0 ± 0	0 ± 0	0 ± 0	-1.4 ± 0.2	-1.4 ± 0.0
R573L	0 ± 0	0 ± 0	0 ± 0	0.9 ± 0.4	1.2 ± 0.4	0 ± 0	0 ± 0	0 ± 0	1.4 ± 0.9	0.7 ± 0.3
R573W	0 ± 0	0 ± 0	0 ± 0	1.7 ± 0.6	1.7 ± 0.2	0 ± 0	0 ± 0	0 ± 0	2.0 ± 0.7	1.7 ± 0.4
L574M	0 ± 0	0 ± 0	0 ± 0	0.2 ± 0	0.1 ± 0	0 ± 0	0 ± 0	0 ± 0	0.2 ± 0.0	0.2 ± 0.0

Table S1. Continues

Variant	SynGAP-Ras Complex					SynGAP-Rap Complex				
	FoldX-MD $\Delta\Delta G_{bind}$ [1]	FoldX-MD $\Delta\Delta G_{bind}$ [2]	Rosetta $\Delta\Delta G_{bind}$ [3]	Foldx-MD $\Delta\Delta G_{fold}$ [4]	Foldx-MD $\Delta\Delta G_{fold}$ [5]	FoldX-MD $\Delta\Delta G_{bind}$ [1]	FoldX-MD $\Delta\Delta G_{bind}$ [2]	Rosetta $\Delta\Delta G_{bind}$ [3]	Foldx-MD $\Delta\Delta G_{fold}$ [4]	Foldx-MD $\Delta\Delta G_{fold}$ [5]
R575H	0 ± 0	0 ± 0	0 ± 0	1.1 ± 0.1	1.1 ± 0.1	0 ± 0	0 ± 0	0 ± 0	1.2 ± 0.0	1.3 ± 0.0
R581Q	0.1 ± 0	0.1 ± 0	0 ± 0	3.6 ± 0.8	3.8 ± 0.4	0.1 ± 0.1	0.1 ± 0	0 ± 0	3.6 ± 0.2	3.2 ± 0.8
R581W	0.1 ± 0	0.1 ± 0	0 ± 0	3.1 ± 0.7	3.3 ± 0.3	0.1 ± 0.1	0.1 ± 0	0 ± 0	3.1 ± 0.1	2.7 ± 0.6
A585V	0 ± 0	0 ± 0	0 ± 0	1.1 ± 0.4	2.1 ± 0.3	0 ± 0	0 ± 0	0 ± 0	0.9 ± 0.4	1.0 ± 0.2
D586N	0 ± 0	0 ± 0	0 ± 0	0.2 ± 0.1	0.1 ± 0.1	0 ± 0.1	0 ± 0	0 ± 0	0.3 ± 0.0	0.2 ± 0.1
L588I	0 ± 0	0 ± 0	0 ± 0	2.3 ± 0.6	1.9 ± 0.2	0 ± 0	0 ± 0	0 ± 0	2.3 ± 0.3	2.6 ± 0.3
I589F	0 ± 0	0 ± 0	0 ± 0	5.0 ± 1.3	7.8 ± 0.6	0 ± 0	0 ± 0	0 ± 0	5.5 ± 0.8	5.1 ± 0.4
I589L	0 ± 0	0 ± 0	0 ± 0	0.6 ± 0.3	1.0 ± 0.2	0 ± 0	0 ± 0	0 ± 0.1	0.6 ± 0.1	0.5 ± 0.1
I589M	0 ± 0	0 ± 0	0 ± 0	0.8 ± 0.2	1.2 ± 0.1	0 ± 0	0 ± 0	0 ± 0	0.9 ± 0.1	1.0 ± 0.1
A591T	0 ± 0.1	0.3 ± 0.2	-0.1 ± 0.1	1.1 ± 0.3	1.6 ± 0.3	-0.1 ± 0.1	0 ± 0	-0.1 ± 0.1	1.0 ± 0.2	1.3 ± 0.3
R596H	1.2 ± 0.4	1.5 ± 0.3	-0.1 ± 0.3	4.8 ± 0.5	5.0 ± 0.5	1.1 ± 0.4	1.2 ± 0.3	0.5 ± 0.3	3.9 ± 1.0	5.7 ± 0.5
P600V	0.5 ± 0.7	1.4 ± 0.7	-0.2 ± 0.2	2.9 ± 1.0	4.0 ± 0.7	0.7 ± 0.4	0.7 ± 0.4	0.2 ± 0.3	3.2 ± 0.6	3.0 ± 0.8
A601V	0 ± 0	0.1 ± 0	0 ± 0.1	2.1 ± 0.1	1.9 ± 0.6	0 ± 0	0.1 ± 0	0 ± 0.1	1.5 ± 0.3	1.5 ± 0.5
M603I	1.0 ± 0.7	1.1 ± 0.3	0.7 ± 0.3	1.3 ± 0.8	1.4 ± 0.4	0.7 ± 0.2	0.7 ± 0.3	0.3 ± 0.1	1.2 ± 0.5	1.2 ± 0.2
S604L	0.7 ± 2.1	2.1 ± 2.0	-0.5 ± 0.2	0.1 ± 2.3	2.0 ± 1.7	0.9 ± 0.9	1.9 ± 1.5	1.6 ± 0.9	0.3 ± 1.1	1.7 ± 1.3
L607F	0 ± 0.1	0.6 ± 1.0	0.3 ± 0.3	0.4 ± 0.4	1.6 ± 1.2	0.4 ± 0.3	0.4 ± 0.3	0.7 ± 0.3	0.9 ± 0.5	1.0 ± 0.5
Q612H	0 ± 0	0 ± 0	0 ± 0	0.7 ± 0.2	1.4 ± 0.6	0 ± 0	0 ± 0	0 ± 0	0.7 ± 0.3	0.9 ± 0.3
Y614C	0.3 ± 0.4	0.6 ± 0.2	0.5 ± 0.3	1.8 ± 0.5	2.2 ± 0.5	0.4 ± 0.3	0.8 ± 0.7	0.4 ± 0.3	1.8 ± 0.3	2.5 ± 0.3
R621Q	1.4 ± 0.9	1.6 ± 0.1	0.2 ± 0.3	2.8 ± 0.5	2.6 ± 0.3	1.4 ± 0.9	1.8 ± 1.1	0.2 ± 0.3	2.1 ± 0.5	2.3 ± 0.5
T624A	0.5 ± 0.2	0.6 ± 0.3	0.5 ± 0.2	-0.1 ± 0.1	0.1 ± 0.2	0.4 ± 0.1	1.1 ± 0.2	0.2 ± 0.1	-0.0 ± 0.1	0.6 ± 0.1
L625F	0 ± 0.2	-0.1 ± 0	0.3 ± 0.2	1.8 ± 1.2	1.1 ± 0.5	-0.1 ± 0.1	-0.1 ± 0.1	0 ± 0.1	1.2 ± 0.0	1.3 ± 0.2
L625I	0.1 ± 0.1	0 ± 0	0.1 ± 0.1	0.9 ± 0.3	0.9 ± 0.2	0 ± 0	0 ± 0	0 ± 0.1	0.7 ± 0.1	0.9 ± 0.4
A627V	0.3 ± 0	0.2 ± 0.1	0.4 ± 0.2	2.2 ± 0.5	2.6 ± 0.7	0.1 ± 0.1	0.1 ± 0.2	0.6 ± 0.6	2.6 ± 1.0	1.2 ± 0.3
K628R	-1.0 ± 0.1	-1.2 ± 0.1	0.8 ± 0.2	-0.9 ± 0.5	-1.2 ± 0	-1.0 ± 0	-0.9 ± 0.3	0.1 ± 0.4	-1.0 ± 0.4	-1.5 ± 0.2
K628N	1.8 ± 0.4	1.9 ± 0.4	1.1 ± 0.3	2.7 ± 0.6	2.7 ± 0.3	1.4 ± 0.4	0.9 ± 0.2	0.4 ± 0.6	1.9 ± 0.3	1.7 ± 0.2
I630M	0 ± 0	0 ± 0	0 ± 0	-0 ± 0.1	0 ± 0	0 ± 0	0 ± 0	0 ± 0.1	0.0 ± 0.1	-0.0 ± 0.0
N635S	1.2 ± 0.3	1.3 ± 0.2	0.4 ± 0.1	1.3 ± 0.5	1.5 ± 0.2	0.7 ± 0.2	0.5 ± 0	0.5 ± 0.5	1.9 ± 0.6	2.0 ± 0.2
F639L	0 ± 0	0 ± 0	0 ± 0.1	1.7 ± 0.1	1.8 ± 0.1	0 ± 0.1	0 ± 0	0.3 ± 0.4	1.3 ± 0.2	1.3 ± 0.2
E643Q	-1.6 ± 1.0	-0.7 ± 0.2	-0.3 ± 0.2	-0.5 ± 0.3	-0.5 ± 0.4	-0.3 ± 0	-0.2 ± 0.1	0 ± 0.4	0.5 ± 0.1	0.6 ± 0.1
D644A	0.1 ± 0.1	0.1 ± 0	0.6 ± 0.6	-0 ± 0.1	0.1 ± 0	0.8 ± 0.6	0.7 ± 0.3	0.2 ± 0.2	1.0 ± 0.5	1.0 ± 0.3
D644N	0 ± 0	0 ± 0.1	0.7 ± 0.6	-0.2 ± 0.2	-0.1 ± 0.1	0.3 ± 0.4	0.5 ± 0.3	0.3 ± 0.1	0.4 ± 0.2	0.5 ± 0.2
G647D	0 ± 0.1	0.1 ± 0	0 ± 0.1	0.9 ± 0.4	0.6 ± 0.3	0.1 ± 0.1	0.1 ± 0	0.3 ± 0.3	1.1 ± 1.5	0.1 ± 0.2
E651K	0 ± 0	0 ± 0	0 ± 0	0.1 ± 0	0.2 ± 0.1	0 ± 0	0 ± 0	0 ± 0	0.2 ± 0.1	0.3 ± 0.1
E654K	0 ± 0	0 ± 0	0 ± 0	-0 ± 0.1	0.1 ± 0.2	0 ± 0	-0.1 ± 0	0 ± 0	0.1 ± 0.2	0.2 ± 0.0
Q662K	0 ± 0	0 ± 0	0 ± 0	-0.1 ± 0.1	0 ± 0	0 ± 0	0 ± 0	0 ± 0	0.0 ± 0.0	-0.0 ± 0.0
E666D	0 ± 0	0 ± 0	0 ± 0	0.8 ± 0	1.0 ± 0	0 ± 0	0 ± 0	0 ± 0	0.8 ± 0.1	0.7 ± 0.0
E666K	0 ± 0	0 ± 0	0 ± 0	0 ± 0	0.4 ± 0.1	0 ± 0	0 ± 0	0 ± 0	0.0 ± 0.2	-0.2 ± 0.1
E666Q	0 ± 0	0 ± 0	0 ± 0	0.5 ± 0	0.9 ± 0	0 ± 0	0 ± 0	0 ± 0	0.5 ± 0.3	0.2 ± 0.1
D671G	0 ± 0	0 ± 0	0 ± 0	0.7 ± 0.2	0.5 ± 0.1	0 ± 0	0 ± 0	0 ± 0	0.5 ± 0.1	0.5 ± 0.3
T672M	0 ± 0	0 ± 0	0 ± 0	0.2 ± 0.1	0.5 ± 0.1	0 ± 0	0 ± 0	0 ± 0	0.4 ± 0.4	0.0 ± 0.1
E680D	0 ± 0	0 ± 0	0 ± 0	0.1 ± 0.1	0 ± 0.1	0 ± 0	0 ± 0	0 ± 0	0.1 ± 0.1	0.2 ± 0.2
E680Q	0 ± 0	0 ± 0	0 ± 0	0.3 ± 0.1	0.2 ± 0.1	0 ± 0	0 ± 0	0 ± 0	0.3 ± 0.1	0.1 ± 0.3
G686D	0 ± 0	0 ± 0	0 ± 0	1.9 ± 0.6	2.6 ± 0.2	0 ± 0	0 ± 0	0 ± 0	1.8 ± 0.1	2.1 ± 0.3
R687Q	0 ± 0	0 ± 0	0 ± 0	2.2 ± 0.2	1.9 ± 0.2	0 ± 0	0 ± 0	0 ± 0	2.3 ± 0.3	2.2 ± 0.2
E688Q	0 ± 0	0 ± 0	0 ± 0	0.2 ± 0	0.5 ± 0.1	0 ± 0	0 ± 0	0 ± 0	0.2 ± 0.3	0.2 ± 0.2
H693Y	0 ± 0	0 ± 0	0 ± 0	-0 ± 0.2	0.3 ± 0.2	0 ± 0	0 ± 0	0 ± 0.1	-0.2 ± 0.2	-0.1 ± 0.4
P713S	0 ± 0	0 ± 0	0 ± 0	0.9 ± 0	0.8 ± 0	0 ± 0	0 ± 0	0 ± 0	0.9 ± 0.0	0.8 ± 0.1
L714P	0 ± 0	0 ± 0	0 ± 0	7.5 ± 0.2	7.7 ± 0.3	0 ± 0	0 ± 0	0 ± 0	7.6 ± 0.1	7.5 ± 0.1
R716Q	0 ± 0	0 ± 0	0 ± 0	0.6 ± 0	0.6 ± 0	0 ± 0	0 ± 0	0 ± 0	0.6 ± 0.1	0.5 ± 0.1
R716W	0 ± 0	0 ± 0	0 ± 0	0.4 ± 0.1	0.3 ± 0	0 ± 0	0 ± 0	0 ± 0	0.4 ± 0.0	0.3 ± 0.0
L717F	0 ± 0	0 ± 0	0 ± 0	1.1 ± 0.1	1.0 ± 0.1	0 ± 0	0 ± 0	0 ± 0	1.1 ± 0.1	1.0 ± 0.1
D720G	0 ± 0	0 ± 0	0 ± 0	0.7 ± 0	0.6 ± 0	0 ± 0	0 ± 0	0 ± 0	0.6 ± 0.0	0.6 ± 0.0

Binding affinities and folding stabilities for all SynGAP–Ras and SynGAP–Rap variants (N = 214), where positive ΔΔG values indicate destabilization and negative values indicate stabilization, with all ΔG and ΔΔG values reported in kcal/mol. Table S1 includes the complete dataset for all variants, whereas Tables 1 and S2 present only the subset of variants exhibiting notable changes in binding affinity in either Rosetta or FoldX analyses. SD, standard deviation, represents between-replica variability, calculated as the sample SD of the mean ΔΔG values from the three WT replica ensembles for FoldX-MD and of the ΔΔG values from the three independently selected WT templates for Rosetta Flex ddG.

[1] FoldX-MD[4–6] $\Delta\Delta G_{bind}$ values were computed using *AnalyseComplex* on 180 WT simulation frames extracted from 200-ns simulations (first 20 ns discarded; one frame per ns thereafter). Three WT replica simulations were processed independently, and variant $\Delta\Delta G_{bind}$ values represent the mean and standard deviation relative to these three WT reference sets.

[2] Additional FoldX-MD binding-affinity $\Delta\Delta G_{bind}$ values were computed using AnalyseComplex on 200 frames from each of the three extended WT simulations (one frame per ns over the first 200 ns).

[3] Rosetta[7,8] $\Delta\Delta G_{bind}$ values were calculated using three WT template structures, each selected as the lowest-energy frame based on the MM/GBSA analysis of its corresponding replica simulation, and used as input for FlexddG. Reported values are the mean and standard deviation across the three templates.

[4] FoldX-MD $\Delta\Delta G_{fold}$ folding stability values were computed using *BuildModel* on 180 WT simulation frames as explained above for FoldX-MD (see 1).

[5] Additional FoldX-MD $\Delta\Delta G_{fold}$ values were computed using BuildModel on the same 200-frame WT ensembles described in 2.

Table S2. Binding affinities for SynGAP–Ras/Rap GTPase complexes.

Variant	SynGAP–Ras Complex				SynGAP–Rap Complex			
	FoldX-MD $\Delta\Delta G_{bind}$[1]	FoldX-MD %Average[2]	Rosetta $\Delta\Delta G_{bind}$[3]	Rosetta %Average[2]	FoldX-MD $\Delta\Delta G_{bind}$[1]	FoldX-MD %Average[2]	Rosetta $\Delta\Delta G_{bind}$[3]	Rosetta %Average[2]
R272W - Sim-1	1.0		0.7		0.6		0.6	
R272W - Sim-2	0.3	**0.7 ± 0.4**	1.2	1.0 ± 0.3	0.8	**0.7 ± 0.1**	0.9	0.6 ± 0.3
R272W - Sim-3	1.0		1.2		0.8		0.3	
E360K - Sim-1	0.8		0.7		1.0		0.1	
E360K - Sim-2	0.4	**0.6 ± 0.2**	-0.6	-0.4 ± 1.0	**0.7**	**1.0 ± 0.3**	0.2	0.3 ± 0.3
E360K - Sim-3	0.6		-1.3		**1.4**		0.6	
R401Q - Sim-1†	2.7		0.9		4.5		1.6	
R401Q - Sim-2†	0.9	**1.6 ± 0.9**	0.0	0.4 ± 0.5	**3.6**	**3.9 ± 0.6**	0.8	1.3 ± 0.4
R401Q - Sim-3†	1.2		0.3		3.4		1.4	
R401W - Sim-1	1.9		0.3		**3.6**		0.8	
R401W - Sim-2	0.7	**1.1 ± 0.7**	-0.4	-0.1 ± 0.4	2.9	**3.0 ± 0.6**	0.3	0.6 ± 0.3
R401W - Sim-3	0.6		-0.2		2.4		0.7	
N440I - Sim-1	0.0		-0.5		**0.8**		0.1	
N440I - Sim-2	0.0	0.0 ± 0.0	-0.5	-0.5 ± 0.1	**0.3**	**0.5 ± 0.3**	-0.1	0.0 ± 0.1
N440I - Sim-3	0.0		-0.4		0.4		0.0	
K442R - Sim-1	-0.6		-0.1		**-1.1**		0.3	
K442R - Sim-2	-0.5	**-0.6 ± 0.1**	0.0	0.0 ± 0.1	**-1.1**	**-1.1 ± 0.0**	0.4	0.5 ± 0.3
K442R - Sim-3	-0.8		0.0		**-1.2**		0.9	
R479Q - Sim-1†	0.4		-0.1		**2.0**		0.2	
R479Q - Sim-2†	0.3	0.3 ± 0.2	0.0	0.0 ± 0.1	**1.7**	**1.8 ± 0.2**	0.4	0.2 ± 0.2
R479Q - Sim-3†	0.1		0.0		**1.7**		-0.0	
R479W - Sim-1	0.2		-0.1		1.6		0.0	
R479W - Sim-2	0.0	0.0 ± 0.2	-0.1	-0.1 ± 0.1	1.5	**1.5 ± 0.1**	0.1	0.1 ± 0.1
R479W - Sim-3	-0.2		-0.2		**1.4**		0.1	
H481D - Sim-1	1.1		0.7		0.7		0.2	
H481D - Sim-2	1.0	**1.1 ± 0.1**	0.4	0.6 ± 0.2	0.8	**0.9 ± 0.2**	0.2	0.6 ± 0.6
H481D - Sim-3	1.2		0.7		1.1		1.3	
F484A - Sim-1	0.1		0.0		0		0.0	
F484A - Sim-2	0.0	0.1 ± 0.0	0.0	0.0 ± 0.0	**0.0**	0.0 ± 0.0	0.0	0.0 ± 0.1
F484A - Sim-3	0.1		-0.0		**0**		-0.1	
R485L-F484A - Sim-1	-0.1		-0.7		-0.2		0.4	
R485L-F484A - Sim-2	0.0	-0.1 ± 0.1	-0.1	-0.6 ± 0.4	0.0	-0.1 ± 0.1	-0.6	-0.1 ± 0.5
R485L-F484A - Sim-3	0.0		-0.8		**-0.2**		-0.1	
R485L - Sim-1	-0.2		-0.7		**-0.3**		0.4	
R485L - Sim-2	0.0	-0.1 ± 0.1	-0.5	-0.7 ± 0.2	**-0.1**	-0.2 ± 0.1	-0.6	-0.1 ± 0.5
R485L - Sim-3	-0.1		-0.9		**-0.2**		-0.2	
R485K - Sim-1	0.7		0.7		**0.5**		0.9	
R485K - Sim-2	0.5	**0.7 ± 0.2**	0.7	0.8 ± 0.2	0.5	**0.5 ± 0.0**	0.2	0.7 ± 0.4
R485K - Sim-3	0.9		1.0		**0.4**		0.9	
R485H - Sim-1	1.7		0.1		**0.8**		0.5	
R485H - Sim-2	1.3	**2.0 ± 0.9**	0.7	0.2 ± 0.5	0.7	**0.7 ± 0.1**	-0.3	0.1 ± 0.4
R485H - Sim-3	3.1		-0.3		**0.6**		0.2	
R485P - Sim-1	1.9		0.1		**1.0**		1.2	
R485P - Sim-2	2.0	**2.1 ± 0.3**	1.9	1.1 ± 0.9	**1.2**	**1.0 ± 0.2**	0.5	0.8 ± 0.4
R485P - Sim-3	2.4		1.2		**0.8**		0.7	
E486K - Sim-1†	0.9		0.6		0.3		0.3	
E486K - Sim-2†	0.7	**0.9 ± 0.2**	0.3	0.3 ± 0.2	0.5	0.3 ± 0.2	0.1	0.0 ± 0.3
E486K - Sim-3†	1.2		0.2		0.1		-0.3	
N487K - Sim-1	1.5		0.7		0.6		0.3	
N487K - Sim-2	0.7	**0.8 ± 0.7**	-0.5	0.1 ± 0.6	0.3	**0.5 ± 0.2**	2.0	0.5 ± 1.4
N487K - Sim-3	0.1		0.1		0.7		-0.7	
N487T - Sim-1	1.7		-0.1		0.3		-0.2	
N487T - Sim-2	1.0	**1.1 ± 0.5**	-0.3	-0.3 ± 0.2	0.4	0.4 ± 0.2	-0.5	-0.3 ± 0.2
N487T - Sim-3	0.7		-0.5		0.6		0.0	
K492E - Sim-1†	2.1		1.2		2.1		0.7	
K492E - Sim-2†	1.6	**1.7 ± 0.4**	1.1	**1.0 ± 0.2**	1.3	**1.4 ± 0.6**	0.7	0.5 ± 0.3
K492E - Sim-3†	1.2		0.8		0.9		0.2	
R596H - Sim-1†	1.6		0.2		1.1		0.8	
R596H - Sim-2†	1.5	**1.3 ± 0.4**	-0.2	-0.1 ± 0.3	1.2	**1.2 ± 0.1**	0.3	0.5 ± 0.3
R596H - Sim-3†	0.9		-0.3		1.2		0.2	

Table S2. Continues

Variant	SynGAP–Ras Complex				SynGAP–Rap Complex			
	FoldX-MD $\Delta\Delta G_{bind}$[1]	FoldX-MD %Average[2]	Rosetta $\Delta\Delta G_{bind}$[3]	Rosetta %Average[2]	FoldX-MD $\Delta\Delta G_{bind}$[1]	FoldX-MD %Average[2]	Rosetta $\Delta\Delta G_{bind}$[3]	Rosetta %Average[2]
P600V - Sim-1	0.5		-0.1		0.2		-0.0	
P600V - Sim-2	1.1	**1.0 ± 0.4**	-0.4	-0.2 ± 0.2	0.8	**0.7 ± 0.4**	0.5	0.2 ± 0.3
P600V - Sim-3	1.3		0.0		1.0		0.0	
M603I - Sim-1	0.8		1.0		0.5		0.5	
M603I - Sim-2	1.0	**1.1 ± 0.3**	0.4	0.7 ± 0.3	0.8	**0.7 ± 0.2**	0.2	0.3 ± 0.1
M603I - Sim-3	1.4		0.6		0.8		0.3	
S604L - Sim-1†	-0.4		-0.7		0.7		1.9	
S604L - Sim-2†	2.1	**1.4 ± 1.6**	-0.3	-0.5 ± 0.2	1.4	**1.4 ± 0.7**	2.3	1.6 ± 0.9
S604L - Sim-3†	2.6		-0.7		2.1		0.5	
L607F - Sim-1	0.9		0.0		0.5		0.6	
L607F - Sim-2	0.0	0.3 ± 0.5	0.7	0.3 ± 0.3	0.2	0.4 ± 0.2	0.5	0.7 ± 0.3
L607F - Sim-3	0.1		0.2		0.5		1.0	
Y614C - Sim-1	0.3		0.1		1.0		0.4	
Y614C - Sim-2	0.4	0.4 ± 0.2	0.7	0.5 ± 0.3	0.8	**0.6 ± 0.5**	0.7	0.4 ± 0.3
Y614C - Sim-3	0.6		0.8		0.1		0.1	
R621Q - Sim-1†	1.1		0.1		1.4		0.5	
R621Q - Sim-2†	1.5	**1.5 ± 0.4**	0.5	0.2 ± 0.3	0.8	**1.6 ± 0.9**	0.2	0.2 ± 0.3
R621Q - Sim-3†	1.9		-0.2		2.6		-0.1	
A627V - Sim-1	0.3		0.6		0.1		1.3	
A627V - Sim-2	0.2	0.2 ± 0.0	0.3	0.4 ± 0.2	0	0.1 ± 0.1	0.4	0.6 ± 0.6
A627V - Sim-3	0.2		0.4		0.2		0.1	
K628R - Sim-1	-1.1		0.8		-0.9		0.0	
K628R - Sim-2	-1.2	**-1.1 ± 0.0**	1.0	0.8 ± 0.2	-1.1	**-1.0 ± 0.2**	0.6	0.1 ± 0.4
K628R - Sim-3	-1.1		0.7		-0.8		-0.2	
K628N - Sim-1	1.8		0.9		0.9		0.3	
K628N - Sim-2	2.3	**1.9 ± 0.4**	1.3	1.1 ± 0.3	1.4	**1.1 ± 0.3**	1.1	0.4 ± 0.6
K628N - Sim-3	1.5		0.9		1.1		-0.1	
N635S - Sim-1†	0.9		0.4		0.5		0.6	
N635S - Sim-2†	1.3	**1.2 ± 0.3**	0.5	0.4 ± 0.1	0.7	**0.6 ± 0.1**	0.0	0.5 ± 0.5
N635S - Sim-3†	1.5		0.3		0.7		1.0	
E643Q - Sim-1	-0.9		-0.2		-0.2		-0.3	
E643Q - Sim-2	-1.8	**-1.1 ± 0.6**	-0.2	-0.3 ± 0.2	-0.3	-0.2 ± 0.1	0.1	0.0 ± 0.4
E643Q - Sim-3	-0.7		-0.6		-0.2		0.4	
D644N - Sim-1	0.0		1.1		0.4		0.2	
D644N - Sim-2	0.0	0.0 ± 0.0	0.9	0.7 ± 0.6	0.3	0.4 ± 0.2	0.4	0.3 ± 0.1
D644N - Sim-3	-0.1		0.0		0.7		0.3	
D644A - Sim-1	0.0		1.1		0.6		0.5	
D644A - Sim-2	0.0	0.0 ± 0.0	0.8	0.6 ± 0.5	0.5	**0.7 ± 0.3**	0.1	0.2 ± 0.2
D644A - Sim-3	0.0		0.0		1.1		0.1	

Binding affinities for the WT and missense SynGAP–Ras and SynGAP–Rap complexes, where positive ΔΔG values indicate destabilization and negative values indicate stabilization, with all ΔG and ΔΔG values reported in kcal/mol. Variants included in this table are those that exceeded the energetic thresholds in either Rosetta or FoldX. Numerical values are shown in bold for mildly destabilizing or stabilizing effects ($\Delta\Delta G_{bind} \geq 0.5$ or ≤ -0.5 kcal/mol), and bolded/underlined for significant perturbations ($\Delta\Delta G_{bind} \geq 1.0$ or ≤ -1.0 kcal/mol, or $\Delta\Delta G_{fold} \geq 3$ or ≤ -3 kcal/mol).

[1] FoldX-MD $\Delta\Delta G_{bind}$ values were computed using *AnalyseComplex* on a combined set of 380 WT simulation frames per replica, comprising the original 180 frames from the 200-ns simulations (first 20 ns discarded; one frame per ns thereafter) together with an additional 200 frames from the restarted and extended WT simulations. The three WT replica datasets were processed independently.

[2] Values are reported as mean ± SD. SD represents between-replica variability and was calculated as the sample SD of the three replica mean ΔΔG values for FoldX-MD and of the three template-derived ΔΔG values for Rosetta Flex ddG..

[3] Rosetta $\Delta\Delta G_{bind}$ values were calculated using three WT template structures, each selected as the lowest-energy frame based on the MM/GBSA analysis of its corresponding replica simulation and used as input for FlexddG.

Grey: Strong/reproducible effect: all three replicas show the same direction and ≥2/3 exceed the predefined energetic threshold.

Sky blue: Moderate effect: all three replicas show the same direction, but the threshold is not consistently exceeded.

Blue: Uncertain effect: replicas disagree in the direction of the energetic change.

† Missense variants correspond to ClinVar-annotated substitutions, classified as VUS (R401Q, E486K, K492E, S604L, N635S) or as likely benign (R479Q, R596H, R621Q).

Table S3. Percentage of coordinated water at the SynGAP-induced Ras/Rap-GTPase active-site between catalytic glutamine and GTP

Variant	SynGAP–Ras Complex	SynGAP–Rap Complex
	% of Coordinated Water Bridge[1]	% of Coordinated Water Bridge[1]
WT*	89.3 ± 5.1	55.1 ± 39.0
WT	90.4 ± 4.6	56.4 ± 20.9
R272W	89.8 ± 9	61.3 ± 16.9
E360K	98.9 ± 0.2	42.4 ± 21
R401Q	94.8 ± 1.9	**35.4 ± 10.3**
N440I	**66.4 ± 22.4**	**40.2 ± 17.7**
K442R	91.7 ± 9.8	50 ± 17.5
R479Q	96.2 ± 0.8	57.4 ± 21
H481D	**64.6 ± 45.6**	**78.2 ± 4.1**
F484A	**75 ± 32.6**	58.6 ± 12
R485P	**57 ± 39.8**	**35.4 ± 25.4**
R485L/F484A	**55.8 ± 14.1**	**39.4 ± 15.9**
R485K	**65.8 ± 46.5**	**20.6 ± 16.4**
R485L	**72.7 ± 22.3**	51.6 ± 16.9
E486K	**66.4 ± 43.7**	**70.5 ± 9.3**
N487K	**66.4 ± 41.5**	62.9 ± 5.3
N487T	89.5 ± 6.8	53.5 ± 24.9
K492E	90.1 ± 5.4	49.1 ± 22.2
R596H	**60 ± 20.1**	**14 ± 14.8**
P600V	95.9 ± 4	**46.1 ± 35.1**
M603I	80.8 ± 13.1	**42.5 ± 26.4**
S604L	81.1 ± 1.9	**41.8 ± 20.3**
L607F	90.2 ± 12.6	53.6 ± 37.2
Y614C	97.7 ± 0.2	47.2 ± 31.5
R621Q	**73.7 ± 32.3**	55.8 ± 13
A627V	98 ± 0.7	53.6 ± 29.3
K628N	**60.7 ± 27.9**	**42.6 ± 0.4**
N635S	81.7 ± 6.4	**31.1 ± 25.3**
D644A	92.9 ± 5	**37.3 ± 37.1**

Percentage of water molecule being coordinated at the catalytic position between GTP and residues Gln61 (SynGAP–Ras) or Gln63 (SynGAP–Rap) across three replica MD simulation trajectories (Fig. 4A-B). Values represent averages over three independent replicas. Values differing from the WT by more than 10% are highlighted in bold. H-bond distances and angles were computed using the *hbond bridgeout* in CPPTRAJ[2], considering the water molecule closest to the GTP γ-phosphate at each simulation frame. Water behaviour was evaluated using:

[1] Coordinated-water criterion: Water classified as coordinated only when simultaneously satisfying both the H-bond angle and distance requirements (≤3.6 Å heavy-atom distance and ≥135° Acceptor–H–Donor angle) between the catalytic Gln and GTP.

*WT refers to the original WT system from the initial 200-ns simulations, prior to the subsequent 100-ns WT/variant simulation set.

Table S4. MM/GBSA Binding Free Energies for SynGAP–Ras and SynGAP–Rap GTPase complexes.

Variant	SynGAP-Ras Complex				SynGAP-Rap Complex			
	MMGSA ΔG_{bind} (100ns)[1]	MMGSA $\Delta\Delta G_{bind}$ [2]	MMGSA ΔG_{bind} (500ns)[3]	MMGBA $\Delta\Delta G_{bind}$ [2]	MMGSA ΔG_{bind} (100ns)[1]	MMGSA $\Delta\Delta G_{bind}$ [2]	MMGSA ΔG_{bind} (500ns)[3]	MMGBA $\Delta\Delta G_{bind}$ [2]
WT	-101.7 ± 10.2	N/A	-64.4 ± 2.1	N/A	-92.3 ± 19.7	N/A	-68.7 ± 4.3	N/A
R272W	-101.4 ± 18.4	0.3	N/A	N/A	-87.8 ± 24.7	4.5	N/A	N/A
E360K	-85.5 ± 7.9	16.2	N/A	N/A	-96.6 ± 8.4	-4.3	N/A	N/A
R401Q†	-96.2 ± 4.3	5.5	-68.5 ± 18.9	-4.1	-67.6 ± 9.1	24.7	-46.3 ± 13.3	22.4
N440I	-90.4 ± 12.5	11.3	N/A	N/A	-81.6 ± 7.9	10.7	N/A	N/A
K442R	-99.2 ± 19.1	2.5	N/A	N/A	-103.7 ± 9.0	-11.4	N/A	N/A
R479Q†	-83.0 ± 2.6	18.7	-65.7 ± 4.8	-1.3	-74.8 ± 3.7	17.5	-60.3 ± 6.6	8.4
H481D	-79.5 ± 6.4	22.2	-63.6 ± 24.1	0.8	-87.0 ± 26.5	5.3	-66.7 ± 13.8	2
F484A	-100.8 ± 25.8	0.9	N/A	N/A	-87.8 ± 13.2	4.5	N/A	N/A
R485L-F484A	-97.8 ± 17.9	3.9	-79.7 ± 12.9	-15.3	-95.8 ± 18.1	-3.5	-77.7 ± 22.4	-9
R485L	-87.2 ± 5.4	14.5	N/A	N/A	-88.4 ± 8.2	3.9	N/A	N/A
R485K	-76.9 ± 10.7	24.8	-53.2 ± 11.9	11.2	-71.0 ± 1.8	21.3	-50.2 ± 8.1	18.5
R485P	-84.0 ± 15.1	17.7	-64.7 ± 12.2	-0.3	-69.7 ± 7.3	22.6	-56.6 ± 4.5	12.1
E486K†	-99.8 ± 36.6	1.9	-80.3 ± 36.1	-15.9	-98.1 ± 17.1	-5.8	-65.7 ± 14	3
N487K	-96.1 ± 25.4	5.6	-72.6 ± 16	-8.2	-87.1 ± 5.2	5.2	-75.6 ± 9.8	-6.9
N487T	-95.1 ± 17.6	6.6	-73.4 ± 16.3	-9	-93.8 ± 25.4	-1.5	-73.1 ± 8.9	-4.4
K492E†	-93.1 ± 6.1	8.6	-59.2 ± 24	5.2	-77.6 ± 14.2	14.7	-68.7 ± 3.7	0
R596H†	-103.7 ± 5.0	-2	-67 ± 17.6	-2.6	-94.4 ± 11.5	-2.1	-67.7 ± 7.7	1
P600V	-93.6 ± 19.2	8.1	-69.9 ± 17	-5.5	-91.1 ± 6.2	1.2	-64.4 ± 6.8	4.3
M603I	-88.5 ± 10.7	13.2	N/A	N/A	-88.7 ± 7.9	3.6	N/A	N/A
S604L†	-92.5 ± 25.8	9.2	-63.9 ± 12.3	0.5	-81.8 ± 6.2	10.5	-67.6 ± 5.2	1.1
L607F	-104.6 ± 10.1	-2.9	N/A	N/A	-81.4 ± 18.4	10.9	N/A	N/A
Y614C	-95.2 ± 17.8	6.5	N/A	N/A	-110.5 ± 1.8	-18.2	N/A	N/A
R621Q†	-80.3 ± 9.4	21.4	N/A	N/A	-87.5 ± 11.5	4.8	N/A	N/A
A627V	-93.2 ± 8.6	8.5	N/A	N/A	-89.5 ± 10.8	2.8	N/A	N/A
K628N	-83.1 ± 14.6	18.6	-61.1 ± 13.3	3.3	-81.8 ± 4.4	10.5	-78.3 ± 6.2	-9.6
N635S†	-95.3 ± 21.0	6.4	N/A	N/A	-88.2 ± 3.6	4.1	N/A	N/A
D644A	-87.5 ± 6.0	14.2	N/A	N/A	-108.0 ± 10.6	-15.7	N/A	N/A

[1] MM/GBSA ΔG_{bind} binding free energies in Kcal/mol were computed for each frame for 100-ns simulations using AMBER MMPBSA.py. Values are reported as the mean across three replica simulations ± the standard deviation between replicas.

[2] The reported value represents $\Delta\Delta G_{bind}$ calculated as $\Delta G_{bind-WT}$ - $\Delta G_{bind-variant}$

[3] MM/GBSA ΔG_{bind} MM/GBSA ΔG binding free energies for the 500-ns simulations were calculated and reported as described in 1.

MM/GBSA results are interpreted qualitatively; the improved convergence and lower between-replica SD of the WT at 500 ns provide a stable reference for comparison, whereas the greater variability among several variant replicas limits quantitative assessment of subtle energetic differences.

† See Table 1 for more details.

Table S5. Binding affinity and stability of GTP within SynGAP–Ras and SynGAP–Rap GTPase complexes.

Variant	SynGAP-Ras Complex				SynGAP-Rap Complex			
	MMGSA ΔG_{bind}[1]	MMGSA $\Delta\Delta G_{bind}$[2]	RMSF[3]	RMSD[4]	MMGSA ΔG_{bind}[1]	MMGSA $\Delta\Delta G_{bind}$[2]	RMSF[3]	RMSD[4]
WT	-248.4 ± 14.9	N/A	2.4 ± 0.8	0.53 ± 0.15	-300.7 ± 2.2	N/A	1.5 ± 0.2	0.36 ± 0.02
R272W	-275.0 ± 25.3	**-26.6**	3.1 ± 0.9	**0.86 ± 0.16**	-300.6 ± 4.3	0.1	1.6 ± 0.3	0.44 ± 0.13
E360K	-280.1 ± 32.3	**-31.7**	1.9 ± 0.5	0.63 ± 0.13	-295.0 ± 6.6	5.7	**2.4 ± 0.5**	**0.61 ± 0.16**
R401Q†	**-264.6 ± 14.4**	**-16.2**	2.3 ± 0.4	0.55 ± 0.06	**-285.0 ± 7.7**	**15.7**	**2.5 ± 0.8**	0.49 ± 0.08
N440I	-254.9 ± 25.4	-6.5	3.2 ± 0.5	0.63 ± 0.09	-296.7 ± 10.3	4	**2.1 ± 0.6**	**0.57 ± 0.17**
K442R	**-281.1 ± 23.4**	**-32.7**	2.3 ± 0.8	0.65 ± 0.07	-300.5 ± 0.7	0.2	1.5 ± 0.1	0.39 ± 0.02
R479Q†	-252.8 ± 13.9	-4.4	2.7 ± 0.7	0.63 ± 0.04	-303.8 ± 11.6	-3.1	**2.4 ± 0.7**	**0.61 ± 0.02**
H481D	**-287.5 ± 18.3**	**-39.1**	2.1 ± 0.3	0.56 ± 0.19	-294.3 ± 6.4	6.4	1.6 ± 0	**0.60 ± 0.17**
F484A	-237.8 ± 15.6	10.6	1.9 ± 0.6	0.61 ± 0.18	-296.7 ± 1.2	4	**2.3 ± 0.8**	**0.61 ± 0.12**
R485L-F484A	**-215.9 ± 25.2**	**32.5**	**3.7 ± 0.4**	0.72 ± 0.06	-238.9 ± 5.2	**61.8**	**2.5 ± 0.9**	**0.63 ± 0.04**
R485L	**-222.7 ± 5.2**	**25.7**	**3.5 ± 0.1**	0.65 ± 0.07	-244.3 ± 6.2	**56.4**	**2.7 ± 0.6**	**0.64 ± 0.09**
R485K	**-297.8 ± 1.9**	**-49.4**	2.4 ± 0.3	0.65 ± 0.17	-288.7 ± 29.5	**12**	**2.2 ± 0.5**	**0.67 ± 0.11**
R485P	**-224.7 ± 11.9**	**23.7**	**3.5 ± 0.4**	0.68 ± 0.12	**-228.4 ± 9.0**	**72.3**	**2.8 ± 0.2**	**0.72 ± 0.11**
E486K†	**-288.5 ± 17.9**	**-40.1**	2.2 ± 0.8	0.56 ± 0.21	**-290.3 ± 7.6**	**10.4**	**2.1 ± 0.6**	0.52 ± 0.19
N487K	-262.5 ± 15.2	-14.1	2.6 ± 0.3	0.59 ± 0.06	-306.1 ± 5.3	-5.4	1.6 ± 0.1	0.47 ± 0.08
N487T	-239.5 ± 22.2	8.9	2.2 ± 0.7	0.47 ± 0.06	-303.9 ± 3.8	-3.2	**2.4 ± 0.7**	**0.62 ± 0.18**
K492E†	-262.5 ± 24.0	-14.1	2.2 ± 0.8	0.58 ± 0.26	-294.4 ± 9.9	6.3	**2.5 ± 0.8**	0.53 ± 0.13
R596H†	-270.9 ± 27.4	-22.5	2.9 ± 0.7	0.53 ± 0.05	-302.7 ± 4.5	-2	**1.9 ± 0**	**0.60 ± 0.08**
P600V	-258.9 ± 25.3	-10.5	2 ± 0.9	0.55 ± 0.04	-285.4 ± 9.0	**15.3**	**2.7 ± 0.5**	**0.67 ± 0.08**
M603I	-254.3 ± 33.2	-5.9	1.6 ± 0.2	0.55 ± 0.16	-302.9 ± 3.6	-2.2	1.4 ± 0.1	0.43 ± 0.05
S604L†	-255.6 ± 32.4	-7.2	2 ± 0.5	0.45 ± 0.03	**-285.6 ± 9.1**	15.1	2.2 ± 0.6	**0.68 ± 0.18**
L607F	-239.6 ± 6.5	8.8	1.9 ± 0.4	0.47 ± 0.11	-299.2 ± 2.4	1.5	1.9 ± 0.2	0.55 ± 0.10
Y614C	-265.1 ± 25.2	-16.7	2.5 ± 0.7	0.71 ± 0.24	-301.0 ± 2.7	-0.3	1.3 ± 0	0.43 ± 0.09
R621Q†	-267.5 ± 35.5	-19.1	1.6 ± 0.2	0.68 ± 0.16	-298.6 ± 5.8	2.1	1.6 ± 0	0.42 ± 0.08
A627V	-268.9 ± 21.6	-20.5	2.4 ± 0.3	0.46 ± 0.04	-295.5 ± 12.0	5.2	2.2 ± 0.7	**0.67 ± 0.20**
K628N	**-224.9 ± 1.4**	23.5	2.6 ± 1	0.60 ± 0.13	-298.6 ± 2.0	2.1	2.4 ± 0.7	**0.75 ± 0.19**
N635S†	**-276.3 ± 23.7**	-27.9	2.4 ± 0.8	0.60 ± 0.18	-309.7 ± 5.0	-9	1.7 ± 0.2	0.39 ± 0.06
D644A	-270.0 ± 22.8	-21.6	2.9 ± 0.5	0.67 ± 0.15	-302.4 ± 7.4	-1.7	2.6 ± 0.6	0.56 ± 0.02

Binding free energies and structural fluctuation metrics computed for each frame for each frame from 100-ns simulations for the GTP molecule in the WT and missense variant SynGAP–Ras and SynGAP–Rap complexes. ΔG and ΔΔG values are reported in kcal/mol while RMSF (root-mean-square fluctuation) and RMSD (root-mean-square deviation) in Å. For RMSF and RMSD, positive values indicate increased fluctuations or structural movement whereas negative value indicate substrate rigidification. While for MM/GBSA a positive ΔΔG reflects decreased binding affinity and a negative ΔΔG reflects increased affinity. Values are reported as the mean across three replica simulations ± the standard deviation between replicas. Values are highlighted in bold when the variant mean ± SD falls outside the WT mean ± SD for that metric, with WT and variant statistics computed from three independent simulation replicas.

[1] MM/GBSA ΔG_{bind} binding free energies of GTP were computed using AMBER *MMPBSA.py*, with averages reported across three replica simulations.

[2] The reported value represents $\Delta\Delta G_{bind}$ calculated as $\Delta G_{bind-WT}$ - $\Delta G_{bind-variant}$

[3] RMSF of GTP heavy atoms was averaged across three replica simulations.

[4] RMSD of GTP heavy atoms relative to the starting structure was averaged across three replica simulations.

† See Table 1 for more details.

# References.

1. Scrima A, Thomas C, Deaconescu D, Wittinghofer A. The Rap-RapGAP complex: GTP hydrolysis without catalytic glutamine and arginine residues. *EMBO Journal*. 2008;27(7):1145-1153. doi:10.1038/emboj.2008.30

2. Case DA, Aktulga HM, Belfon K, et al. AmberTools. *J Chem Inf Model*. 2023;63(20):6183-6191. doi:10.1021/acs.jcim.3c01153

3. Laskowski RA, Swindells MB. LigPlot+: Multiple ligand-protein interaction diagrams for drug discovery. *J Chem Inf Model*. 2011;51(10):2778-2786. doi:10.1021/ci200227u

4. Schymkowitz J, Borg J, Stricher F, Nys R, Rousseau F, Serrano L. The FoldX web server: An online force field. *Nucleic Acids Res*. 2005;33(SUPPL. 2). doi:10.1093/nar/gki387

5. Miller CR, Johnson EL, Burke AZ, et al. Initiating a watch list for Ebola virus antibody escape mutations. *PeerJ*. 2016;2016(2). doi:10.7717/peerj.1674

6. Ali AE, Li LL, Courtney MJ, Pentikäinen OT, Postila PA. Atomistic simulations reveal impacts of missense mutations on the structure and function of SynGAP1. *Brief Bioinform*. 2024;25(6). doi:10.1093/bib/bbae458

7. Sora V, Laspiur AO, Degn K, et al. RosettaDDGPrediction for high-throughput mutational scans: From stability to binding. *Protein Science*. 2023;32(1). doi:10.1002/pro.4527

8. Barlow KA, Ó Conchúir S, Thompson S, et al. Flex ddG: Rosetta Ensemble-Based Estimation of Changes in Protein-Protein Binding Affinity upon Mutation. *Journal of Physical Chemistry B*. 2018;122(21):5389-5399. doi:10.1021/acs.jpcb.7b11367
